# The ULK1-NCOA3 axis restrains de novo lipogenesis and prevents diet-induced steatohepatitis and fibrosis in mice

**DOI:** 10.1172/JCI191101

**Published:** 2026-04-02

**Authors:** Young Do Koo, Romilia Tatiana Castillo, Asha Sukumaran Nair, Michael Garneau, Chad Gochee, Zachary V. Campbell, Tashya Shreyas Vakil, Jua Ha, Alex Marti, Jamie Soto, Debajyoti Das, Nuria Martinez-Lopez, Shipra Sharma, Yennifer Delgado, Callie Phung, Immy A. Ashley, Edmund D. Kapelczak, Rashel Jacobo, Eric T. Weatherford, Dao-Fu Dai, Jihane N. Benhammou, Andrea G. Marshall, Antentor Hinton, Ling Yang, Renata O. Pereira, Tara TeSlaa, Mehdi Bouhaddou, Rajat Singh, E. Dale Abel

**Affiliations:** 1 Division of Endocrinology, Diabetes and Metabolism, Department of Medicine, David Geffen School of Medicine and UCLA Health, UCLA, Los Angeles, California, USA.; 2 Fraternal Order of Eagles Diabetes Research Center, Roy J. and Lucille A. Carver College of Medicine, and; 3 Mouse Metabolic Phenotyping Core, Carver College of Medicine, University of Iowa, Iowa City, Iowa, USA.; 4 Center for Translational Science, Florida International University, Port Saint Lucie, Florida, USA.; 5 Vatche and Tamar Manoukian Division of Digestive Diseases, David Geffen School of Medicine,; 6 Comprehensive Liver Research Center,; 7 Department of Microbiology, Immunology, and Molecular Genetics (MIMG),; 8 Institute for Quantitative and Computational Biosciences, and; 9 Department of Molecular and Medical Pharmacology, UCLA, Los Angeles, California, USA; 10 Department of Pathology, Johns Hopkins University School of Medicine, Baltimore, Maryland, USA.; 11 Veterans’ Affairs Greater Los Angeles Healthcare System, Los Angeles, California, USA.; 12 Department of Molecular Physiology and Biophysics, Vanderbilt University, Nashville, Tennessee, USA.; 13 Department of Anatomy and Cell Biology, University of Iowa Carver College of Medicine, Iowa City, Iowa, USA.

**Keywords:** Cell biology, Endocrinology, Hepatology, Autophagy, Obesity

## Abstract

Metabolic dysfunction–associated steatotic liver disease (MASLD) and metabolic dysfunction–associated steatohepatitis (MASH) are leading causes of cirrhosis and hepatocellular carcinoma. Defects in autophagy contribute to the development of MASLD; however, the role of Unc-51–like autophagy-activating kinase 1 (ULK1) in the pathophysiology of MASLD remains unclear. Herein, we show that ULK1, a serine/threonine kinase and core autophagy protein, is significantly repressed in human MASH livers, and that hepatocyte-specific loss of ULK1 promotes, unexpectedly, hepatic steatosis and progression to liver fibrosis, without affecting basal autophagy flux. Phospho-proteomics identified the transcriptional coactivator NCOA3 as a downstream phospho-target of ULK1. Mechanistically, ULK1 phosphorylates NCOA3 to repress its transcriptional activity and restrain the CREB/CBP-mediated de novo lipogenic program. Accordingly, a phosphorylation-deficient NCOA3 mutant drives CREB/CBP-mediated lipogenesis, whereas genetic or pharmacological NCOA3 inhibition prevents steatosis, hepatic inflammation, and profibrotic signaling. Hence, ULK1-mediated NCOA3 phosphorylation is a fundamental and druggable checkpoint against the entire MASLD spectrum.

## Abstract 2

ULK1 suppresses hepatic steatohepatitis by regulating lipid metabolism and anitoxidant pathways via NCOA3 phosphorylation, offering a potential drug target to prevent fatty liver progression and fibrosis.

## Graphical abstract

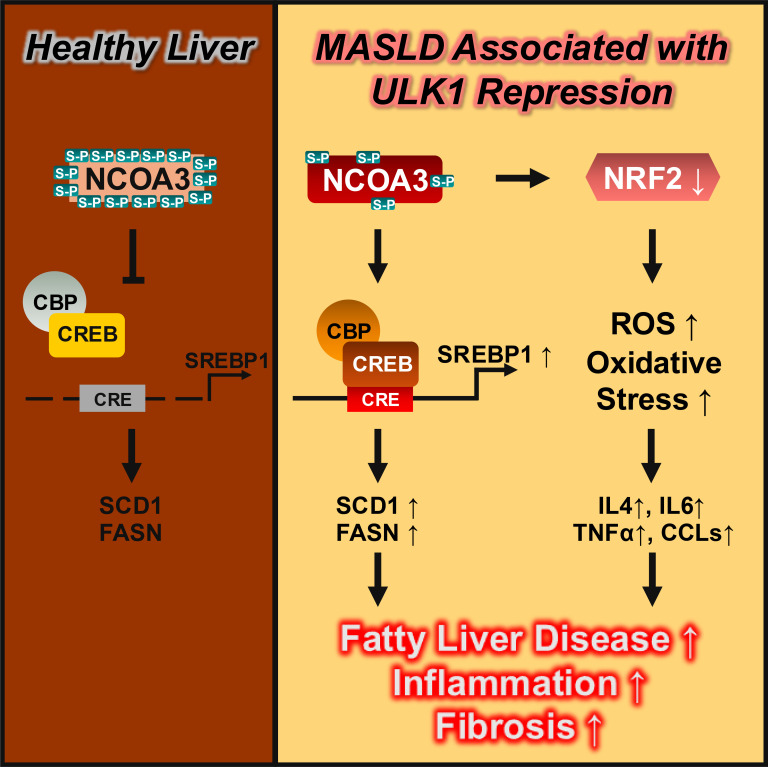

## Introduction

Metabolic dysfunction–associated steatotic liver disease (MASLD) and insulin resistance, now at epidemic proportions globally, both share a complex and bidirectional relationship in the etiology of obesity-associated chronic disease (1). The highly heterogeneous MASLD spectrum progresses, in a subset of individuals, to metabolic dysfunction–associated steatohepatitis (MASH), cirrhosis, and hepatocellular carcinoma (2); however, the mechanisms for this clinical heterogeneity and variable progression remain unclear (3, 4). Autophagy, a lysosomal quality-control mechanism, may play a key role in protecting against MASLD through multiple mechanisms, including lipid clearance (5). Autophagy is induced under conditions of nutrient deprivation, leading to the degradation of organelles and proteins, which are recycled to maintain cellular homeostasis (6). Even under basal states, autophagy maintains cellular health and function (7). Autophagy is systemically impaired during obesity (8), and reduction of hepatic autophagy has been shown to contribute to MASLD (5). Stimulation of hepatic autophagy has been proposed as a therapeutic approach to ameliorate the MASLD spectrum, as illustrated by studies using targeted overexpression of various core ATG and lysosomal genes (8).

Unc-51–like autophagy-activating kinase 1 (ULK1)/ATG1 is the only kinase within the ATG protein family that regulates autophagy (9), among its other autophagy-independent roles. During fasting states, ULK1 is activated through its phosphorylation by AMPK, whereas nutrient availability suppresses ULK1 through its phosphorylation (at distinct non-AMPK target sites) by mTORC1 (10, 11). Although ULK1, per se, has been widely studied (12), its direct role in hepatic lipid metabolism and contribution to the MASLD spectrum has remained largely unclear.

Hepatic triglyceride (TG) levels are regulated through multiple mechanisms, including dietary lipid uptake, de novo lipogenesis (DNL), lipid droplet turnover, and secretion of VLDL (13). Increased DNL in obesity contributes to the pathogenesis of MASLD and MASH (14, 15). Feeding and insulin availability drive DNL, which is regulated by transcriptional mechanisms including RORα (16), PPARγ (17), and SREBP-1c (18). NCOA3, a cofactor in the p160 coactivator family (19), is broadly involved in many pathophysiological pathways, including nuclear hormone receptor–driven tumorigenesis, mammary gland development, cell proliferation, and inflammation, in addition to being a key regulator of lipid metabolism (20). Consistently, in mice fed a high-fat diet (HFD), deletion of NCOA3 was shown to improve hepatic steatosis and inflammation, due, in part, to inhibition of the transcriptional activity of nuclear receptors PPARα and LXR (20).

Expression of ULK1 in human MASH livers and in livers of HFD-fed mice revealed a marked reduction in hepatic ULK1 expression relative to their corresponding controls. Thus, we sought to determine how ULK1 regulates hepatic lipid metabolism. Consistently, ULK1 KO livers displayed significantly increased hepatic TG levels and progressed to develop hepatic inflammation and fibrosis in the absence of defects in basal hepatic autophagy. Phospho-proteomics revealed NCOA3 as a ULK1 phospho-target that directly regulates the de novo lipogenic program. Indeed, expression of phosphorylation-deficient NCOA3 mutants, which exhibit unhindered DNA binding, in hepatocytes replicates the phenotype of ULK1 KO livers (i.e., dysregulated induction of lipogenic genes), while pharmacological inhibition or genetic loss of NCOA3 in vivo is sufficient to normalize hepatic TG levels in hepatocyte-specific ULK1 KO mice. Hence, we show that ULK1 restrains the hepatic de novo lipogenic program by phosphorylating and inhibiting NCOA3, providing a framework pointing to the ULK1-NCOA3 axis as a potential therapeutic target against this highly pervasive disease cluster.

## Results

### Human and murine MASLD associate with reduced ULK1 expression.

**Figure 1:**
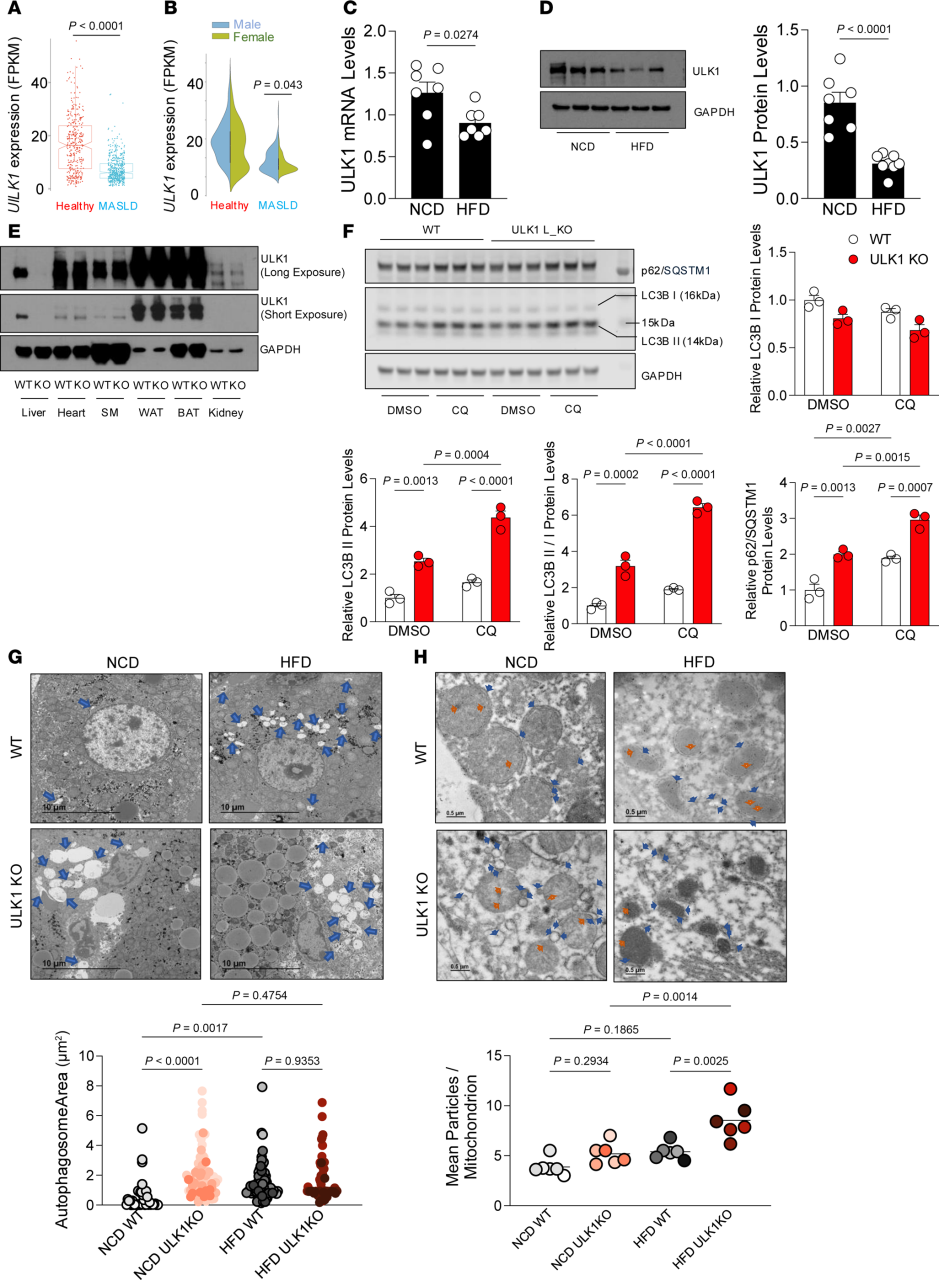
Human and murine MASLD associate with reduced ULK1 expression. (**A**) Box-and-whisker plot of ULK1 transcript abundance (fragments of kilobase per million mapped reads) values) in healthy (*n* = 356) versus MASLD (*n* = 503) human liver samples. (**B**) Bivariate violin plot of ULK1 expression stratified by sex. (**C** and **D**) mRNA (**C**) and protein (**D**) levels of ULK1 measured in livers of mice fed an NCD (*n* = 7) and those fed a 60% HFD (*n* = 7). (**E**) Generation of L-ULK1 KO mice and protein levels of ULK1 in liver and other tissues of WT (*n* = 8) and L-ULK1 KO (*n* = 9) mice. (**F**) Protein levels of markers for autophagosome formation in WT and L-ULK1 KO mice injected with 40 or 50 mg/kg CQ at 0, 6, 18, and 21 hours prior to euthanasia. (**G**) Electron micrographs of liver tissues of WT (*n* = 4) and L-ULK1 KO (*n* = 4) mice in NCD and HFD conditions. Blue arrows represent autophagosomes, which are quantified in bar graphs. (**H**) LC3B immunogold labeling of electron micrographs in liver tissues of WT (*n* = 5) and L-ULK1 KO (*n* = 5) mice in NCD and HFD conditions. Blue arrows indicate immunogold dots co-located with phagophore structures; orange arrows display representative nonphagophore immunogold labeling. All data represent the mean ± SEM. (**A**–**D**) Data were analyzed by 2-tailed Student’s *t* test and actual *P* values are shown. (**F**) Data were analyzed by 2-way ANOVA to assess the effects of genotype and CQ treatment, followed by Tukey’s post-hoc test; actual *P* values are shown. See Supporting Data files for genotype × treatment interaction 2-way ANOVA statistics. BAT, brown adipose tissue; SM, skeletal muscle; WAT, white adipose tissue.

To investigate the relationship between ULK1 expression and hepatic lipotoxicity, we analyzed large-scale human liver transcriptomic datasets and found a significant decline in ULK1 expression in patients with MASLD compared with healthy individuals, as well as in livers from HFD-fed mice. (Figure 1, A–D). Interestingly, when ULK1 expression was compared between men and women in the MASLD cohort, it was markedly reduced in women relative to men. We also examined expression levels of other ATG proteins (e.g., ATG7, ref. 21; ATG5, ref. 22; and ATG13, ref. 23), and we observed that the levels of these proteins were not decreased in the livers of HFD-fed mice (Supplemental Figure 1A; supplemental material available online with this article; https://doi.org/10.1172/JCI191101DS1).

### Loss of ULK1 does not affect basal hepatic autophagy.

To determine the consequence of loss of ULK1 in liver, we generated hepatocyte-specific ULK1 KO mice by crossing mice harboring the floxed ULK1 allele with those expressing the *Cre* recombinase driven by the hepatocyte-specific albumin promoter. As expected, ULK1 levels were depleted specifically in hepatocytes but remained intact in other tissues (i.e., heart, skeletal muscle, white adipose tissue, brown adipose tissue, kidney) (Figure 1E).

Given the regulatory role of ULK1/ATG1 in autophagy (9, 24), we sought to determine if loss of ULK1 impaired autophagic flux, which could potentially alter hepatic lipid metabolism observed during obesity and in the ULK1 KO mice. To this end, we determined autophagy flux in mice fed a normal chow diet (NCD) and HFD-fed mice by injecting them with intraperitoneal chloroquine (CQ) (25) and measuring the turnover rates of the autophagosome marker LC3-II and autophagy cargo p62/SQSTM1 (26). Although ULK1 has been shown to control nutrient-regulated autophagy (11, 27, 28), under basal fed conditions, autophagy flux (namely, p62 and LC3-II turnover) was not repressed in ULK1 KO livers relative to controls, indicated by equivalent turnover rate of LC3-II and p62, with greater overall increases in LC3-II levels after CQ injection (Figure 1F). The number of autophagosomes was increased in NCD- or HFD-fed mice with hepatic deficiency of ULK1 (Figure 1G). Importantly, immunogold labeling of membrane-bound LC3-II in electron micrographs of liver samples of ULK1-deficient mice fed an HFD was increased, further supporting the conclusion that autophagic flux was not decreased (Figure 1H). We also confirmed an increase in the number of autophagosomes and lysosomes in an in vitro system after ULK1 knockdown in Hepa1c1c7 cells (Supplemental Figure 1B). These data indicate loss of ULK1 does not affect basal autophagy in livers.

Autophagic flux was also assessed. After transfection of ULK1 siRNA in Hepa1c1c7 cells, autophagosome-lysosome fusion was inhibited by treatment with CQ. This assay confirmed that autophagy flux (P62 and LC3-II turnover) was not reduced in ULK1 knockdown relative to controls (Supplemental Figure 1C). Taken together, these data indicate ULK1 deficiency in hepatocytes does not inhibit autophagic flux under basal fed states.

### Hepatocyte-specific deletion of ULK1 induces obesity and hepatic steatosis.

**Figure 2:**
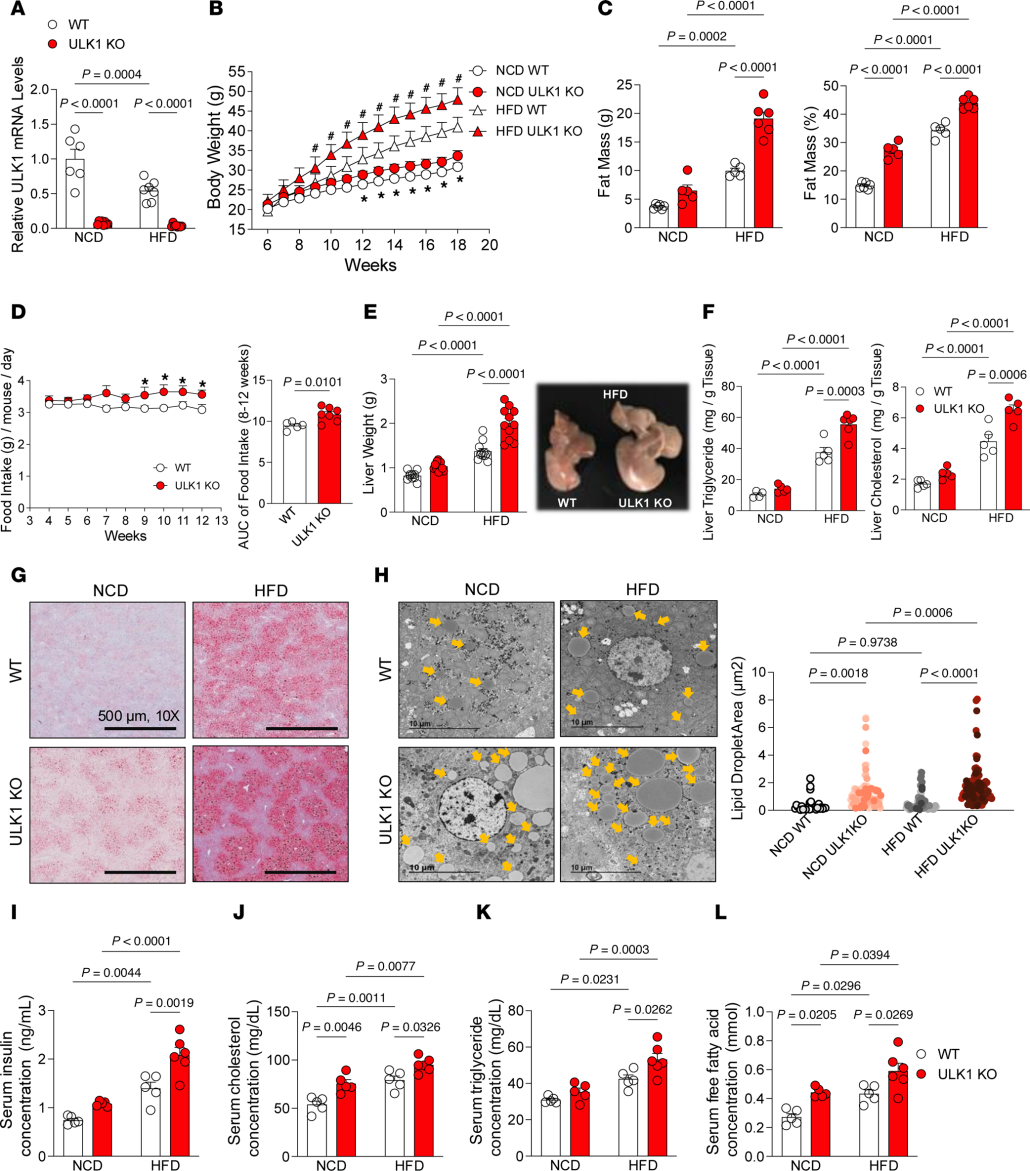
Hepatocyte-specific deficiency of ULK1 induces obesity and metabolic dysfunction–associated steatotic liver disease. (**A**) ULK1 mRNA levels measured in liver tissues from WT (*n* = 7–8) and L-ULK1 KO (*n* = 9) mice in NCD and HFD conditions for 12 weeks. (**B** and **C**) Body weights (**B**) and fat mass (**C**) of 18-week-old WT (*n* = 7–10) and L-ULK1 KO (*n* = 7–10) mice in NCD and HFD conditions. (**D**) Food intake of WT (*n* = 5) and L-ULK1 KO (*n* = 7) mice fed an NCD. (**E**) Liver weight of 18-week-old WT (*n* = 7-10) and L-ULK1 KO (*n* = 7–10) mice in NCD and HFD conditions. (**F**) Levels of liver TG and cholesterol content measured in WT and L-ULK1 KO mouse livers in NCD (*n* = 5) and HFD (*n* = 5) conditions. (**G**) Oil Red O staining of livers isolated from WT (*n* = 4) and L-ULK1 KO (*n* = 4) mice in NCD and HFD conditions. (**H**) Lipid droplet number determined from electron micrographs of liver tissues of WT (*n* = 7) and L-ULK1 KO (*n* = 6) mice under NCD and HFD conditions. (**I–L**) Serum concentrations of insulin (**I**), cholesterol (**J**), TG (**K**), and free fatty acids (**L**) in WT (*n* = 5) and L-ULK1 KO (*n* = 5) mice under NCD and HFD conditions. All data represent the mean ± SEM. Data in **B** and **D** were analyzed by 2-tailed Student’s t test. (**A**, **C**, **E**, **F**, and **H**–**L**) Data were analyzed by 2-way ANOVA to assess the effects of genotype and diet, followed by Tukey’s post hoc test for multiple comparisons; *P* values are shown. See Supporting Data files for genotype × treatment interaction 2-way ANOVA statistics.

To determine the contribution of loss of ULK1 in the development of hepatic steatosis, we subjected control and hepatocyte-specific ULK1 KO mice to 12-weeks of NCD or HFD feeding. As expected, HFD feeding reduced *Ulk1* expression in control livers (Figure 2A). Interestingly, hepatocyte-specific ULK1 KO mice displayed marked obesity as indicated by increases in body weight and fat mass (Figure 2, B and C), with no difference in absolute lean mass when compared with control mice (Supplemental Figure 2A). However, as typically observed in obesity models, HFD-fed ULK1 KO mice had a proportionate decrease in lean mass when normalized to body weight (Supplemental Figure 2A).

To evaluate potential mechanisms for weight gain, food intake was manually measured, and energy expenditure determined in Comprehensive Lab Animal Monitoring System metabolic chambers. We observed a modest increase in food intake in hepatocyte-specific ULK1 KO mice (Figure 2D). There was no reduction in total energy expenditure in ULK1 KO mice relative to control mice (Supplemental Figure 2, B–E). Furthermore, Seahorse respirometry in frozen liver tissue showed no differences in tissue respiration in ULK1 KO mice compared with controls (Supplemental Figure 2F). Strikingly, deficiency of ULK1 led to markedly increased liver weight in HFD-fed mice (Figure 2E) that correlated with increases in liver TG and cholesterol levels (Figure 2F). Consistent with the onset of hepatic steatosis, Oil Red O staining of liver tissues from 20-week-old NCD- and HFD-fed mice showed increased lipid accumulation in ULK1 KO livers relative to controls (Figure 2G). These findings were corroborated using ultrastructural analysis by electron microscopy (Figure 2H). Consistent with the key features of the MASLD spectrum (i.e., insulin resistance and elevated circulating lipid levels), we observed significant increases in levels of circulating insulin, TGs, cholesterol, and free fatty acids in HFD-fed, hepatocyte-specific ULK1 KO mice relative to controls (Figure 2, I–L). Interestingly, significant increases in serum cholesterol and free fatty acid levels were observed even in the NCD-fed, hepatocyte-specific ULK1 KO mice, demonstrating a fundamental role of liver ULK1 in preventing MASLD, insulin resistance, and hyperlipidemia.

### Liver ULK1 KO mice are glucose intolerant and insulin resistant.

Because MASLD and MASH are hepatic manifestations of obesity and the metabolic syndrome (1, 29), and because hepatocyte-specific ULK1 KO mice had increases in body weight, fat mass, and circulating lipids, we investigated whether loss of ULK1 in hepatocytes in vivo leads to insulin resistance, a key factor in the development of MASLD and its progression to MASH or cirrhosis. Loss of liver ULK1 exacerbated glucose intolerance and insulin resistance upon HFD feeding and impaired insulin sensitivity in NCD-fed mice (Supplemental Figure 3, A and B). Consistently, deficiency of ULK1 in the liver attenuated insulin-mediated Ser473 AKT phosphorylation in both NCD- and HFD-fed mice, although HFD feeding led to a greater reduction in Ser473 AKT phosphorylation when compared with NCD-fed mice (Supplemental Figure 3C). Consistent with hepatic insulin resistance, NCD- and HFD-fed, hepatocyte-specific ULK1 KO mice had elevated rates of gluconeogenesis, indicated by abnormal pyruvate tolerance tests (Supplemental Figure 3D) and increased expression of genes encoding for rate-limiting gluconeogenic enzymes G6Pase and PEPCK (Supplemental Figure 3E). Taken together, these results show that ULK1 deficiency in the liver impairs systemic glucose homeostasis, which is a critical factor in the development of metabolic liver disease.

### Deficiency of ULK2 in liver does not induce hepatic steatosis.

Autophagy initiation requires the ULK kinases to functionally engage and phosphorylate components of ATG14L/Beclin1/VPS34/class III PI3K to generate the PI3Ps needed for autophagosome formation (30). ULK1 and ULK2 share a high degree of homology in terms of molecular size and the kinase domain (31). Although ULK1 and ULK2 are thought to have overlapping roles in autophagy initiation, there is growing evidence of functional differences between them. For example, ULK1 and ULK2 play different roles in lipid metabolism, at least in adipocytes (32). ULK1 and ULK2 may also exist in distinct molecular complexes, indicating they could potentially have independent functions or modalities of regulation (33).

To determine if ULK2 plays a role in MASLD pathogenesis, we tested the impact of ULK2 ablation on systemic and liver health, and observed that loss of ULK2, confirmed by qPCR (Supplemental Figure 4A), had no effect on body weight and liver weight (Supplemental Figure 4, B and C) and did not alter glucose tolerance (Supplemental Figure 4D) or insulin sensitivity (Supplemental Figure 4E). There were also no differences in circulating lipids or hepatic enzymes in hepatocyte-specific ULK2 KO mice compared with controls. (Supplemental Figure 4, F–J). Furthermore, combined deletion of ULK1 and ULK2 manifested phenotypes mirrored by the ULK1 KO mice (Supplemental Figure 5, A–K). Hence, we conclude it is loss of ULK1, but not ULK2, that leads to MASLD development.

### Hepatic TG secretion is increased by hepatocyte specific ULK1 deficiency.

To determine the mechanisms for development of steatosis in ULK1 KO livers, we first tested if reduced VLDL secretion contributes to hepatic steatosis. VLDL secretion is determined via a temporal assay that estimates the kinetics of TG release (34). To this end, we intraperitoneally injected control and ULK1 KO mice with Poloxamer 407 (35), a lipoprotein lipase inhibitor, and then collected blood at 1, 6, and 24 hours to determine blood TG levels. Consistently across the time course of this assay, we observed that levels of circulating TGs were markedly elevated in ULK1 KO mice regardless of NCD or HFD feeding (Supplemental Figure 6, A–C), indicating that impaired lipoprotein secretion in ULK1 KO livers does not contribute to steatosis, and it is likely that increased VLDL secretion occurs in parallel with steatosis and could be a compensatory mechanism to counter the onset of MASLD in these mice. In accordance with increased VLDL secretion in ULK1 KO mice, levels of hepatic microsomal TG transfer protein (36), an important mediator of hepatic TG release (37), was induced (Supplemental Figure 6D). These data indicate hepatic steatosis in ULK1 KO livers is independent of defects in hepatic TG secretion.

### ULK1 deficiency increases the expression of lipogenic genes in liver.

**Figure 3:**
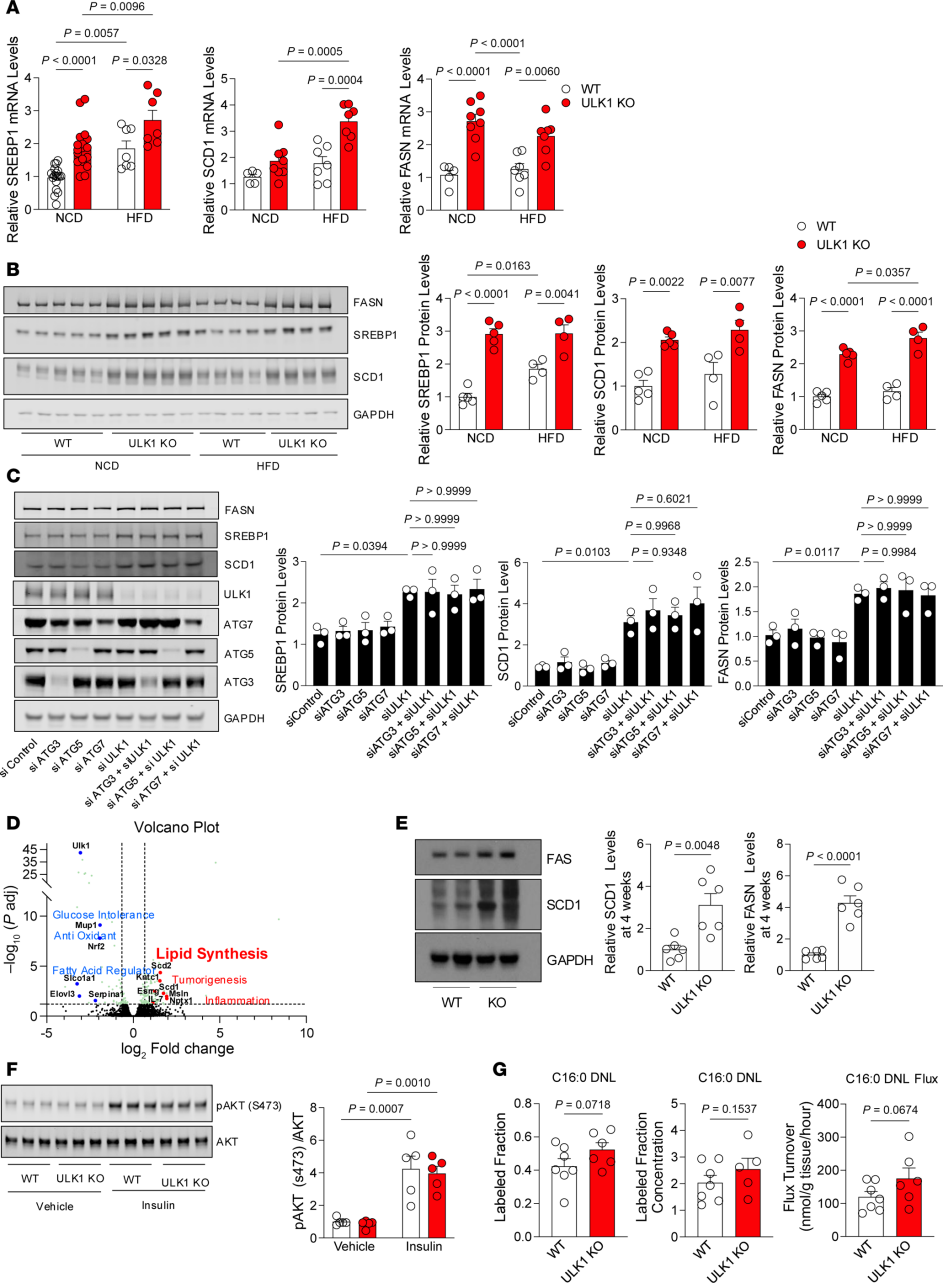
Hepatocyte-specific ULK1 deficiency drives lipogenesis, independent of insulin resistance. Relative (**A**) mRNA and (**B**) protein levels of lipogenic genes (SREBP1c, SCD1, and FAS) in liver tissues of WT (*n* = 4–5) and L-ULK1 KO (*n* = 4–5) mice under NCD and HFD conditions for 12-weeks. (**C**) Protein levels of lipogenesis regulators after silencing of ULK1 or other autophagy-related genes in hepa1c1c7 cells. (**D**) Volcano plot of RNA-Seq performed in 4-week-old WT and L-ULK1KO mice. (**E** and **F**) Protein levels of lipogenic regulators (**E**) and AKT phosphorylation (**F**) in liver tissues of 4-week-old WT (*n* = 5) and L-ULK1 KO (*n* = 5) mice. (**G**) Fractional labeling, concentration, and flux turnover of C16:0 palmitic acid in WT and liver-specific ULK1 KO mice at 4 weeks of age (*n* = 6–8/group). KO livers displayed a trend toward increased labeling and flux turnover. All data represent the mean ± SEM. In **E** and **G**, data were analyzed by 2-tailed Student’s *t* test; in **C**, 1-way ANOVA was used. Data in **A**, **B**, and **F** were analyzed by 2-way ANOVA to assess the effects of genotype and diet, followed by Tukey’s post hoc test for multiple comparisons; actual *P* values are shown. See Supporting Data files for genotype × treatment interaction 2-way ANOVA statistics.

Having excluded changes in TG secretion or mitochondrial dysfunction as potential causes for the development of MASLD, we asked whether loss of ULK1 drives the lipogenic program in liver. Obesity and insulin resistance are well-known drivers of hepatic de novo lipogenesis (14). Accordingly, we examined mRNA expression and protein levels of key lipogenic genes *Srebp1*, *Scd1*, and *Fasn* (38), and noted that expression of these genes and their protein products was significantly induced by the deficiency of *Ulk1* in liver (Figure 3, A and B). We determined if this role of ULK1 in suppression of lipogenesis is independent of canonical autophagy. Indeed, silencing core *Atg* genes, *Atg3*, *Atg5*, or *Atg7*, in Hepa1c1c7 cells via siRNAs did not increase the expression levels of *Srebp1*, *Scd1*, or *Fasn*, when compared with the marked increase in the lipogenic program in cells silenced for *Ulk1* (Figure 3C). Furthermore, co-silencing ULK1 with each of these *Atg* genes led to similar increases in expression of lipogenic genes *Srebp1*, *Scd1*, and *Fasn*, indicating that induction of lipogenesis in siULK1 cells occurs independently of core *Atg* genes.

To comprehensively elucidate how the transcriptional landscape is affected by ULK1 deficiency, bulk RNA-Seq was performed in livers obtained from 4-week-old mice, which exhibited increased expression of candidate lipogenic genes prior to onset of obesity and insulin resistance (Figure 2B, Figure 3F, and Supplemental Figure 7A). Volcano plots revealed global induction of the expression of lipogenic genes (Figure 3D), as exemplified by induction of mRNAs encoding for *Scd1*, *Scd2*, *Pnpla3*, *Pnpla5*, *Acsl3*, *Pdk4*, and *Hmgcr* (Supplemental Figure 7B). Indeed, pathway analysis revealed that lipid synthesis pathways were 3 of the most highly induced pathways, second only to the cell cycle, under ULK1-deficient conditions (Supplemental Figure 7C).

### Increased lipogenic gene expression in ULK1 KO livers is not due to insulin resistance and obesity.

We then examined if the induction of the lipogenic gene expression program in ULK1 KO livers is secondary to insulin resistance and obesity or whether this is a direct effect of loss of ULK1 per se. Strikingly, we observed increased levels of lipogenic proteins in livers of 4-week-old ULK1 KO mice in the fed state (Figure 3E). This early increase in levels of lipogenic proteins FAS and SCD1 in 4-week-old ULK1 KO mice occurred well before the onset of insulin resistance, because insulin-stimulated AKT phosphorylation was found to be equivalent in age-matched control and ULK1 KO livers (Figure 3F). Thus, induction of lipogenic genes is a likely direct effect of ULK1 deficiency and is not a secondary effect of insulin resistance.

### Hepatic ULK1 deletion accelerates de novo lipogenesis.

To determine whether hepatic ULK1 directly regulates DNL in vivo, we performed stable isotope tracing using deuterium oxide (99.9%) in 4-week-old mice with liver-specific ULK1 deletion. At this young age, variability in body weight and food intake is minimal, thereby excluding secondary contributions from dietary overload or obesity. Quantitative analysis of C16:0 palmitic acid revealed a consistent increase in deuterium oxide incorporation in ULK1-deficient livers relative to WT controls. Although the increase in fractional labeling and concentration did not reach statistical significance, flux turnover displayed a borderline elevation (Figure 3G), suggesting a trend toward enhanced lipogenic activity in the absence of ULK1.

We next assessed the total abundance of major DNL-derived fatty acids. Among these, C16:1 palmitoleic acid was significantly elevated in ULK1 KO mice (Supplemental Figure 6E), whereas other fatty acids, including C16:0, C18:0 stearic acid, and C18:1 oleic acid, showed upward trends. Because palmitoleic acid is generated through desaturation of palmitic acid, this increase is consistent with an accelerated flux through the lipogenic pathway. Isotopologue distribution analysis further confirmed these findings. Across all measured fatty acids, KO livers incorporated a greater proportion of labeled carbons, with the most striking enrichment observed in C16:1 palmitoleic acid (Supplemental Figure 6, F–I). These results indicate that loss of hepatic ULK1 enhances the synthesis of both saturated and monounsaturated fatty acids under basal dietary conditions.

Together, these data establish that ULK1 normally acts to restrain hepatic DNL and that its loss is sufficient to increase isotopic flux into lipids even in young, lean animals. Given these effects under basal conditions, we anticipate that the divergence between WT and KO livers would be further amplified under HFD feeding, thereby accelerating the progression toward hepatic steatosis.

### ULK1 phosphorylates proteins with roles in lipid metabolism.

**Figure 4:**
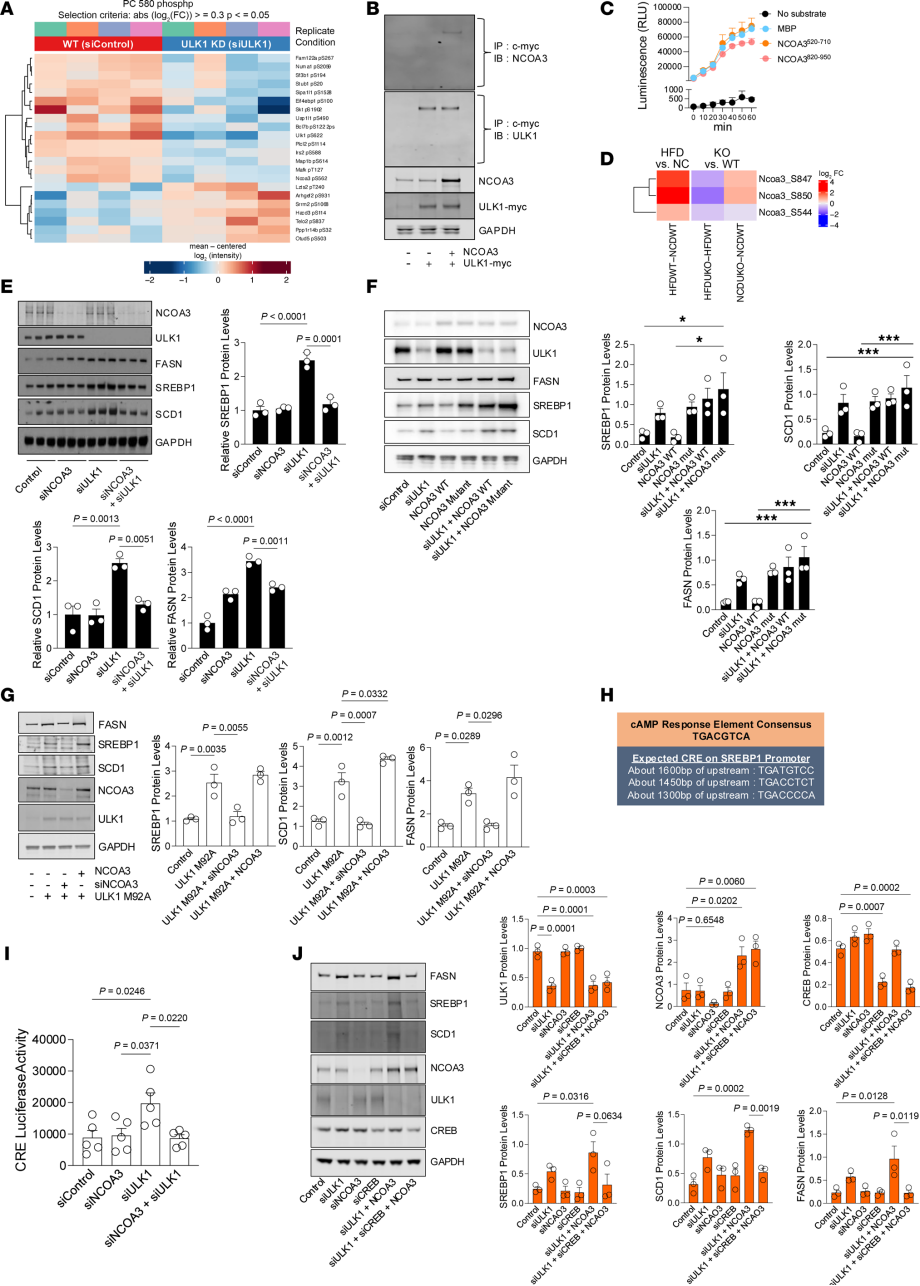
Phosphorylation of NCOA3 by ULK1 blocks lipogenesis by modulating transcriptional activity of the CREB-CBP complex. (**A**) Phosphoproteomic heatmap showing differential phosphorylation events in WT and ULK1 knockdown (KD) cultured hepatocytes. (**B**) Co-immunoprecipitation and immunoblot analyses demonstrating interaction of transfected myc-tagged ULK1 and NCOA3 in hepa1c1c7 cells. (**C**) In vitro kinase assay confirming direct ULK1-dependent phosphorylation of NCOA3. (**D**) Heatmap depicting repression of NCOA3 phosphorylation at S544, S847, and S850 in hepatocyte-specific ULK1 KO mice (UKO) fed NCD or HFD. Color scale represents log2 fold change, with red indicating increased phosphorylation and blue indicating decreased phosphorylation. (**E**) Protein levels of lipogenic regulators after combined silencing using siULK1 (50 nM) and siNCOA3 (50 nM) in hepa1c1c7 cells. (**F**) Hepa1c1c7 cells were transfected with siULK1 (50 nM), NCOA3 (300 ng), and a phosphorylation defective NCOA3 mutation (mut) (300 ng), and then protein levels of lipogenic regulators were measured. (**G**) Lipogenic regulators in hepa1c1c7 cells transfected with NCOA3 (300 ng), siNCOA3 (50 nM), and ULK1 M92A (300 ng). (**H**) CRE consensus sequence and expected CREB binding sites on the promoter region of SREBP1c. (**I**) Luciferase activity in hepa1c1c7 cells transfected with siULK1 (50 nM), siNCOA3 (50 nM), and luc-CRE (200 ng). (**J**) Lipogenic regulators in hepa1c1c7 cells transfected with siULK1 (50 nM), siNCOA3 (50 nM), siCREB (50 nM), and NCOA3 (300 ng). All data represent the mean ± SEM. Data were analyzed by 1-way ANOVA followed by Tukey’s post hoc test versus the control group (**E**–**G**, **I**, and **J**). Actual *P* values are shown; in **F**, *P < 0.05 versus control or NCOA3 WT. FC, fold change; HFDUKO, ULK1 KO mice fed an HFD; HFDWT, WT mice fed an HFD; MBP, myelin basic protein; NCDUKO, ULK1 KO mice fed an NCD; NCDWT, WT mice fed an NCD.

Given that hepatic lipid accumulation in ULK1 KO livers appears to be unrelated to its known roles in autophagy, we tested the hypothesis that ULK1 mediates these effects by phosphorylating other downstream target proteins, which may have direct roles in regulating the lipogenic program. To this purpose, we performed liquid chromatography–tandem mass spectrometry–based phosphoproteomics on lysates of Hepa1c1c7 cells in which ULK1 was silenced, to determine the pool of dephosphorylated proteins in siULK1 cells when compared with controls. This analysis revealed several proteins whose phosphorylation is reduced after ULK1 silencing, including FAM122A, SF3B1, EIF4EBP1, IRS2, MAP1B, MAFK, and NCOA3 (Figure 4A). Interestingly, among the candidate proteins, FAM122A (39), SF3B1 (40), EIF4EBP1 (41), IRS2 (42), and NCOA3 (20) have each been reported to regulate lipid metabolism, suggesting that 1 or more of these downstream phosphoproteins of ULK1 is potentially involved in regulating the lipogenic program.

### ULK1 phosphorylates NCOA3 to repress lipogenesis.

Because phosphorylation of NCOA3 regulates transcription of several target genes by modulating transcription mediated by cAMP response element binding protein (CBP-CREB), and because CREB binds to response elements in the *Srebp-1* gene to stimulate lipogenesis (43), we hypothesized that ULK1 phosphorylates NCOA3 to regulate de novo lipogenesis. Supporting this hypothesis, our co-immunoprecipitation results show that myc-tagged ULK1 interacts with NCOA3 (Figure 4B). An in vitro kinase assay (Supplemental Figure 8) also revealed that ULK1-mediated phosphorylation of both NCOA3 fragments (520–710 and 820–950) was comparable to phosphorylation levels of its known target and positive control, myelin basic protein. Expectedly, the negative control, which does not contain the substrate, yielded minimal luminescence, confirming the absence of signal generated from ULK1 auto-phosphorylation. Furthermore, kinetic luminescence measurements over 60 minutes demonstrated similar reaction profiles for both NCOA3 fragments and myelin basic protein, indicating that NCOA3 is a direct substrate of ULK1. These findings suggest that ULK1-mediated phosphorylation of NCOA3 is direct and involves multiple sites present in the tested fragments (Figure 4C).

We performed phosphor-proteomic analysis on liver tissues from ULK1 KO mice to identify which sites of NCOA3 are phosphorylated by ULK1 in an in vivo model. NCOA3 phosphorylation at three sites (S544, S847 and S850) was markedly suppressed in KO animals (Figure 4D). The similar NCOA3 abundance across groups indicates the decrease in NCOA3 phosphorylation is not due to reduced protein levels (Supplemental Figure 9), thereby supporting that ULK1 directly phosphorylates NCOA3. If increased lipogenesis in cells depleted of ULK1 is due to gain in function of NCOA3, then inhibiting NCOA3 should block the induction of lipogenesis observed in siULK1 cells. To test this possibility, Hepa1c1c7 cells were silenced for NCOA3 or ULK1 or both. Co-silencing of ULK1 and NCOA3 reversed the induction of lipogenic gene expression induced by ULK1 deficiency alone (Figure 4E), whereas depletion of NCOA3 alone had no effect on lipogenic gene expression (Figure 4E).

### ULK1 regulates lipogenesis by phosphorylating NCOA3 and modulating the NCOA3-CBP-CREB complex.

To evaluate the physiological relevance of NCOA3 phosphorylation by ULK1 in the regulation of hepatic lipogenesis, we conducted a search of the predicted phosphorylation sites within the NCOA3 protein, on the basis of its consensus sequences and of published literature (44, 45). We identified 14 serine phosphorylation sites within NCOA3 protein (as depicted in Supplemental Figure 10A). Accordingly, we created a NCOA3-mutant DNA construct by replacing all 14 predicted serine phosphorylation sites with alanine. Hepa1c1c7 cells were then transfected with ULK1 siRNA and WT or phospho-mutant NCOA3, after which we determined the functional interaction between ULK1 kinase and its substrate NCOA3 in the regulation of the de novo lipogenic program. Consistent with our hypothesis, expression of WT NCOA3 in cells with intact ULK1 did not induce the de novo lipogenic program, because active endogenous ULK1 likely restrains NCOA3 transcriptional activity through its phosphorylation. By contrast, overexpression of the phosphorylation-resistant NCOA3 mutant markedly induced levels of SREBP1, which was paralleled by significant increases in FASN and SCD1. However, with expression of WT or mutant NCOA3 in conjunction with ULK1 knockdown, we observed a synergistic induction of the lipogenic program, indicated by marked increases in levels of all 3 lipogenic proteins: SREBP1, FASN, and SCD1 (Figure 4F). Given this observation, we analyzed the effect of single NCOA3 mutants to determine which of these 14 candidate phosphorylation sites play a role in repressing de novo lipogenesis. The examination of the effect of expression of each phosphor-mutant on protein levels of SREBP1, SCD1 and FASN indicated there are differences in the pattern of regulation of expression of lipogenic genes (indicated by corresponding protein levels) as a function of the NCOA3 phosphorylation site that is mutated (Supplemental Figure 10B). Together, these findings suggest that NCOA3, as a downstream target of ULK1, plays a critical role in hepatic lipid metabolism, and that ULK1 KO affects this process by modulating its phosphorylation.

We then asked if ULK 1 kinase activity is required to repress lipogenic gene expression. To test this possibility, we generated the kinase-dead ULK1 M92A mutant (46). Expressing this M92A kinase-dead ULK1 mutant in Hepa1c1c7 cells markedly induced lipogenic gene expression, which phenocopied the induction of lipogenesis seen with ULK1 deficiency. To rigorously test the role of the ULK1-NCOA3 axis in blocking lipogenesis, we determined if silencing NCOA3 blocked the increase in lipogenic gene expression induced by overexpression of the kinase-dead ULK1 M92A mutant (Figure 4G). Indeed, depletion of NCOA3 normalized the induction of lipogenesis in ULK1 kinase-dead M92A mutants. Taken together, these data indicate ULK1 kinase activity represses the lipogenic gene expression program via a mechanism that likely relies on NCOA3 phosphorylation.

### ULK1 regulates NCOA3 transcriptional activity.

An important transcriptional target of NCOA3 that may potentially regulate the expression of lipogenic genes is the CREB-binding protein CBP. Given the observed changes to SREBP1, the master transcriptional regulator of lipogenic genes, including *Scd1* and *Fasn* (38, 47), in livers silenced for *Ulk1* (Figure 3B) or cells expressing the NCOA3 mutants (Figure 4F and Supplemental Figure 10B), we focused on SREBP1. Furthermore, the *Srebp1* gene possesses CREB binding response elements (CREs), which facilitate transcriptional regulation by the CREB-CBP complex (48, 49). Our analyses of *Srebp1* revealed 3 candidate CREs at –1,300, –1,450, and –1,600 bp in the promoter region of *Srebp1* (Figure 4H). Consequently, we constructed a CRE consensus luciferase reporter and determined its activity in cells in which NCOA3 and ULK1 were individually or simultaneously silenced. Confirming our hypothesis, silencing ULK1 in Hepa1c1c7 cells increased CRE luciferase activity by 2-fold, whereas silencing NCOA3 alone did not alter basal luciferase activity. In clear contrast, silencing NCOA3 prevented the induction of luciferase activity in response to ULK1 depletion (Figure 4I). Consistently, epistasis experiments revealed that expression of NCOA3 in siULK1 Hepa1c1c7 cells led to markedly increased protein levels of SREBP1, SCD1, and FASN, whereas depleting CREB under these conditions abrogated the synergistic effect of ULK1 silencing and NCOA3 overexpression on the induction of SREBP1, SCD1, and FASN (Figure 4J). Taken together, these data indicate the interaction of CREB and NCOA3 is required for transactivating lipogenic gene expression under conditions when ULK1 levels are depleted, such as those observed in livers of obese mice and human patients with MASH.

### ULK1-NCOA3 double-KO livers do not manifest MASLD or insulin resistance.

**Figure 5:**
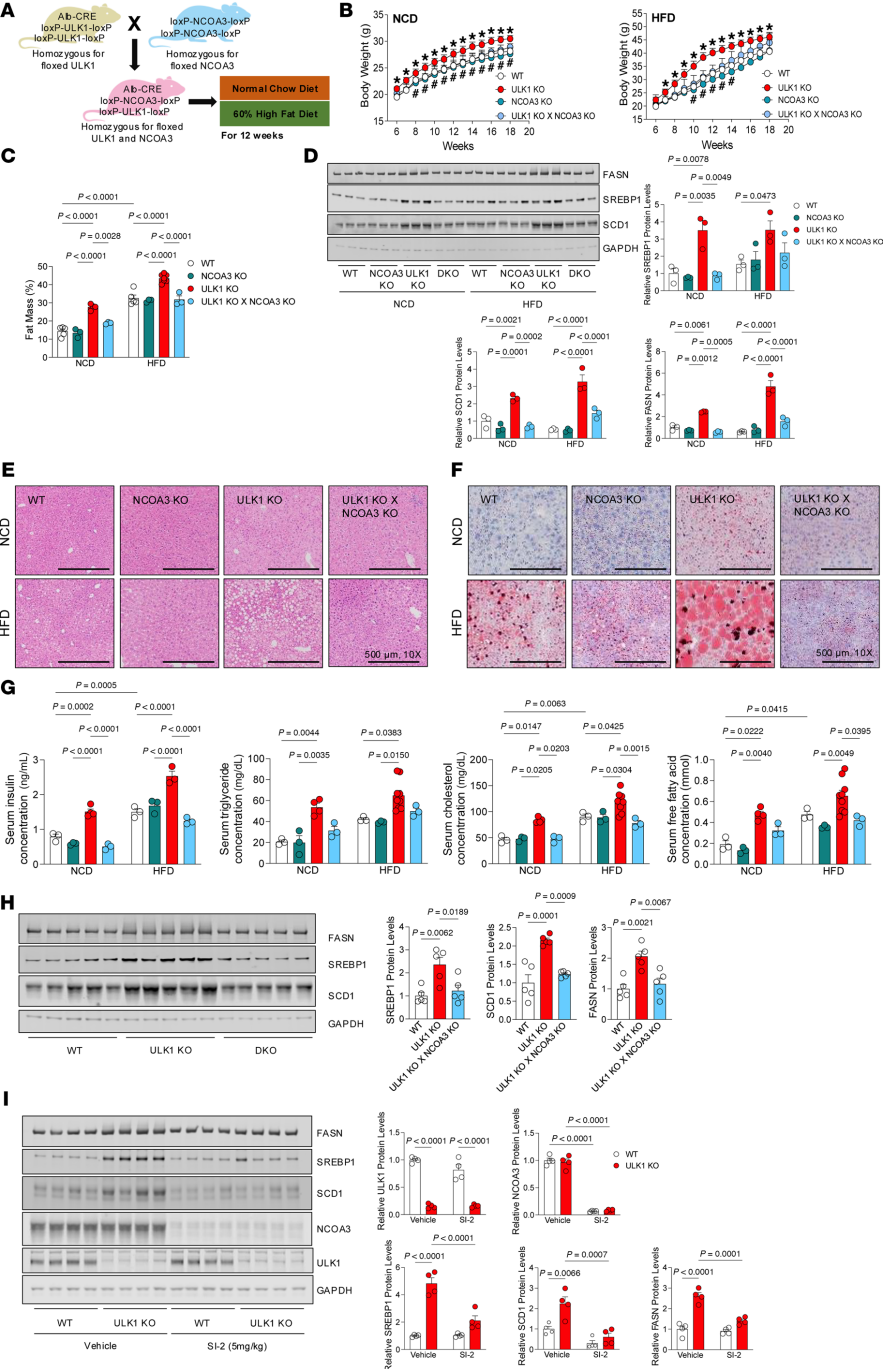
Hepatocyte-specific loss of NCOA3 prevents MASLD due to ULK1 deficiency. (**A**) Generation of hepatocyte specific DKO of ULK1 and NCOA3 (ULK1KO × NCOA3KO) mice. (**B**) Weekly body weight in WT (*n* = 6 or 4), L-NCOA3KO (*n* = 7), L-ULK1KO (*n* = 7 or 8), and L-ULK1KO × NCOA3 KO (*n* = 7) under NCD and HFD feeding for 12 weeks. (**C**) Fat mass in WT (*n* = 6), L-NCOA3KO (*n* = 7), L-ULK1KO (*n* = 7 or 8), and L-ULK1KO × NCOA3 KO (*n* = 7) after 12 weeks of NCD and HFD feeding. (**D**) Protein levels of lipogenic regulators (FASN, SREBP1, and SCD1) in WT (*n* = 3), L-NCOA3KO (*n* = 3), L-ULK1KO (*n* = 3), and L-ULK1KO × NCOA3KO (*n* = 3) after 12 weeks of NCD and HFD feeding. (**E** and **F**) H&E (**E**) and Oil Red-O staining (**F**) in livers obtained from WT (*n* = 4), L-NCOA3KO (*n* = 4), L-ULK1KO (*n* = 4), and DKO (*n* = 4) mice after 12 weeks of NCD and HFD feeding. (**G**) Serum concentrations of insulin, TG, cholesterol, and free fatty acids in WT (*n* = 3), L-NCOA3KO (*n* = 3), L-ULK1KO (*n* = 4 or 8), and L-ULK1KO × NCOA3KO (*n* = 3) mice after 12 weeks of NCD and HFD feeding. (**H**) Protein levels of lipogenic regulators and enzymes in liver tissues from 4-week-old WT, L-ULK1KO, and DKO mice. (**I**) We intraperitoneally injected 18-week-old WT and L-ULK1 KO mice fed an NCD with PBS and SI-2 (5 mg/kg) twice a day for 10 days and protein levels of lipogenic regulators and enzymes were examined. All data represent the mean ± SEM. Data in **B** were analyzed by 2-tailed Student’s *t* test. *P < 0.05 ULK1 KO versus WT; #*P <* 0.05 ULK1 KO × NCOA3 KO versus ULK1 KO. One-way ANOVA was used for **H**, and 2-way ANOVA for **C**, **D**, **G**, and **I** to assess the effects of genotype and diet or treatment, followed by Tukey’s post hoc test. Actual *P* values are shown. See Supporting Data files for genotype × treatment interaction 2-way ANOVA statistics.

To independently validate that ULK1 curtails hepatic lipogenesis by inhibiting NCOA3 function, we generated hepatocyte-specific ULK1-NCOA3 double-KO (DKO) mice, per the strategy depicted in Figure 5A. Interestingly, the obesity phenotype (increased body weight and fat mass) of hepatocyte-specific ULK1 KO mice was reversed when NCOA3 was simultaneously knocked out in liver (Figure 5, B and C). Consistently, hyperglycemia, insulin resistance, and the exaggerated induction of lipogenic gene expression due to ULK1 deficiency were all prevented in hepatocyte-specific ULK1-NCOA3 DKO mice (Supplemental Figure 11 and Figure 5D). Furthermore, the histological and ultrastructural changes induced by ULK1 deficiency, such as lipid droplet accumulation and Oil Red-O positivity, were both prevented after NCOA3 knockdown (Figure 5, E and F). Circulating metabolites reflecting dyslipidemia and insulin resistance (i.e., elevated insulin, TG, cholesterol, and free fatty acid levels) were each found to be normalized with NCOA3 knockdown (Figure 5G).

To determine whether ULK1 directly regulates hepatic lipogenic gene expression in young mice and whether this effect requires NCOA3, we analyzed livers from 4-week-old mice. Immunoblot analysis revealed that the protein levels of SREBP1, SCD1, and FASN were significantly increased in ULK1 KO mice compared with WT controls. Importantly, this induction was abolished in ULK1/NCOA3 DKO mice, in which protein levels remained comparable to those in WT animals (Figure 5H). These findings demonstrate that ULK1 promotes the induction of hepatic lipogenic genes in an NCOA3-dependent manner.

### Pharmacological inhibition of NCOA3 normalizes lipogenesis in ULK1 KO livers.

SI-2 is a small-molecule inhibitor of NCOA3 that leads to NCOA3 degradation (50). We tested whether SI-2 could dampen the induction of lipogenesis in ULK1 KO livers. To this end, control and ULK1 KO mice were administered SI-2 (5 mg/kg) via intraperitoneal injection twice a day for 10 days, following which the expression of hepatic lipogenic genes was determined. Strikingly, SI-2 treatment markedly reduced NCOA3 protein levels and normalized lipogenic gene expression, induced by KO of ULK1 (Figure 5I). Similar observations were noted in Hepa1c1c7 cells treated for 24 hours with 200 nM or 500 nM SI-2, which reduced NCOA3 protein levels and concurrently blunted lipogenic gene expression in ULK1-deficient cells (Supplemental Figure 12).

### The regulation of NCOA3 by ULK1 determines hepatic inflammation and fibrosis.

**Figure 6:**
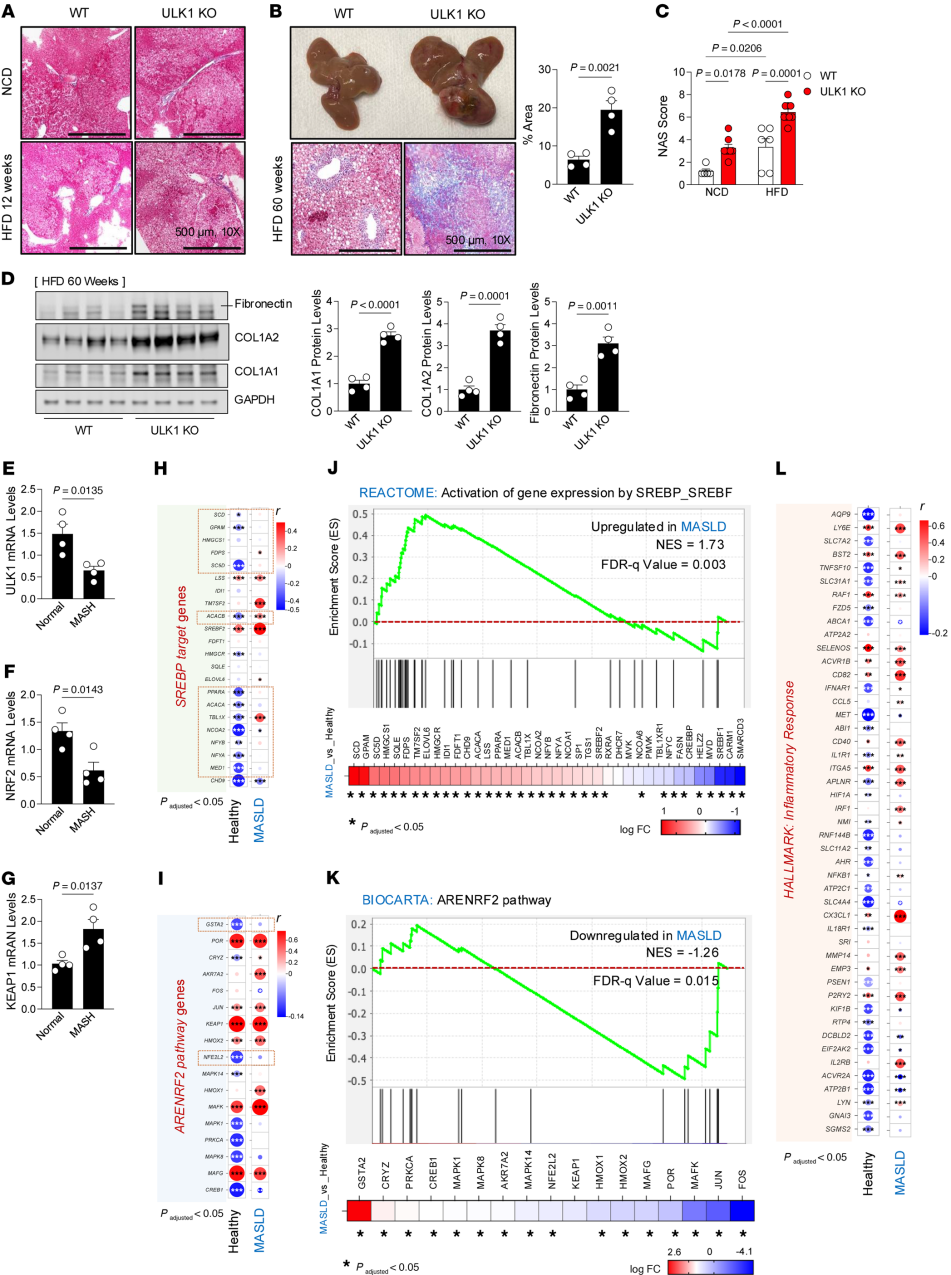
Fibrosis in ULK1-deficient murine livers and changes in lipogenic, inflammatory, and NRF2 target genes in human MASLD livers. (**A**) Representative trichrome staining of liver sections from WT and ULK1 KO mice fed an NCD or HFD for 12 weeks. (**B**) Gross appearance, trichrome staining, and quantification of fibrotic area in livers of WT (*n* = 5) and L-ULK1 KO (*n* = 5) mice after 60 weeks of HFD feeding. (**C**) NAFLD activity score (NAS) in ULK1 KO and WT mice after 60 weeks of NCD and HFD feeding. (**D**) Immunoblot analysis of fibronectin, COL1A2, and COL1A1 in WT and ULK1 KO mouse livers after 60 weeks of HFD feeding. (**E**–**G**) Relative hepatic mRNA levels of ULK1 (**E**), NRF2 (**F**), and KEAP1 (**G**) in normal versus MASLD human samples. (**H**–**L**) Analysis of human liver RNA-Seq datasets, (accession nos. GSE48452 and GSR126848) in 356 control individuals and 503 patients with MASLD. (**H**) Correlational analysis of ULK1 expression and SREBP targets with adjusted *P <* 0.05, in humans with or without MASLD. (**I**) Correlational analysis of ULK1 expression and the ARENRF2 pathway with adjusted *P <* 0.05, in humans with or without MASLD. (**J**) Gene Set Enrichment Analysis illustrating activation of SREBP/SREBF-driven lipogenic transcriptional programs (Reactome) (NES = 1.73; FDR *q* = 0.003). (**K**) GSEA illustrating suppression of the ARENRF2 antioxidant defense pathway (Biocarta) (NES = –1.26; FDR *q* = 0.015). (**L**) Correlation analysis of ULK1 expression and inflammatory response genes with adjusted *P <* 0.05 in humans with or without MASLD. Data are mean ± SEM. Data analyzed by 2-tailed Student’s *t* test in **B** and **D**–**G** and by 2-way ANOVA for **C** to assess the effects of genotype and diet, followed by Tukey’s post hoc test. Actual *P* values are shown. Genotype × treatment interaction 2-way ANOVA for NAS scores are in the Supporting Data file. Correlation coefficients (Pearson’s *r*) represent sample-wise correlations between ULK1 expression and respective target genes. *P < 0.05 vs. healthy.

Although it is well known that HFD feeding per se does not lead to hepatic fibrosis, we examined if loss of ULK1 leads to the onset of fibrosis as a function of diet and duration of feeding. Expectedly, no fibrosis was evident after 12 weeks of HFD feeding (Figure 6A); however, extensive fibrosis was observed in ULK1 KO livers at 60 weeks of HFD feeding, as indicated by increased trichrome staining (Figure 6B). The NAFLD Activity Score analysis revealed significantly higher scores in ULK1 KO mice relative to WT mice under both NCD and HFD conditions. HFD-fed KO mice had the highest scores, indicating more severe hepatic steatosis and inflammation (Figure 6C). Fibronectin, COL1A2, and COL1A1, established markers of fibrosis (51), were increased by ULK1 KO in mice who had been fed a long-term HFD, indicating that ULK1 may play a protective role in mitigating fibrosis under these conditions (Figure 6D).

The transition from steatosis to fibrosis is dependent on, among other factors, increases in oxidative stress–induced hepatocyte injury, inflammation, and stellate cell activation (52). Interestingly, our RNA-Seq data revealed that *Nrf2* expression is repressed in ULK1 KO livers (Figure 3D). NRF2 is a key driver of the cellular antioxidant response (53). Given the role of ROS in the development and progression of steatohepatitis (54), reduced NRF2 could contribute to the transition from steatosis to hepatitis. *Nrf2* induction prevents cellular damage and maintains normal liver function (55) by providing a coordinated cellular defense against oxygen radicals and oxidative stress (56), including antioxidant gene expression.

In addition to changes in gene expression, NRF2 is also regulated by KEAP1, a regulatory protein that binds to NRF2 and targets it for degradation. Under conditions of oxidative stress, KEAP1 releases NRF2, allowing it to drive the antioxidant program (56). In addition to reduced *Nrf2* expression in ULK1 KO livers (Figure 3D), we also analyzed the mRNA expression of *Ulk1*, *Nrf2*, and *Keap1* in liver tissue from patients with MASH and from normal individuals. As anticipated, the *Ulk1* mRNA was decreased in patients with MASH. This occurred in concert with repression of *Nrf2* and induction of *Keap1* mRNA. (Figure 6, E–G). Together these changes suggest an impairment in antioxidant defense mechanisms.

Analysis of large-scale human liver transcriptomic datasets was performed to determine if these patterns could be replicated in a larger population cohort. Correlation analysis revealed negative correlations between *Ulk1* expression and *Srebp1* targets in normal control individuals, which were reversed in patients with MASLD (Figure 6H). Correlation analysis revealed inverse correlations between Ulk1 and *Keap1* and *Nfe2l3*, which suggests they are regulated in opposite directions by ULK1 activity (Figure 6I). Gene Set Enrichment Analysis revealed that MASLD livers exhibited a robust enrichment of SREBP/SREBF-driven de novo lipogenic gene programs (Reactome) (normalized enrichment score [NES] = 1.73; FDR *q* = 0.003) (Figure 6J), consistent with excessive lipid biosynthesis, whereas the ARENRF2-mediated antioxidative response pathway (Biocarta) (NES = –1.26; FDR *q* = 0.015) was significantly suppressed, reflecting impaired redox homeostasis (Figure 6K). These data suggest ULK1 expression could be a nodal regulator linking lipid accumulation to defective antioxidant defense. Together, these findings identify ULK1 repression as an important molecular feature of MASLD that could contribute to metabolic overload through unchecked de novo lipogenesis while increasing vulnerability to oxidative stress, through impaired ARENRF2 signaling, thereby providing a mechanistic framework for how impaired ULK1 activity coordinately exacerbates disease progression independent of its canonical autophagy-related functions.

### NCOA3 inhibition restores antioxidant defense and prevents inflammation in ULK1-deficient livers.

**Figure 7:**
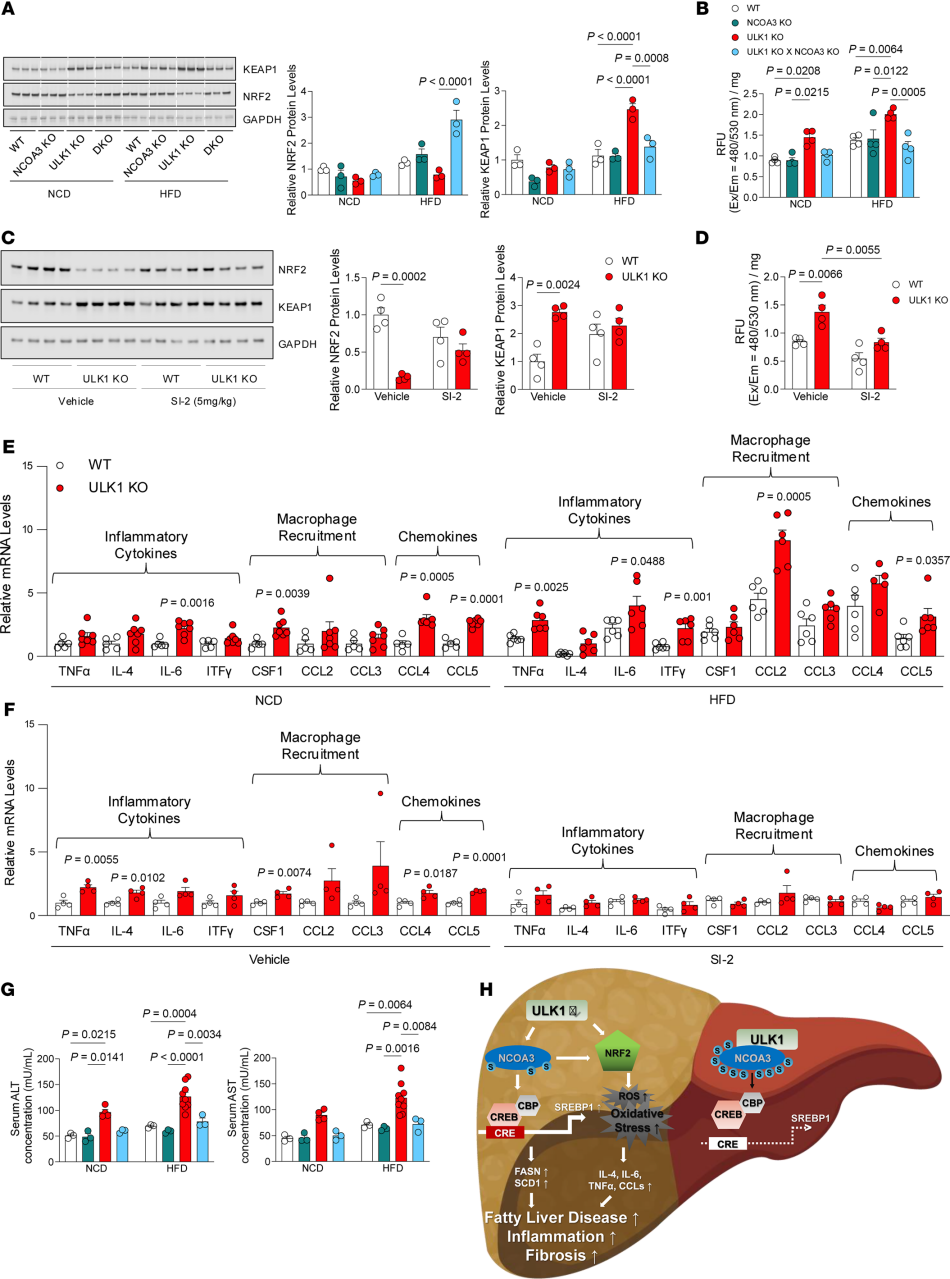
UKL1 deficiency represses NRF2 signaling and promotes oxidative stress and hepatic inflammation through NCOA3-dependent mechanisms. (**A**) Protein levels of NRF2 and Keap1 were examined in WT (*n* = 3), L-NCOA3KO (*n* = 3), L-ULK1KO (*n* = 3), and L-ULK1 KO × NCOA3 KO (DKO) (*n* = 3) after 12-weeks of NCD and HFD feeding. (**B**) ROS levels were determined in WT (*n* = 4), L-NCOA3KO (*n* = 4), L-ULK1KO (*n* = 4), and L-ULK1KO × NCOA3KO (*n* = 4) after 12-weeks of NCD and HFD feeding. (**C**) We intraperitoneally injected 18-week-old WT and L-ULK1 KO mice fed an NCD with PBS and SI-2 (5 mg/kg) twice a day for 10 days and protein levels of NRF2 and Keap1 were examined. (**D**) ROS levels were determined in the same mice described in (**C**). (**E**) mRNA levels of immune response–related genes in liver tissues of 18-week-old WT (*n* = 5–6) and L-ULK1 KO (*n* = 6–7) mice under NCD and HFD conditions, as in **A**. (**F**) qPCR was performed to measure RNA levels of immune response–related genes in liver tissues of 18-week-old NCD-fed WT (*n* = 4) and L-ULK1 KO (*n* = 4) mice after intraperitoneal injection with PBS and SI-2 (5 mg/kg) twice a day for 10 days. (**G**) Serum concentrations of alanine aminotransferase (ALT) and aspartate aminotransferase (AST) in WT (*n* = 3), L-NCOA3 KO (*n* = 3), L-ULK1 KO (*n* = 4), and L-ULK1KO × NCOA3KO (*n* = 3) mice under NCD and HFD conditions. (**H**) Schematic summarizing mechanisms linking ULK1 deficiency with the pathophysiology of hepatic steatosis and progression to MASH and fibrosis. All data represent the mean ± SEM. In **A**–**G**, data were analyzed by 2-way ANOVA to assess the effects of genotype and diet or treatment, followed by Tukey’s post hoc test. Actual *P* values are shown. See Supporting Data files for genotype × treatment interaction 2-way ANOVA statistics.

We also observed an increase in the expression of KEAP1 protein, suggesting that NRF2 function is disrupted in ULK1 KO livers, reflecting an impairment in the overall hepatocellular antioxidant capacity (Figure 7A). Importantly, these changes in NRF2 and KEAP1 protein levels were reversed in ULK1 and NCOA3 DKO mice (Figure 7A). Consistent with dysregulation of NRF2/KEAP1 control of the antioxidant response, (57), we observed elevated ROS levels in ULK1-deficient livers, which were normalized by the loss of NCOA3 (Figure 7B). Hepatocellular injury and altered immune response are central to the pathophysiology of the MASLD spectrum. This is supported by correlation analysis revealing negative correlations between *Ulk1* expression and inflammatory response genes in normal control individuals, which were reversed in patients with MASLD (Figure 6L). Thus, we explored whether pharmacological inhibition of NCOA3 is sufficient to reduce the onset of inflammation in ULK1 KO livers of NCD-fed mice. Indeed, intraperitoneal injection of SI-2, a pharmacological inhibitor of NCOA3, to control and hepatocyte-specific ULK1 KO (L-ULK1 KO) mice fed an NCD, normalized the disrupted NRF2-KEAP1 signaling cascade caused by ULK1 deficiency (Figure 7C). Additionally, the elevated ROS levels resulting from *Ulk1* loss were restored to normal by NCOA3 inhibition (Figure 7D).

We examined the expression of inflammatory and profibrotic genes in control and ULK1 KO livers of mice fed an NCD and those fed an HFD, and we observed that expression of inflammatory and profibrotic genes were globally elevated in KO mice irrespective of diet, and HFD feeding of ULK1 KO mice further augmented inflammation pathway gene expression relative to NCD-fed KO mice (Figure 7E). Treatment of NCD-fed ULK1 KO mice with the NCOA3 inhibitor SI-2 normalized the expression of these transcripts (Figure 7F), suggesting that ULK1 phosphorylation of NCOA3 not only suppresses MASLD but also prevents the onset of inflammation and the progression to MASH or fibrosis in the context of ULK1 deficiency. Consistent with increased fibrosis at 60 weeks of HFD feeding in hepatocyte-specific ULK1 KO livers, we observed that hepatocellular injury reflected by increases in the circulating levels of transaminases alanine aminotransferase and aspartate aminotransferase as early as by 12 weeks of HFD feeding were normalized in ULK1 KO livers co-deleted for NCOA3 (ULK1 KO × NCOA3 KO) (Figure 7G). Collectively, these findings demonstrate that NCOA3 is required for the disruption of NRF2-KEAP1 signaling, oxidative stress, and inflammatory progression in ULK1-deficient livers, and that genetic or pharmacological inhibition of NCOA3 restores antioxidant defense and protects against hepatocellular injury (Figure 7H).

## Discussion

Here we show that expression of hepatic *Ulk1* is significantly reduced in patients with MASLD and in HFD-fed mice. Under lipotoxic conditions, ULK1 activates the p62/KEAP1/NRF2 pathway to enhance antioxidant defenses. Prolonged lipotoxic stress leads to a relative reduction in ULK1 protein levels, potentially due to its sustained engagement in this pathway; however, the precise mechanisms underlying this decrease remain unclear (58). To elucidate mechanisms by which reduced *Ulk1* expression contributes to the pathophysiology of MASLD, we generated hepatocyte-specific ULK1 KO mice. We show that ULK1 loss increases hepatic lipogenesis in parallel with increased insulin resistance, liver inflammation, and pathways promoting hepatic fibrosis. However, the increase in lipogenic capacity precedes the onset of insulin resistance in these mice, indicating that ULK1 deficiency primes hepatocytes for an amplified steatotic and inflammatory response in the presence of caloric overload.

Given the canonical role of ULK1 in the regulation of autophagy, we carefully evaluated autophagic flux in vivo and in cultured hepatocytes after reduction of ULK1 levels, by determining LC3II and SQSTM1 levels after CQ treatment and directly quantifying autophagosome number in electron micrographs under basal and HFD conditions. SQSTM1 protein was modestly elevated in ULK1-deficient cells at baseline. While this increase could be interpreted as representing impaired autophagy, it is not clear whether the elevated SQSTM1 levels exclusively reflect autophagic flux or other mechanisms of SQSTM1 turnover independent of autophagosomes. To directly assess flux, we performed lysosomal inhibition experiments, the gold standard for monitoring autophagic activity (59). Upon CQ treatment, SQSTM1 accumulated in both control and ULK1-deficient cells, confirming effective inhibition of lysosomal degradation. Notably, the greater increase in SQSTM1 after CQ treatment in ULK1-deficient cells indicates basal autophagy flux was not diminished. Consistent with this, the increased number of autophagosomes in these cells is unlikely to reflect a block in degradation, given the robust accumulation of LC3II and SQSTM1 after CQ treatment. Together, these findings demonstrate that both basal and HFD-induced autophagic degradation remain intact in ULK1-deficient cells. Thus, our studies confirm that in the context of ULK1 depletion, the observed dysregulation of the lipogenic program is not attributable to impaired autophagy but is mediated by an autophagy-independent function of ULK1.

Liver-specific ULK1 deletion enhances hepatic de novo lipogenesis in young mice, as shown by increased isotopic labeling and elevated palmitoleic acid in this study, independent of dietary intake or body weight. These findings identify ULK1 as a key suppressor of intrinsic lipogenic flux, loss of which predisposes the liver to steatosis and further exacerbates lipid accumulation under HFD conditions. To identify potential mechanisms, a phosphoproteomic screen was performed and revealed the transcriptional coactivator NCOA3 as a direct target of ULK. We show that ULK1 directly phosphorylates NCOA3 to restrain hepatic de novo lipogenesis. NCOA3 is a global regulator of gene expression, including those regulating lipid metabolism, and its transcriptional activity is regulated by a variety of post-translational modifications, including phosphorylation, ubiquitination, SUMOylation, acetylation, and methylation (60–63). In this study, we observed that depletion of ULK1 in the liver reduced the phosphorylation of NCOA3, thereby identifying what we believe is a novel mechanism that regulates this transcriptional coactivator.

Inactivating putative ULK1 phosphorylation sites on NCOA3 was sufficient to activate the lipogenic program in cultured hepatocytes to the same extent as ULK1 silencing, supporting our proposed concept that ULK1 keeps hepatic de novo lipogenesis in check by phosphorylating and inactivating the transcriptional coactivator NCOA3. Furthermore, the expression of kinase-inactive ULK1 recapitulated the effect of ULK1 deficiency on the hepatocyte lipogenic program. Hence, we present a conceptual framework based on a direct relationship between ULK1 and its phosphorylation of NCOA3, whereby phosphorylated NCOA3 is unable to support the expression of lipogenic targets. By contrast, when ULK1 levels are depleted, dephosphorylated NCOA3 regulates the transcription of several target genes by amplifying CBP-CREB–mediated transcriptional activation (64). Through epistasis studies, we demonstrated that ULK1 modulates the amplification of CBP-CREB by NCOA3 in cultured hepatocytes and that dephosphorylated NCOA3 effectively interacts with CBP-CREB to promote the transcription of SREBP1. NCOR1 is a key transcriptional corepressor with broad roles in downstream signaling pathways. Consistent with prior reports implicating NCOR1 as a direct substrate of ULK1 (65), we observed a substantial decrease in NCOR1 phosphorylation in ULK1-deficient livers (data not shown). Given the central role of NCOR1 in coordinating transcriptional responses, its regulation by ULK1 may represent an additional mechanism by which ULK1 could modulate hepatic metabolic programs. Future studies will focus on characterizing the phosphorylation status and functional consequences of NCOR1 in the context of ULK1 deficiency.

Our study has implications for the development of novel therapeutic targets for MASLD and MASH, raising the possibility that gain-of-function or small-molecule activation of ULK1 could protect against hepatic steatosis. Overexpression of ULK1 is likely to have pleiotropic effects by affecting the roles of AMPK or mTOR in canonical autophagy, depending on the cellular physiological context (i.e., fasting state versus fed state). Indeed, ULK1 independently affects mTORC1 activity as well as cell proliferation (66) or promotes apoptosis (67). Despite the potential pleiotropic effects of directly manipulating ULK1, studies have examined ULK1 as a therapeutic target (68), and because small-molecule activators of ULK1 have been described (69), future studies will be needed to determine if pharmacological ULK1 activation can prevent hepatic steatosis. Interestingly, because, in this conceptual framework, ULK1 inhibits NCOA3, we tested if pharmacological inhibition of NCOA3 using SI-2 can prevent the onset of MASLD. Intriguingly, the administration of SI-2 attenuated the induction of lipogenic enzymes and the markers of hepatic inflammation and fibrosis.

ULK1 deficiency is associated with the progression to steatohepatitis, as evidenced by increases in the levels of liver transaminases and expression of proinflammatory genes in ULK1-deficient mice. Oxidative stress is believed to be an important mediator of hepatic injury and progression to fibrosis in MASLD. Our study identified a role of dysregulation of the ULK1-NCOA3 axis in limiting the antioxidant defense mechanisms in the liver. ULK1 deficiency is associated with reduced expression of *Nrf2* and increased levels of KEAP1, which sequesters and inactivates KEAP1. Inhibition of NCOA3 with SI-2 restored NRF2 and reduced the levels of its inhibitor KEAP1, which would be predicted to increase the activity of antioxidant response pathways. Indeed, the observed increase in tissue oxidant levels in ULK1-deficient livers was normalized when NCOA3 was inhibited pharmacologically or genetically. Together these findings reveal that in addition to regulating lipogenesis, activating the ULK1-NCOA3 axis also lowers oxidative stress and inflammation, which prevents the progression from MASLD to MASH. Thus, the ULK1-NCOA3 axis could serve as a viable therapeutic target not only for preventing hepatic steatosis but also for preventing the progression to fibrosis.

In addition to NCOA3, ULK1 depletion led to decreased phosphorylation of several other proteins in the liver, including FAM122A, SF3B, EIF4BP1, MAFK, and IRS2, which could contribute to the regulation of hepatic lipid metabolism by ULK1; however, our mechanistic studies show a direct role of ULK1 in NCOA3 phosphorylation in repressing lipogenic gene expression. IRS-2 S588 phosphorylation was identified in our phospho-proteomics analysis, and this phosphorylation site remains poorly studied. Although our study suggests that abnormal lipid metabolism and liver steatosis precede the subsequent development of insulin resistance, it is possible that NCOA3-mediated alteration of IRS2 signaling could also contribute to the hepatic insulin resistance observed. Hence, further studies will be needed to determine if ULK1 deficiency induces gluconeogenesis and insulin resistance in part by altering the phosphorylation of IRS-2.

The present study does not elucidate the mechanisms underlying ULK1 repression in MASLD and MASH. Our data in mice and new data in human cohorts indicate that the reduction in ULK1 likely occurs on the basis of transcriptional repression. Although much is known about the regulation of ULK1 activity by phosphorylation, less is known about the transcriptional regulation of ULK1 or the regulation of its mRNA, including half-life and stability specifically in the liver. However, multiple mechanisms have been reported to regulate ULK1 gene expression in diverse cell types, as recently reviewed (70). Future studies will pursue these mechanisms in the context of MASLD. We also note that hepatocyte-induced loss of ULK1 exacerbated obesity, by unclear mechanisms, and this enhanced obesity could also indirectly contribute to accelerating hepatic steatosis in this model.

In conclusion, this study establishes ULK1 as a key repressor of hepatic de novo lipogenesis in a manner independent of its roles in autophagy regulation. This kinase-dependent role of ULK1 in modulating NCOA3 phosphorylation plays a critical role in multiple mechanisms involved in MASLD, including lipogenesis, antioxidant defense, and inflammation. Thus, the ULK1-NCOA3 axis may represent a new frontier for therapeutic interventions against metabolic liver disease.

## Methods

Detailed methods and protocols can be found in Supplemental Methods.

### Sex as a biological variable.

For animal models, only male mice were examined, to reduce female sexual cycle–related variation.

### Donor characteristics.

The donors without steatosis were 3 men and 1 woman, aged 20–64 years, with BMI ranging from 21.5 to 29.96. The donors with steatosis (10%–70%) were 3 men and 1 woman, aged 17–58 years, with BMI ranging from 24.3 to 69.6.

### Statistics.

All data are presented as mean ± SEM. Statistical analyses were performed using GraphPad Prism, version 10.2.2. Differences between groups were assessed using Student’s 2-tailed *t* test, 1-way ANOVA, or 2-way ANOVA, followed by Tukey’s multiple comparisons post hoc test. *P <* 0.05 was considered statistically significant.

### Study approval.

All aspects of animal care and experiments were conducted in accordance with the *Guide for the Care and Use of Laboratory Animals* (National Academies Press, 2011) of the NIH and approved by the animal research committees of UCLA (permit ARC-2003-105) and the University of Iowa (permit 9041709-042). Human liver specimens used in this study were provided by the University of Kansas Liver Center Tissue Bank. The use of human liver tissues was approved by the University of Iowa IRB as nonhuman subjects research. None of the donors had diabetes.

### Data availability.

All primary data not deposited in a public repository are maintained at UCLA and are available upon reasonable request to the corresponding author. A Supporting Data values file containing all the supporting data used in graphs and RNA-Seq raw data files are available as Supplemental Materials. The mass spectrometry proteomics data have been deposited to the ProteomeXchange Consortium via the PRIDE partner repository (dataset identifier PXD072418).

## Author contributions

YDK contributed to the study conceptualization; resources; data curation, formal analysis, validation, and visualization; project administration and supervision; and wrote the first draft of the manuscript and reviewed and edited it. RTC, ASN, MG, CG, ZVC, TSV, JH, AM, EDK, RJ, JS, and MB contributed to the investigation; data curation, formal analysis, validation, and visualization. DD, NML, SS, YD, CP, IAA, ETW, DFD, AH Jr., JNB, and AGM contributed to the study methodology; investigation; data curation, formal analysis, validation, and visualization; and software. LY, ROP, TT, and RS contributed to the study conceptualization and resources. EDA contributed to the study conceptualization; resources; supervision; project administration; formal analysis; validation; visualization; and funding acquisition; and wrote the first draft of the manuscript and reviewed and edited it.

## Conflict of interest

The authors have declared that no conflict of interest exists.

## Funding support

American Heart Association (AHA) Strategically Focused Research Network (grant 20SFRN35120123 to YDK and EDA, the latter of whom is an established investigator of the AHA).

- National Science Foundation Graduate Research Fellowship Program (grant DGE-2444110 to YDK).
- UCLA AIDS Institute.
- The James B. Pendleton Charitable Trust.
- The McCarthy Family Foundation.

## Supplementary Material

Supplemental data

Supplemental data set 1

Supplemental data set 10

Supplemental data set 11

Supplemental data set 2

Supplemental data set 3

Supplemental data set 4

Supplemental data set 5

Supplemental data set 6

Supplemental data set 7

Supplemental data set 8

Supplemental data set 9

Unedited blot and gel images

Supporting data values

## Version 1 04/02/2026

In-Press Preview

## Version 2 06/01/2026

Electronic publication
