# Supplementary material for: The ULK1-NCOA3 axis restrains de novo lipogenesis and prevents diet-induced steatohepatitis and fibrosis in mice
Source: J Clin Invest. 2026 Apr 2;136(11):e191101. doi: 10.1172/JCI191101 (PMC13221236; doi:10.1172/JCI191101)
Supplement: Supplemental data [file jci-136-191101-s154.pdf]

Supplemental Information

The ULK1-NCOA3 axis restrains *de novo* lipogenesis and prevents diet-induced steatohepatitis and fibrosis in mice

Young Do Koo[1,2], Romilia Tatiana Castillo[2], Asha Sukumaran Nair[2], Michael Garneau[2], Chad Gochee[2], Zachary V. Campbell[2], Tashya Shreyas Vakil[1], Jua Ha[1], Alex Marti[2], Jamie Soto[2,3,4], Debajyoti Das[5,6], Nuria Martinez-Lopez[5,6], Shipra Sharma[7,8], Yennifer Delgado[7,8], Callie Phung[7,8], Immy A. Ashley[7,8], Edmund D Kapelczak[9], Rashel Jacobo[9], Eric T. Weatherford[2,3], Dao-Fu Dai[10], Jihane N. Benhammou[5,611], Andrea G. Marshall[12], Antentor Hinton Jr.[12], Ling Yang[2,13], Renata O. Pereira[2], Tara TeSlaa[9], Mehdi Bouhaddou[7,8], Rajat Singh[5,6], E. Dale Abel[1,2].

[1]Division of Endocrinology, Diabetes and Metabolism, Department of Medicine, David Geffen School of Medicine and UCLA Health, University of California-Los Angeles, Los Angeles, CA, 90095, USA.

[2]Fraternal Order of Eagles Diabetes Research Center, Roy J. and Lucille A. Carver College of Medicine, University of Iowa, 169 Newton Road, 4338 PBDB, Iowa City, IA, 52242, USA.

[3]Mouse Metabolic Phenotyping Core, Carver College of Medicine, University of Iowa, Iowa City, IA, 52242, USA.

[4]Center for Translational Science, Florida International University, Port Saint Lucie, FL, 34987, USA

[5] Vatche and Tamar Manoukian Division of Digestive Diseases, David Geffen School of Medicine, University of California-Los Angeles, Los Angeles, CA, 90095, USA.

[6] Comprehensive Liver Research Center, University of California-Los Angeles, Los Angeles, CA, 90095, USA.

[7] Department of Microbiology, Immunology, and Molecular Genetics (MIMG), University of California, Los Angeles, LA, USA.

[8] Institute for Quantitative and Computational Biosciences, University of California, Los Angeles, Los Angeles, CA, USA.

[9]Department of Molecular and Medical Pharmacology, University of California Los Angeles, Los Angeles, CA, USA.

[10] Department of Pathology, Johns Hopkins University School of Medicine, Baltimore, MD

[11] Veterans' Affairs Greater Los Angeles Healthcare System; Los Angeles, USA.

[12] Department of Molecular Physiology and Biophysics, Vanderbilt University, Nashville, TN 37232, USA.

[13]Department of Anatomy and Cell Biology, University of Iowa Carver College of Medicine, Iowa City, IA, USA.

2-Way ANOVA statistical Information for main figures

Figure 1: A significant treatment effect for CQ was present for LC3B-I ($p=0.0298$), LC3B-II ($p=0.0001$), and p62/SQSTM1 ($p<0.0001$). A significant genotype effect is observed for LC3B-I ($p=0.0035$), LC3B-II ($p<0.0001$), and p62/SQSTM1 ($p<0.0001$) in ULK1 KO compared to WT. A Genotype x treatment interaction was observed following CQ treatment in ULK1 KO for LC3B-II ($p=0.0103$). (G–H) Data were analyzed by two-way ANOVA to assess the effects of genotype and diet, followed by Tukey's post-hoc test for multiple comparisons and actual p-values are shown. A significant treatment effect for HFD was present for autophagosome number ($p<0.0001$) and LC3B expression ($p=0.0001$). A significant genotype effect is present for autophagosome number ($p<0.0001$) and LC3B expression ($p=0.0013$) in ULK1 KO compared to WT. A genotype × treatment interaction was not observed following HFD feeding in ULK1 KO for autophagosome number and LC3B expression.

Figure 2: A significant treatment effect for HFD was present for ULK1 expression ($p=0.0007$) (A), body weight (B), absolute fat mass (C), relative fat mass (C), liver weight (E), liver triglyceride ((F), liver cholesterol (F), lipid droplet number (H), insulin (I), cholesterol (J), triglyceride (K) (all $p<0.0001$), and free fatty acid ($p=0.0005$) (L). A significant genotype effect was present for ULK1 expression (A), body weight (B), absolute fat mass (C), relative fat mass (C), liver weight (E) (all $p<0.0001$), liver triglyceride ($p=0.0005$) (F), liver cholesterol ($p=0.0002$) (F), lipid droplet ($p<0.0001$) (H), insulin ($p=0.0003$) (I), cholesterol ($p=0.0001$) (J), triglyceride ($p=0.0069$) (K), and free fatty acid ($p=0.0003$) (L) in ULK1 KO compared to WT. A genotype × treatment interaction was observed following HFD feeding in ULK1 KO for ULK1 expression ($p=0.0034$) (A), body weight ($p<0.0001$) (B), absolute fat mass ($p=0.0011$) (C), liver weight ($p=0.0021$) (E), liver triglyceride ($p=0.0115$) (F), and liver cholesterol ($p=0.0286$) (F).

Figure 3: A significant treatment effect for HFD was present for SREBP1 RNA levels ($p<0.0001$) (A), SCD1 RNA levels ($p=0.0004$) (A), SREBP1 protein levels ($p=0.0243$) (B) and FASN protein levels ($p=0.0119$) (B). A significant genotype effect was present for RNA levels of SCD1, FASN, protein levels of SREBP1, SCD1 and FASN (all $p\leq0.0001$) in ULK1 KO compared to WT. A genotype × treatment interaction was observed following HFD feeding in ULK1 KO for SREBP1 protein levels ($p=0.029$) (B). For AKT phosphorylation, a significant treatment effect for insulin was present AKT ($p<0.0001$) and there were no significant genotype effects or genotype × treatment interactions.

Figure 5: A significant treatment effect for HFD is present for absolute fat mass ($p<0.0001$) (C), SREBP1 protein levels ($p=0.0194$) (D), SCD1 protein levels ($p=0.0454$) (D), FASN protein levels ($p=0.0002$) (D), insulin (G), triglyceride (G), cholesterol (G), free fatty acid (G) (all $p\leq0.0001$). A significant genotype effect is present for absolute fat mass (C), SREBP1 protein levels (D), SCD1 protein levels (D), FASN protein levels (D), insulin (G), triglyceride (G), cholesterol (G), free fatty acid (G) (all $p<0.0001$). A genotype × treatment interaction is observed for SCD1 protein levels ($p=0.003$) (D) and FASN protein levels ($p<0.0001$) (D). A significant treatment effect for SI-2 is present for NCOA3 ($p<0.0001$) (I), SREBP1 ($p=0.0004$) (I), SCD1 ($p=0.0001$) (I), and FASN ($p=0.003$) (I). A significant genotype effect is present for ULK1 (I), NCOA3(I), SREBP1 (all $p<0.0001$) (I), SCD1

(p=0.0033) (I), and FASN (p<0.0001) (I) in ULK1 KO compared to WT. A genotype × treatment interaction is observed following SI-2 treatment for SREBP1 (p=0.0003) (I), SCD1 (p=0.0488) (I), and FASN (p=0.001) (I).

Figure 6: A significant treatment effect for HFD is present for NAS score (p<0.0001). A significant genotype effect is present for NAS score (p<0.0001). No genotype × treatment interaction for NAS score was observed.

Figure 7: A significant treatment effect for HFD is present for NRF protein levels (A), Keap1 protein levels (A), TNFα mRNA levels (E) and ROS levels (all p<0.0001) (B), IL-4 mRNA levels (p=0.0059) (E), IL-6 mRNA levels (p=0.0016) (E), CSF1 mRNA levels (E), CCL2 mRNA levels (E), and CCL3 mRNA levels (all p<0.0001) (E), CCL4 mRNA levels (p=0.0007) (E), ALT (p=0.0044) (G), and AST (p=0.002) (G). A significant genotype effect is present for NRF protein levels (A), Keap1 protein levels (A), ROS levels (B) and TNFα mRNA levels (all p<0.0001) (E), IL-4 mRNA levels (p=0.0053) (E), IL-6 mRNA levels (p=0.0019) (E), ITFγ mRNA levels (p=0.0005) (E), CSF1 mRNA levels (p<0.0001) (E), CCL2 mRNA levels (p=0.0003) (E), CCL3 mRNA levels (p=0.0312) (E), CCL4 mRNA levels (E), ALT (G), and AST (all p≤0.0001). A genotype × treatment interaction is observed for NRF protein levels (p=0.0002) (A), Keap1 protein levels (p=0.0003) (A), TNFα mRNA levels (p<0.0001) (E), ITFγ mRNA levels (p=0.0374) (E), CSF1 mRNA levels (p<0.0001) (E), CCL2 mRNA levels (p=0.0106) (E), and CCL4 mRNA levels (p=0.0298) (E). A significant treatment effect for SI-2 is present for ROS levels (p=0.0005) (D), TNFα mRNA levels (p=0.0003) (F), IL-4 mRNA levels (p=0.0012) (F), ITFγ mRNA levels (p=0.0206) (F), CSF1 mRNA levels (p=0.0439) (F), and CCL4 mRNA levels (p=0.0081) (F). A significant genotype effect is present for NRF protein levels (p=0.0001) (C), Keap1 protein levels (p=0.002) (C), ROS levels (p=0.0007) (D), TNFα mRNA levels (p=0.0004) (F), IL-4 mRNA levels (p=0.0009) (F), IL-6 mRNA levels (p=0.0207) (F), CCL2 mRNA levels (p=0.0452) (F), and CCL5 mRNA levels (p=0.0452) (F). A genotype × treatment interaction is observed for NRF protein levels (p=0.0037) (C), Keap1 protein levels (p=0.0174) (C), CSF1 mRNA levels (p=0.0028) (F), and CCL4 mRNA levels (p=0.0014) (F).

Supplemental Figures

Supplemental Figure 1. Related to Figure 1. ATG protein levels in livers of high-fat-fed mice and autophagy flux when ULK1 is silenced in cultured hepatocytes. Protein levels of ATG5, ATG7, and ATG13 were measured in liver extracts from NCD fed mice (n=5) and 60% HFD fed mice (n=5). All data are represented as the mean ± SEM. Significant p values shown, vs NCD by Student's t-test. (B) Numbers of autophagosomes and lysosomes were measured in transmission electron micrographs of Hepa1c1c7 cells incubated with siControl or siULK1. Blue arrows indicate autophagosomes, specifically those with cellular content within them, and green arrows indicate lysosomes. (C) Protein levels of autophagy formation markers in siControl and siULK1 cells treated with 80uM of CQ for 6h. All data are represented as the mean ± SEM. (A and B) Two-tailed Student's t-test. (C) Data were analyzed by two-way ANOVA to assess the effects of genotype and CQ treatment, followed by Tukey's post-hoc test for multiple comparisons. Actual p-values are shown. A significant treatment effect for CQ is present for LC3B-II ($p<0.0001$) and ULK1 ($p<0.0001$) (C). A significant genotype effect is observed for LC3BI ($p=0.0009$), LC3BII ($p<0.0001$), p62/SQSTM1 ($p=0.0025$), and ULK1 ($p=0.0002$) in siULK1 compared to siControl cells (C). A Genotype × treatment effect is observed following CQ treatment in siULK1 cells for LC3BI ($p=0.0317$), p62/SQSTM1 ($p=0.0248$), and ULK1 ($p=0.0006$) (C).

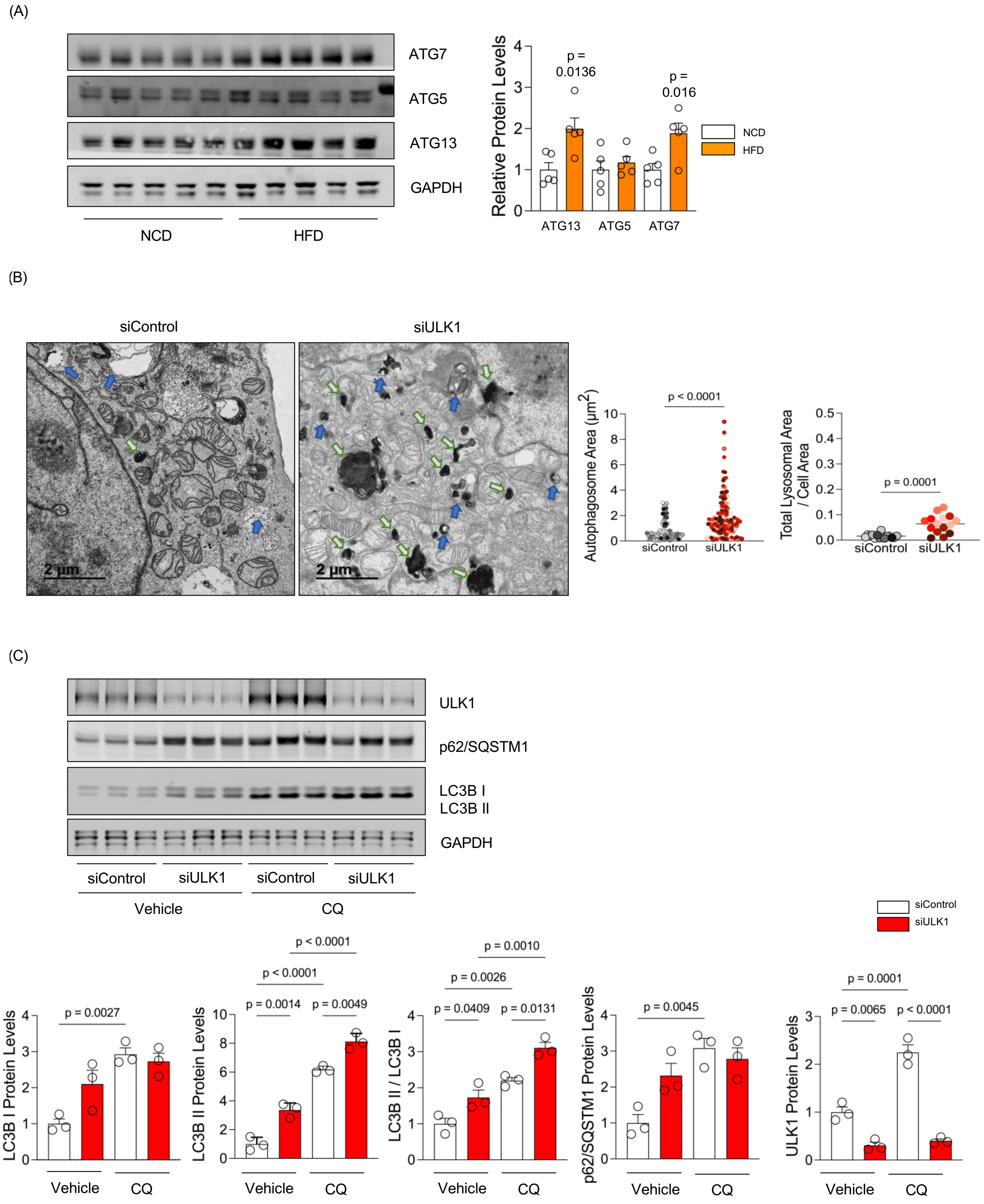

ıpplemental figure 1. Related to Figure 1. ATG protein levels in livers of high-fat-fed mice and autophagy x when ULK1 is silenced in cultured hepatocytes.

Supplemental Figure 2. Related to Figure 2. Lean mass, energy expenditure, cumulative food intake and energy balance in hepatocyte-specific ULK1 knockout mice. (A) Lean mass of 18-week-old WT (n=5-10) and L-ULK1 KO mice (n=5-10) after 12-weeks of NCD and HFD feeding. All data represent the mean ± SEM, and data were analyzed by two-way ANOVA to assess the effects of genotype and diet, followed by Tukey's post-hoc test for multiple comparisons. Actual p-values are shown. A significant treatment effect for HFD is present for relative lean mass ($p<0.0001$) (A). A significant genotype effect is observed for absolute and relative lean mass ($p=0.0166$) and ($p=0.0128$) respectively in ULK1 KO compared to WT (A). A Genotype × treatment interaction is not observed. (B) Cumulative and total energy expenditure, (C) oxygen consumption, (D) cumulative food consumption, and (E) cumulative energy balance was measured following 12 weeks of HFD feeding in a CLAMS metabolic chamber. (F) Oxygen consumption was measured in frozen liver tissues of ULK1 liver-specific knockout mice and WT controls under high fat diet conditions. All data are represented as the mean ± SEM. (A, C, D, E, and F) Two-tailed Student's t-test.

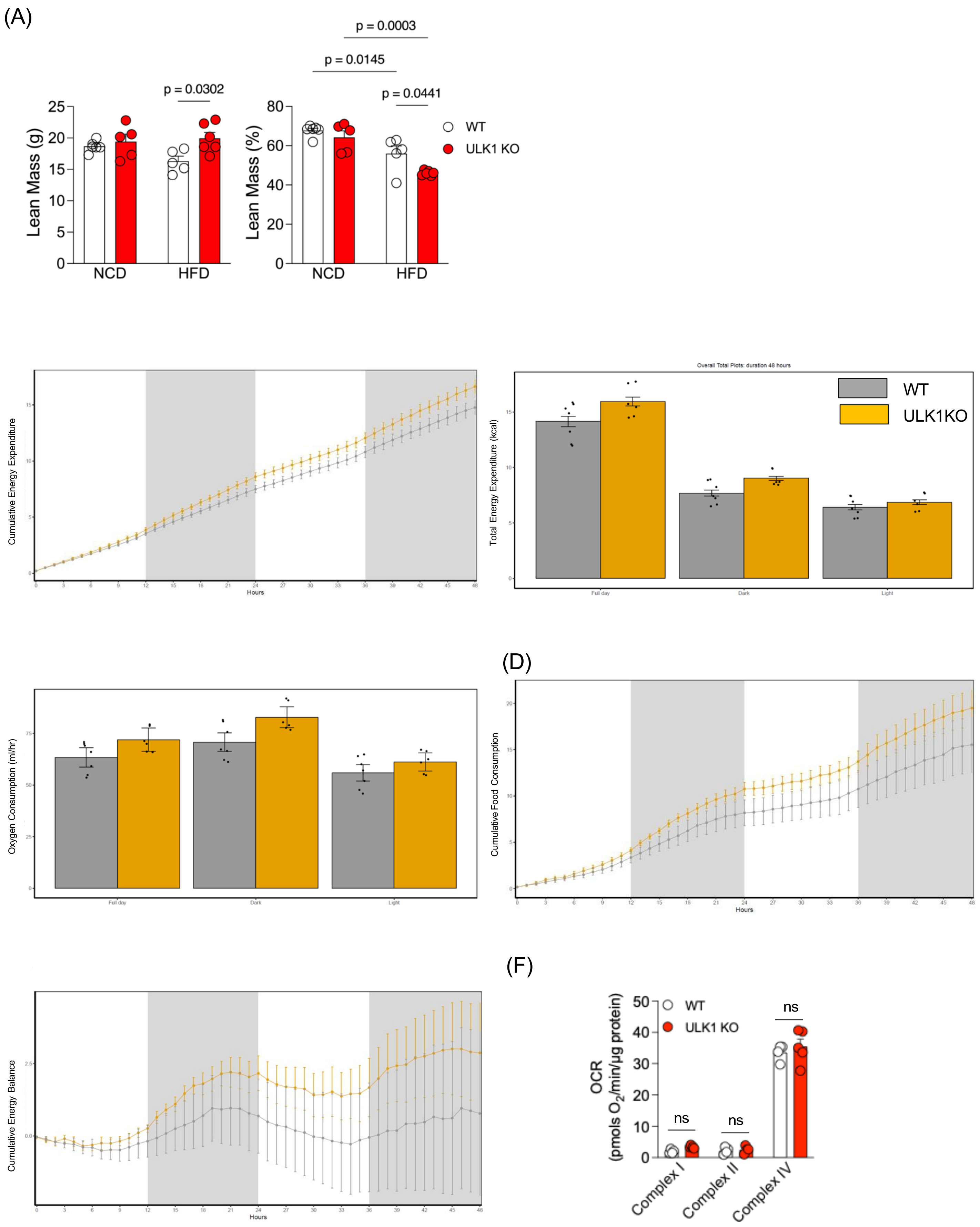

ıpplemental figure 2. Related to Figure 2. Lean mass, energy expenditure, cumulative food intake and ıergy balance in hepatocyte-specific ULK1 knockout mice.

Supplemental Figure 3. Related to Figure 2. Hepatocyte ULK1 deficiency impairs glucose tolerance, promotes insulin resistance and increases hepatic gluconeogenesis. (A) Glucose tolerance test (GTT) and (B) insulin tolerance tests (ITT) were conducted in WT (n=7-8) and L-ULK1 KO (n=6-9) mice following 12-weeks of NCD and HFD feeding. $^{*}p<0.05$ vs WT of NCD and $^{\#}p<0.05$ vs WT of HFD. (C) Phosphorylation levels of AKT were measured in WT (n=5) and L-ULK1 KO (n=5) mice following 12-weeks of NCD and HFD feeding. The values obtained in the tissues from WT mice were set to 1. (D) Pyruvate tolerance test (PTT) was conducted in WT (n=7-8) and L-ULK1 KO (n=6-9) mice following 12-weeks of NCD and HFD feeding. $^{*}p<0.05$ vs WT of NCD and $^{\#}p<0.05$ vs WT of HFD. (E) mRNA levels of PEPCK and G6Pase in liver tissues of WT (n=4) and L-ULK1 KO (n=8) under NCD conditions. The AUC graphs are depicted as fold change relative to NCD WT values, arbitrarily set to a mean of 100 (A, B, D). Data were analyzed by two-way ANOVA to assess the effects of genotype and diet, followed by Tukey's post-hoc test for multiple comparisons. Significant genotype and treatment effects (all $p<0.001$) were observed for all AUCs (A,B,D). Significant treatment and genotype interactions were noted for GTT ($p=0.0310$), ITT ($p=0.0105$) and PTT ($p=0.0492$) AUCs. Data in C, (mean ± SEM) were analyzed by two-way ANOVA to assess the effects of genotype, insulin, and diet, followed by Tukey's post-hoc test for multiple comparisons. Actual p-values are shown. A significant treatment effect for insulin and HFD ($p<0.0001$) (C), and a significant genotype effect ($p<0.0001$) was observed. A Genotype × treatment interaction was observed following insulin and HFD treatment in ULK1 KO ($p=0.0227$) (C). (E).Two-tailed Student's t-test and actual p-values are shown.

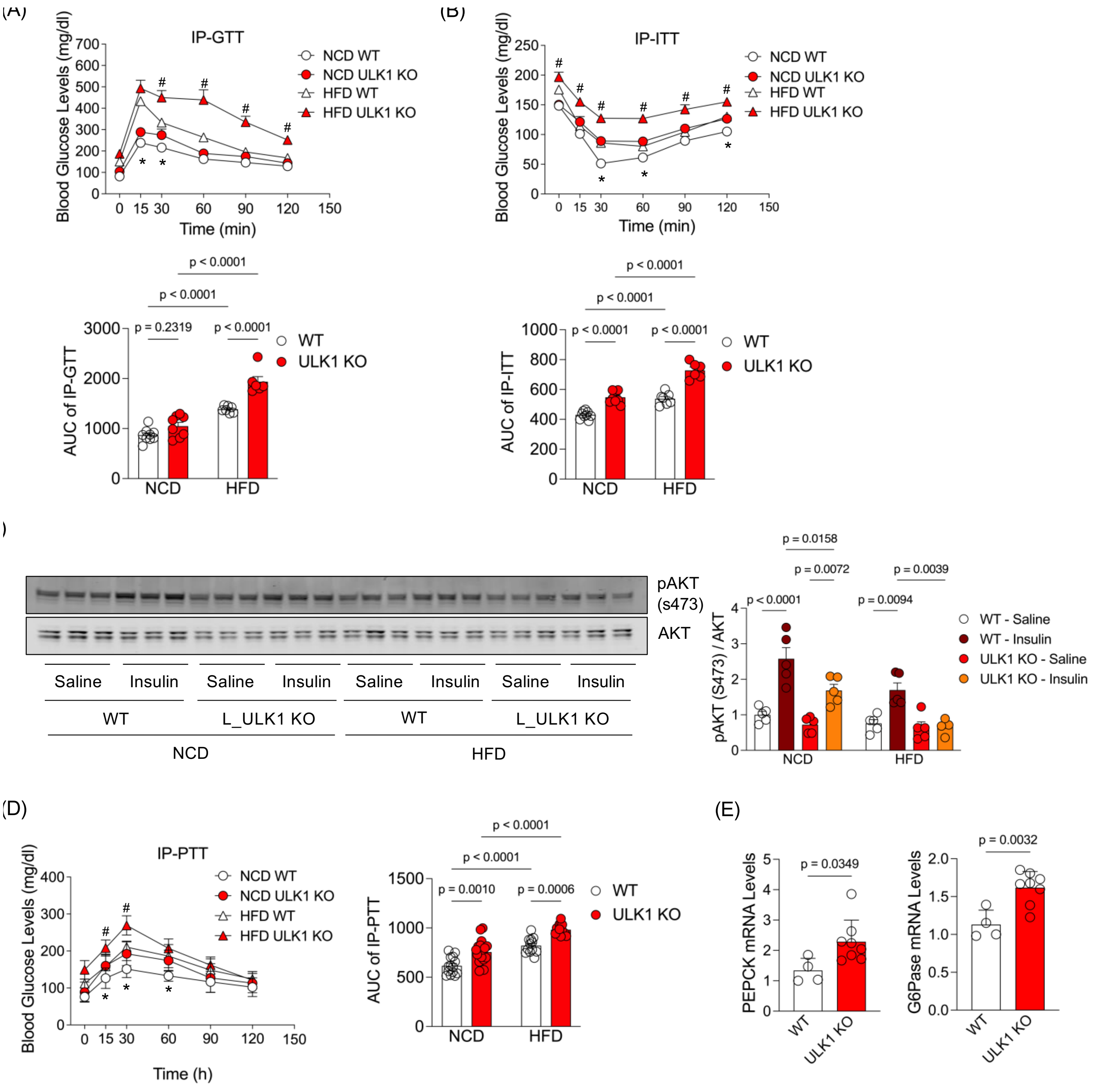

ıpplemental figure 3. Related to Figure 2. Hepatic ULK1 deficiency impairs glucose tolerance, promotes ısulin resistance and increases hepatic gluconeogenesis.

Supplemental Figure 4. Related to Figure 2. Hepatocyte deficiency of ULK2 does not induce obesity, glucose intolerance, insulin resistance or MASH. (A) mRNA expression of ULK2 in liver tissues of WT and L-ULK2 KO mice. WT and L-ULK2 KO mice were fed a NCD and HFD for 12 weeks. (B) Body weights, (C) liver weight of 18-week-old WT (n=6) and L-ULK2 KO mice (n=8) following 12-weeks of NCD and HFD feeding. (D) Glucose tolerance (GTT) and (E) insulin tolerance tests (ITT) were conducted in WT (n=6) and L-ULK2 KO (n=8) following 12-weeks of NCD and HFD feeding. $^{*}p<0.05$ vs WT of NCD and $^{\#}p<0.05$ vs WT of HFD. Levels of (F) insulin, (G) triglyceride, (H) cholesterol, (I) ALT, and (J) AST in WT (n=4) and L-ULK2 KO mice (n=4) following 12-weeks of NCD and HFD feeding. All data represent the mean ± SEM. (A, B, D, and E) Two-tailed Student's t-test. (C, F, G, H, I, and J) Data were analyzed by two-way ANOVA to assess the effects of genotype and diet, followed by Tukey's post-hoc test for multiple comparisons. Actual p-values are shown. A significant treatment effect for HFD is present for body weight (p<0.0001), liver weight (p<0.0001), IP-GTT (p<0.0001), IP-ITT (p<0.0001), insulin (p<0.0001), triglyceride (p=0.0007), cholesterol (p=0.0091), ALT (p<0.0003), and AST (p<0.0001) of ULK2 KO. A significant genotype effect for HFD was not observed in ULK2 KO compared to WT. A Genotype × treatment effect was not observed following HFD feeding in ULK2 KO.

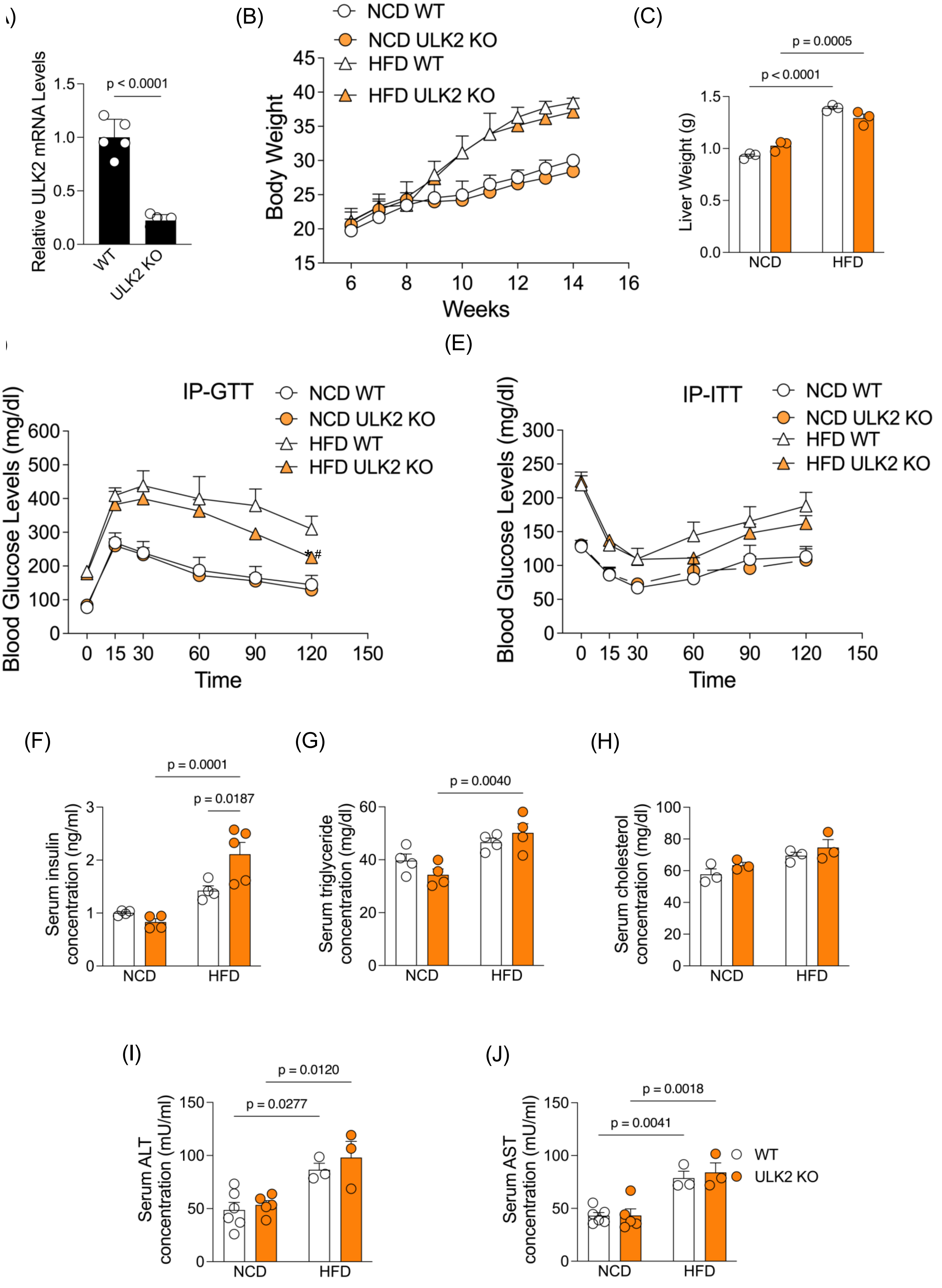

ıpplemental figure 4. Related to Figure 2. Hepatocyte deficiency of ULK2 does not induce obesity, glucose olerance, insulin resistance or MASH.

Supplemental Figure 5. Related to Figure 2. Knockout of ULK1 and ULK2 in hepatocytes phenocopies hepatocyte-specific ULK1 knockout. WT and L-ULK1/2 KO mice were fed a NCD and HFD for 12 weeks. (A) Body weights, (B) liver weight of 18- week-old WT (n=7) and L-ULK1/2 KO mice (n=7) and (C) Protein levels of lipogenic regulators in liver tissues of WT (n=3) and L-ULK1/2 KO (n=3) following 12-weeks of NCD and HFD feeding. (D) Glucose tolerance (GTT) and (E) insulin tolerance tests (ITT) were conducted in WT (n=6) and L-ULK1/2 KO (n=6) mice following 12-weeks of NCD and HFD feeding. Levels of (F) cholesterol, (G) triglyceride (H) insulin, (I) free fatty acids, (J) ALT, and (K) AST of WT (n=4) and L-ULK1/2 KO mice (n=4) following 12-weeks of NCD and HFD feeding. All data represent the mean ± SEM. (A, D, and E) Two-tailed Student's t-test. $^{*}p<0.05$ vs WT on NCD and $^{\#}p<0.05$ vs WT on HFD. (B, F, G, H, I, J, and K) Data were analyzed by two-way ANOVA to assess the effects of genotype and diet, followed by Tukey's post-hoc test for multiple comparisons. Actual p-values are shown. There was a significant treatment (diet) effect for the following parameters: body weight (p<0.0001) (A), liver weight (p<0.0001) (B), IP-GTT (p<0.0001) (D), IP-ITT (p<0.0001) (E), serum cholesterol (p=0.0003) (F), triglycerides (p<0.0001) (G), insulin (p<0.0001) (H), free fatty acids (p<0.0001) (I), and liver injury markers ALT (p<0.0001) (J) and AST (p=0.0004) (K). A significant genotype effect was observed for body weight (p<0.0001) (A), liver weight (p<0.0001) (B), IP-GTT (p=0.0063) (D), IP-ITT (p<0.0001) (E), serum cholesterol (p=0.002) (F), triglycerides (p<0.0001) (G), insulin (p=0.0038) (H), free fatty acids (p=0.0001) (I), ALT (p<0.0001) (J) and AST (p=0.0254) (K) in ULK1 KO compared to WT. A Genotype × treatment effect was observed for body weight (p<0.0001) (A), liver weight (p=0.0077) (B), IP-GTT (p=0.0111) (D), and insulin (p=0.0283) (H), following HFD feeding in ULK1 KO.

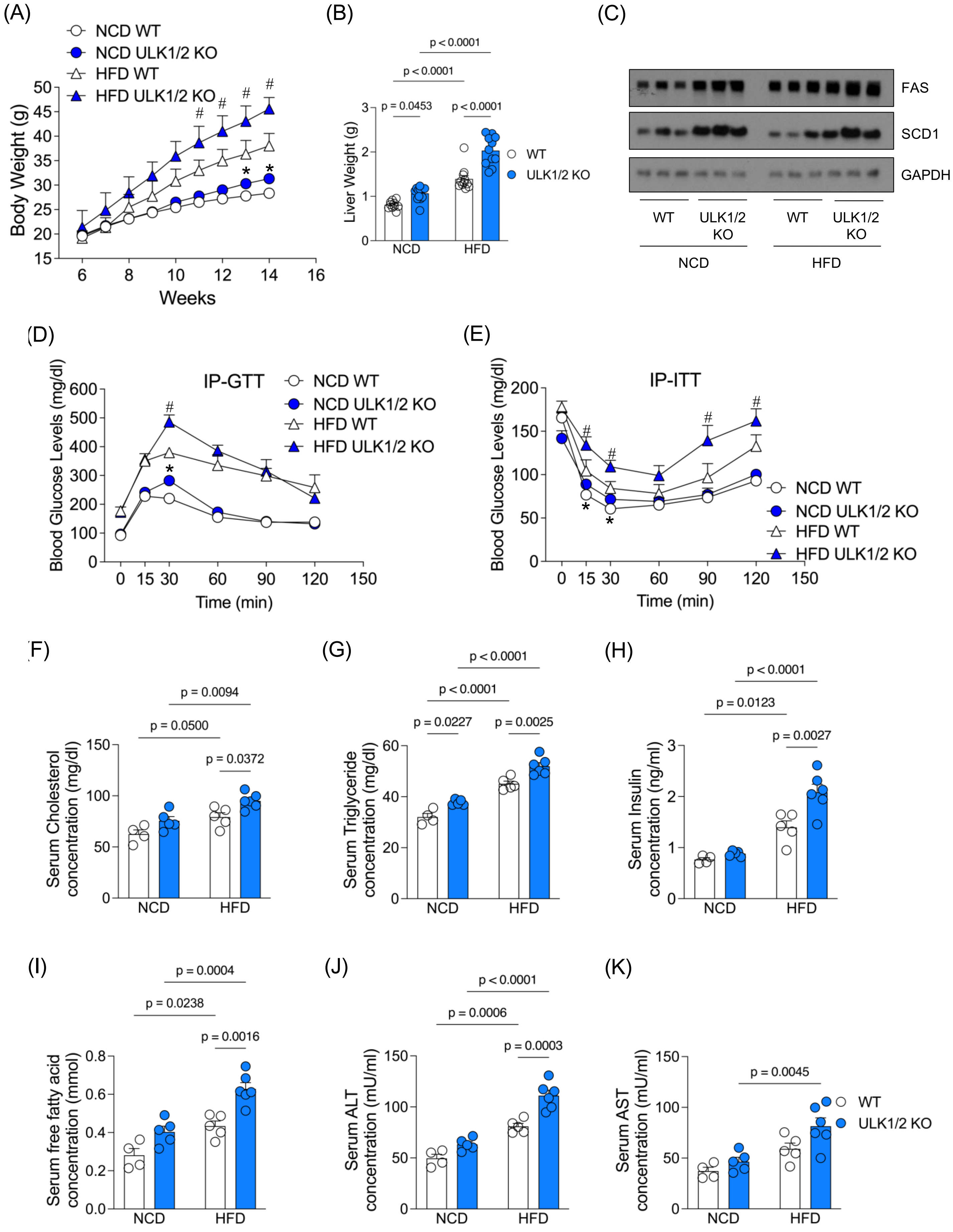

Supplemental figure 5. Related to Figure 2. Knockout of ULK1 and ULK2 in hepatocytes phenocopies hepatocyte-specific ULK1 knockout.

Supplemental Figure 6. Related to Figure 3. Loss of hepatocyte ULK1 promotes triglyceride secretion and increases de novo lipogenic flux. Hepatic triglyceride secretion of WT (n=4) and L-ULK1 KO (n=4) following 12-weeks of NCD and HFD feeding was measured at (A) 1, (B) 6, and (C) 24 h after poloxamer 407 IP injection. (D) Protein levels of MTP in liver tissues of WT (n=4-5) and L-ULK1 KO (n=4-5) following 12-weeks of NCD and HFD feeding. The relative values obtained in the tissues from WT mice on NCD were set to a mean of 1. All data represent the mean ± SEM. (A, B, and C) Two-tailed Student's t-test. **p*<0.05 vs WT on NCD and $^{\#}$*p*<0.05 vs WT on HFD. (D) Data were analyzed by two-way ANOVA to assess the effects of genotype and diet, followed by Tukey's post-hoc test for multiple comparisons. Actual p-values are shown. (D). A significant genotype effect is observed for MTP (p<0.0001) in ULK1 KO compared to WT (D). (E) Relative abundance of total isotopologues for C16:0 (palmitic acid), C16:1 (palmitoleic acid), C18:0 (stearic acid), and C18:1 (oleic acid) in livers from 4-week-old WT (purple) and ULK1 LKO (blue) mice. A significant increase in C16:1 was observed in ULK1 LKO compared to WT (p = 0.044). (F–I) Fractional isotopologue labeling [M+2–M+5] for palmitic acid (F), palmitoleic acid (G), stearic acid (H), and oleic acid (I). Data are presented as mean ± SEM; n = 5 per group. (E, F, G, H, and I) Two-tailed Student's t-test.

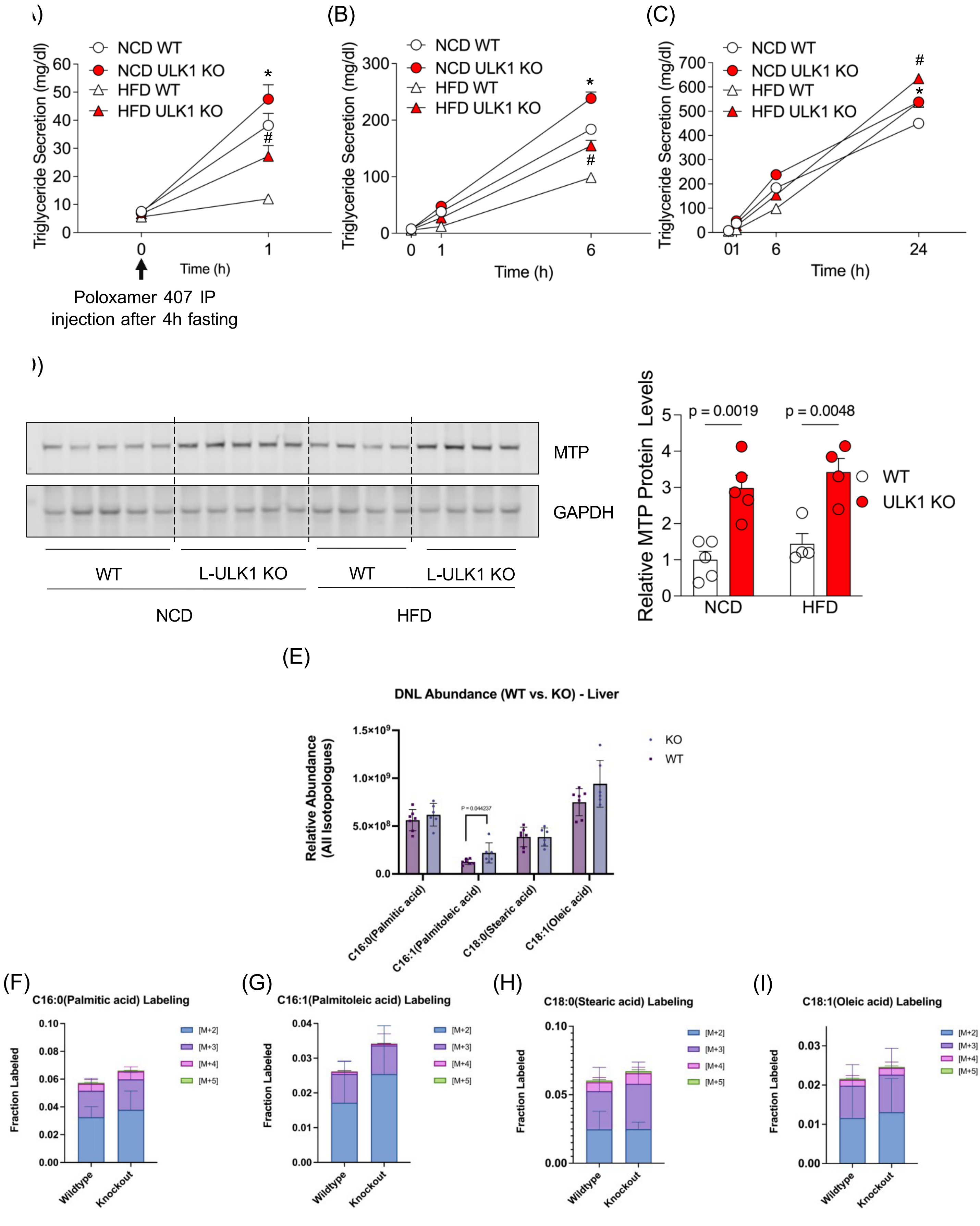

pplemental figure 6. Related to Figure 3. Loss of hepatocyte ULK1 promotes triglyceride secretion and creases de novo lipogenic flux.

Supplemental Figure 7. Related to Figure 3. Bulk RNA sequencing reveals global induction of lipogenic pathways in ULK1 deficient livers. (A) RNA seq was performed in liver tissues of 4-week-old WT (n=3) and L-ULK1 KO mice (n=3). (B) Directional changes in specific genes involved in the lipid metabolic process. (C) Pathway analysis describing most highly regulated signaling pathways identified.

A)

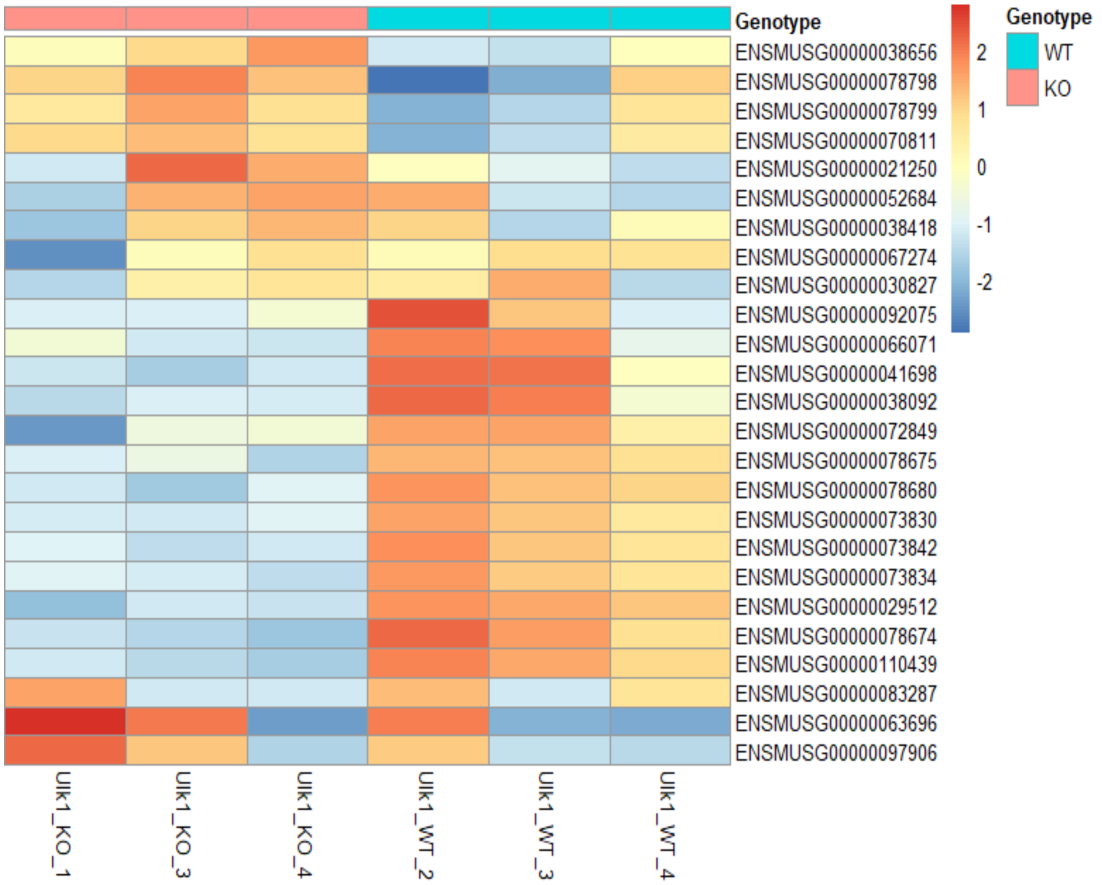

B)

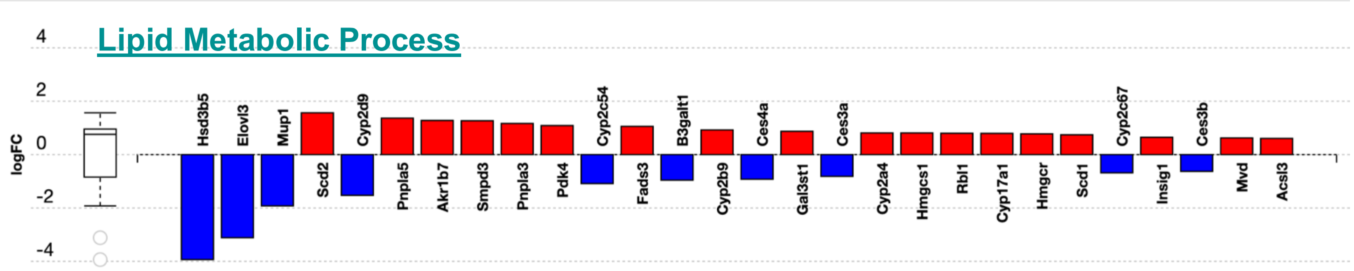

C)

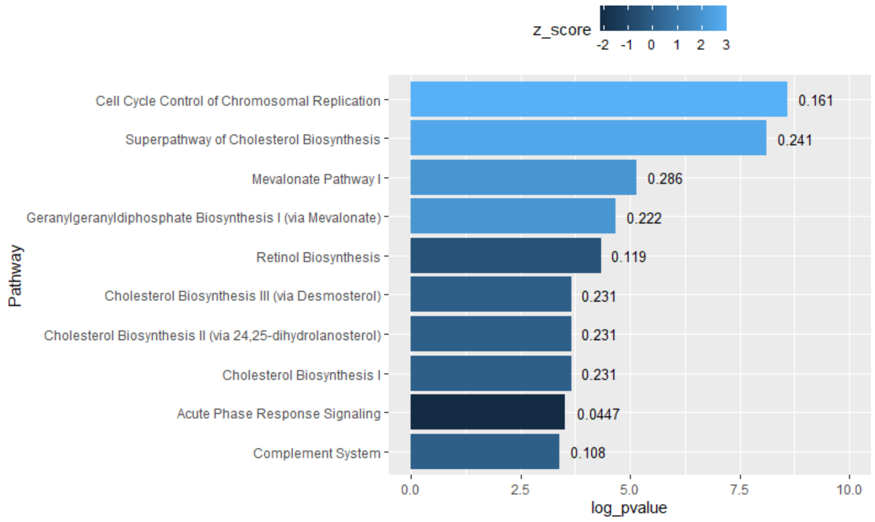

upplemental figure 7. Related to Figure 3. Bulk RNA sequencing reveals global induction of lipogenic thways in ULK1 deficient livers.

Supplemental Figure 8. Related to Figure 4. Schematic of in-vitro kinase assay for NCOA3 phosphorylation by ULK1. Schematic representation of the ADP-Glo™ kinase assay used to measure ULK1 kinase activity toward Myelin Binding Protein (MBP), $NCOA3^{520–710}$, and $NCOA3^{820–950}$ fragments. Recombinant ULK1 was incubated with the indicated substrates in the presence of ATP, and ADP production was quantified by luminescence detection.

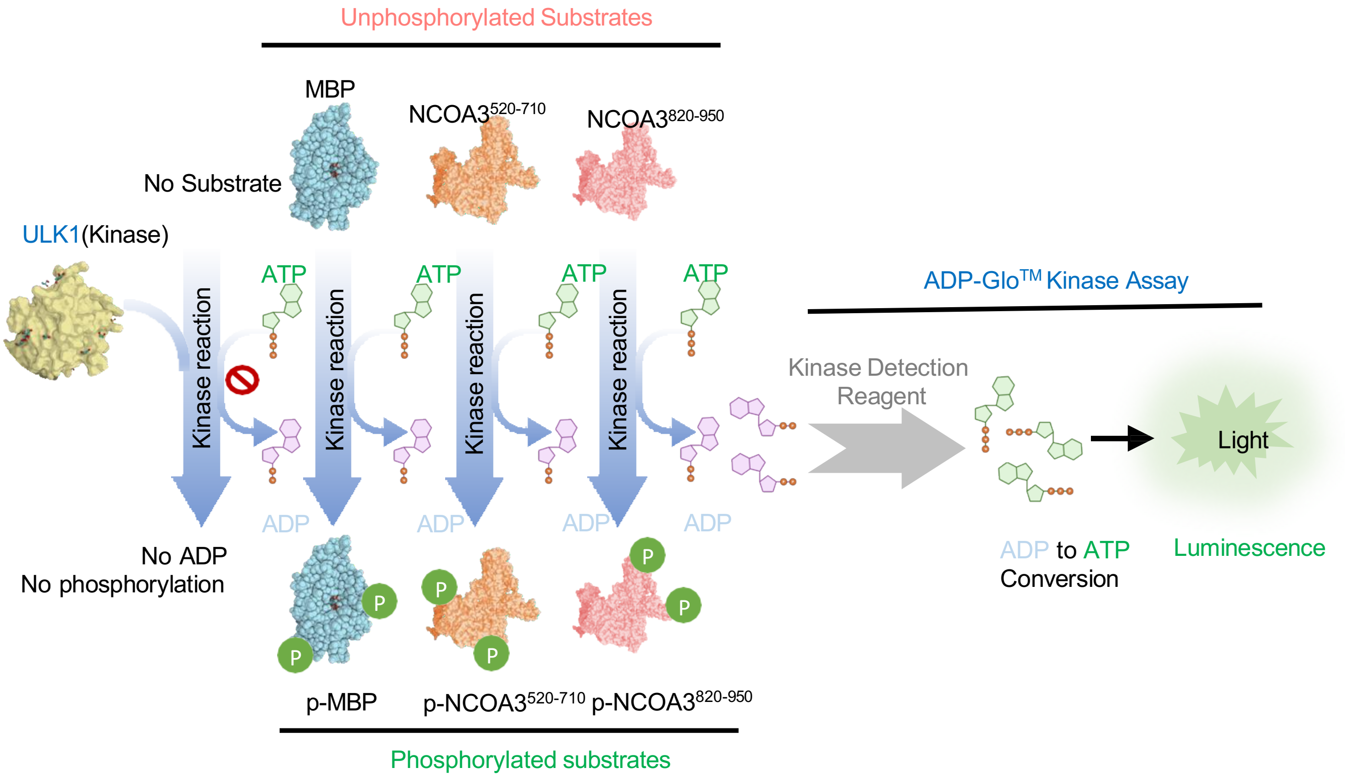

upplemental figure 8. Related to Figure 4. Schematic of in-vitro kinase assay for NCOA3 phosphorylation
ULK1.

Supplemental figure 9. Related to Figure 4. Proteomics analysis of NCOA3 peptides revealing no change in total NCOA3 levels following ULK1 deficiency (UKO). Heatmap analysis of NCOA3 peptides in liver samples from the indicated groups, WT and UKO following HFD and NCD feeding. Each column represents an individual biological replicate, and each row represents a detected NCOA3 peptide. Peptide intensity is color-coded as shown in the scale bar (blue to red, low to high).

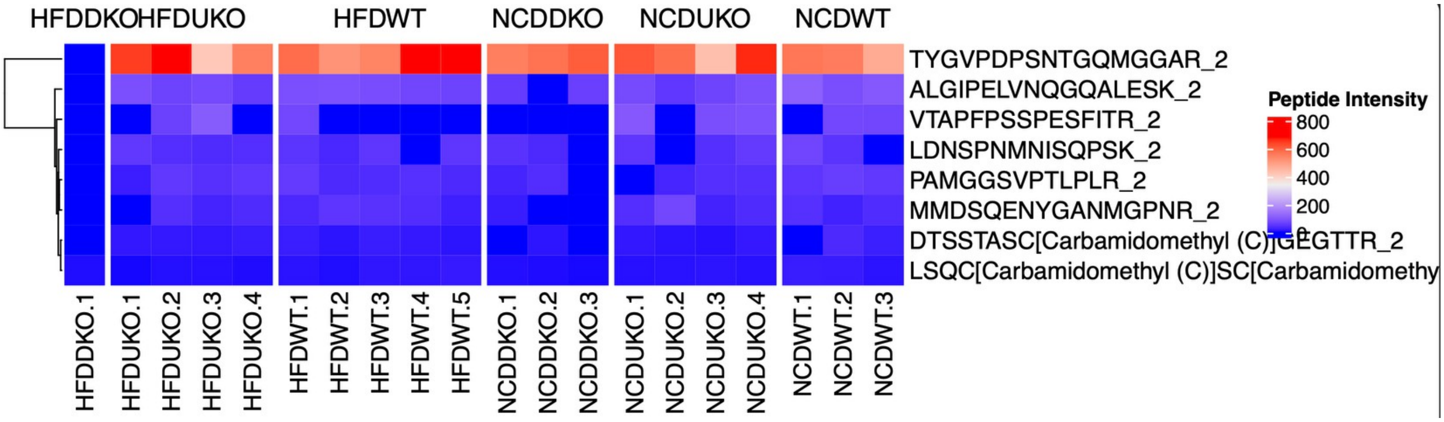

upplemental figure 9. Related to Figure 4. Proteomics analysis of NCOA3 peptides revealing no change in tal NCOA3 levels following ULK1 deficiency (UKO).

Supplemental Figure 10. Related to Figure 4. Effect of site-directed mutagenesis of putative ULK1 phosphorylation sites on lipogenic protein expression in cultured hepatocytes. (A) Predicted consensus ULK1 phosphorylation sites on NCOA3. Circles represent post-translational modifications (PTM) as follows: Blue - phosphorylation, Green - acetylation, Yellow - Ubiquitylation, Gray- Other PTM. Height of line (y-axis) corresponds to number of publications in the literature. (B) Hepa1c1c7 cells were transfected with control, siULK1, or site-specific phosphorylation deficient (Ser to Ala) mutants of putative ULK1 phosphorylation sites of NCOA3 as depicted. Protein levels of SREBP1, SCD1, and FASN were measured by western blot. The red bar indicates a statistically significant increase in lipogenic gene expression upon ULK1 knockdown compared to control. The orange bars represent NCOA3 wild-type (WT) or mutant overexpression conditions that did not show a statistically significant increase in lipogenic gene expression relative to control. The blue bars represent NCOA3 WT or mutant overexpression conditions that exhibited a statistically significant increase in lipogenic gene expression compared to control. All data represent the mean ± SEM. One-way ANOVA followed by Tukey post-hoc analysis. *$p<0.05$ versus Control.

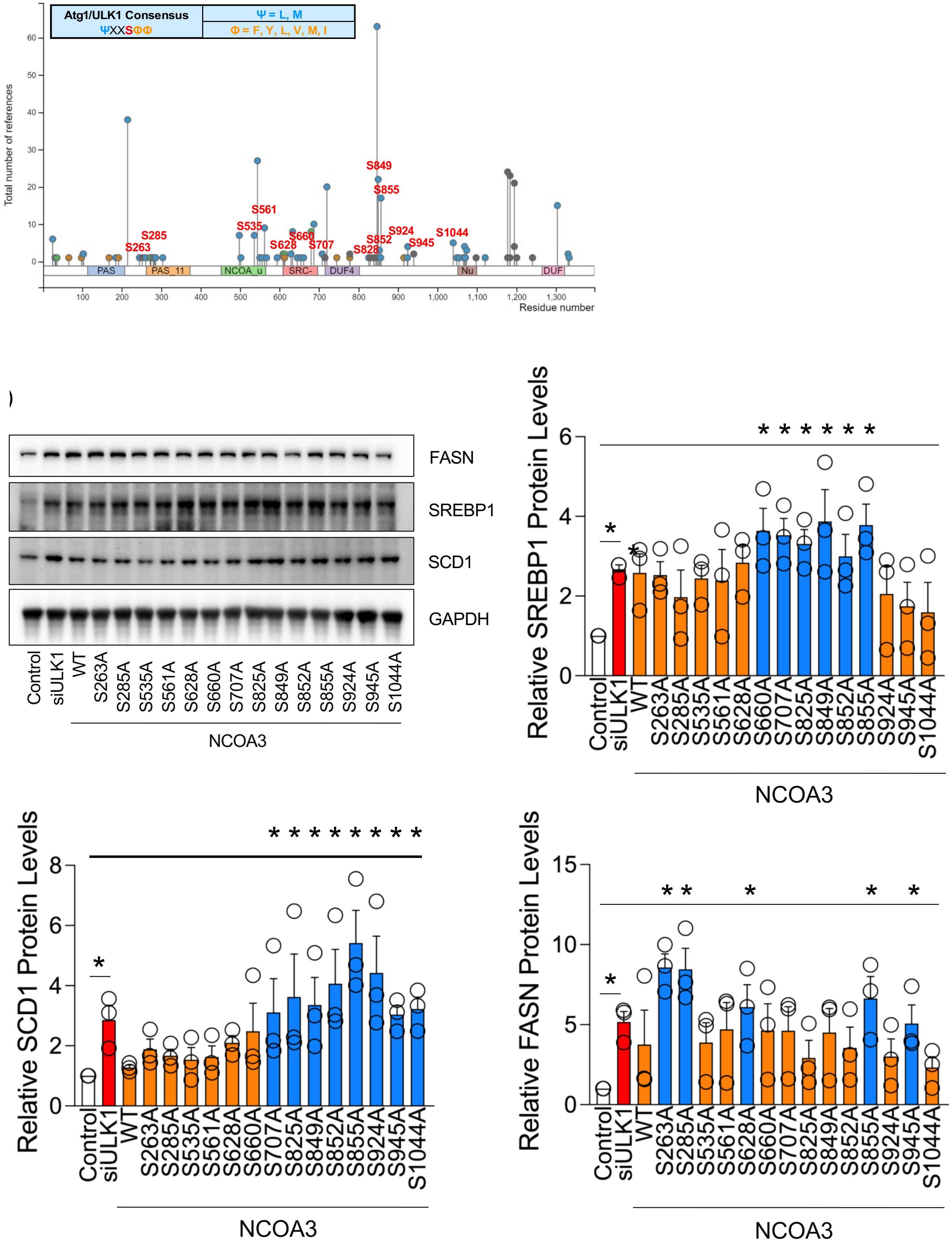

upplemental figure 10. Related to Figure 4. Effect of site-directed mutagenesis of putative ULK1 osphorylation sites on lipogenic protein expression in cultured hepatocytes.

Supplemental Figure 11. Related to Figure 5. Hepatocyte-specific NCOA3 deficiency reverses insulin resistance and impaired glucose homeostasis in hepatocyte-specific ULK1 deficient mice. Intraperitoneal glucose tolerance test (IP-GTT) and insulin tolerance test (IP-ITT) were conducted in WT (n=7), L-NCOA3KO (n=7), L-ULK1KO (n=8-9), and L-ULK1-NCOA3 KO (n=7) following 12-weeks of NCD and HFD feeding. Data for each time point were analyzed by one-way ANOVA to determine genotype-specific differences at specific times. *$p<0.05$ ULK1 KO vs WT and #$p<0.05$ ULK1 KO × NCOA3 KO vs ULK1 KO following 12-weeks of NCD or HFD feeding. The AUC graphs were analyzed by one-way ANOVA followed by Tukey's post-hoc test for multiple comparisons. P values for pairwise comparisons are shown.

(A)

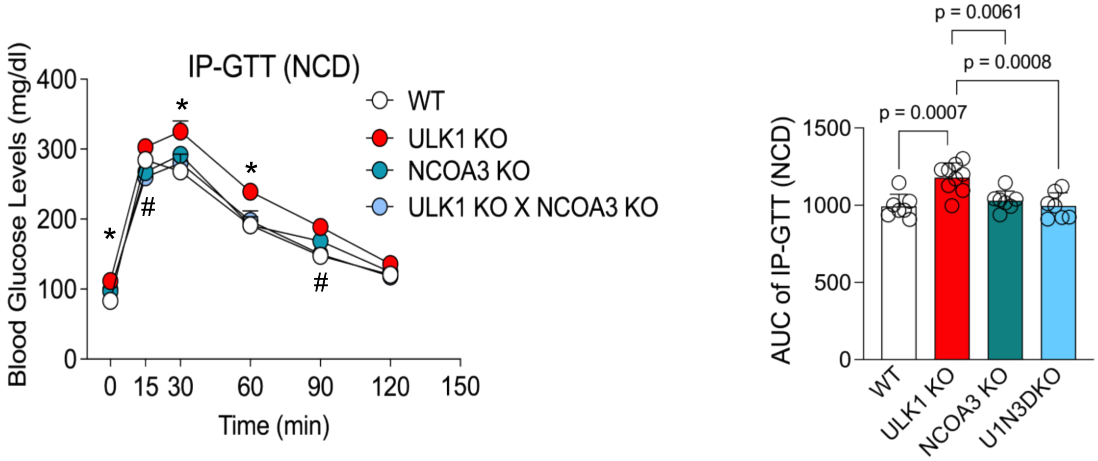

(B)

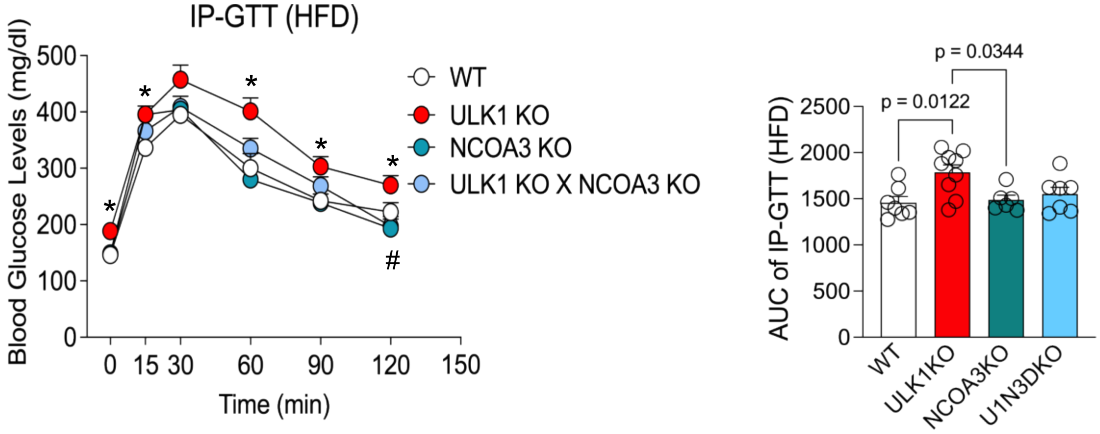

(C)

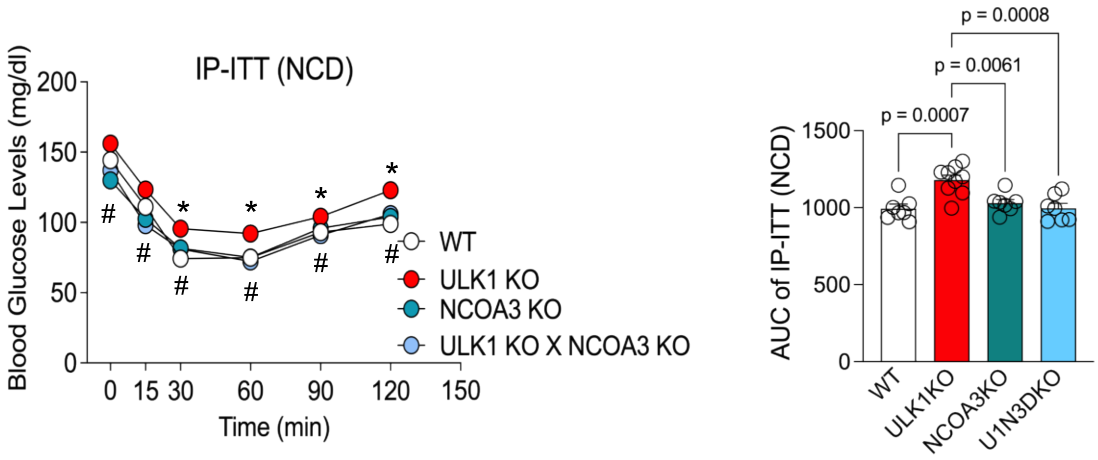

(D)

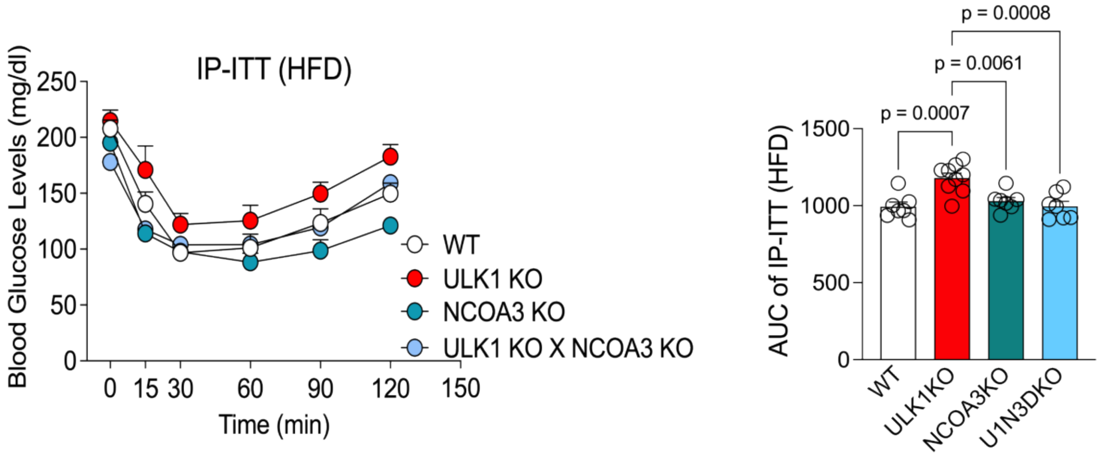

ıpplemental figure 11. Related to Figure 5. Hepatocyte-specific NCOA3 deficiency reverses insulin

Supplemental Figure 12. Related to Figure 5. In cultured hepatocytes, inhibition of NCOA3 by siRNA or by the pharmacological inhibitor SI-2, blocks the induction of lipogenic genes caused by ULK1 deficiency. Hepa1c1c7 cells transfected with control siRNA (siControl) or siULK1, were treated with vehicle (DMSO), 200nM or 500nM of SI-2 as indicated. Protein levels of NCOA3, ULK1, SREBP1, SCD1, and FASN were measured by western blot. All data represent the mean ± SEM. Data were analyzed by two-way ANOVA to assess the effects of siRNA and SI-2 treatment, followed by Tukey's post-hoc test for multiple comparisons. Actual p-values are shown. A significant treatment effect for SI-2 is present for NCOA3 ($p<0.0001$), ULK1 ($p<0.0001$), SREBP1 ($p<0.0001$), SCD1 ($p<0.0001$), and FASN ($p<0.0001$). A significant genotype effect is observed for ULK1 ($p<0.0001$), SREBP1 ($p=0.0003$), and FASN ($p=0.0003$) in siULK1 compared to siControl cells. A Genotype × treatment effect is observed following SI-2 treatment for NCOA3 ($p=0.0103$), ULK1 ($p<0.0001$), SREBP1 ($p<0.0001$), SCD1 ($p=0.001$), and FASN ($p<0.0001$).

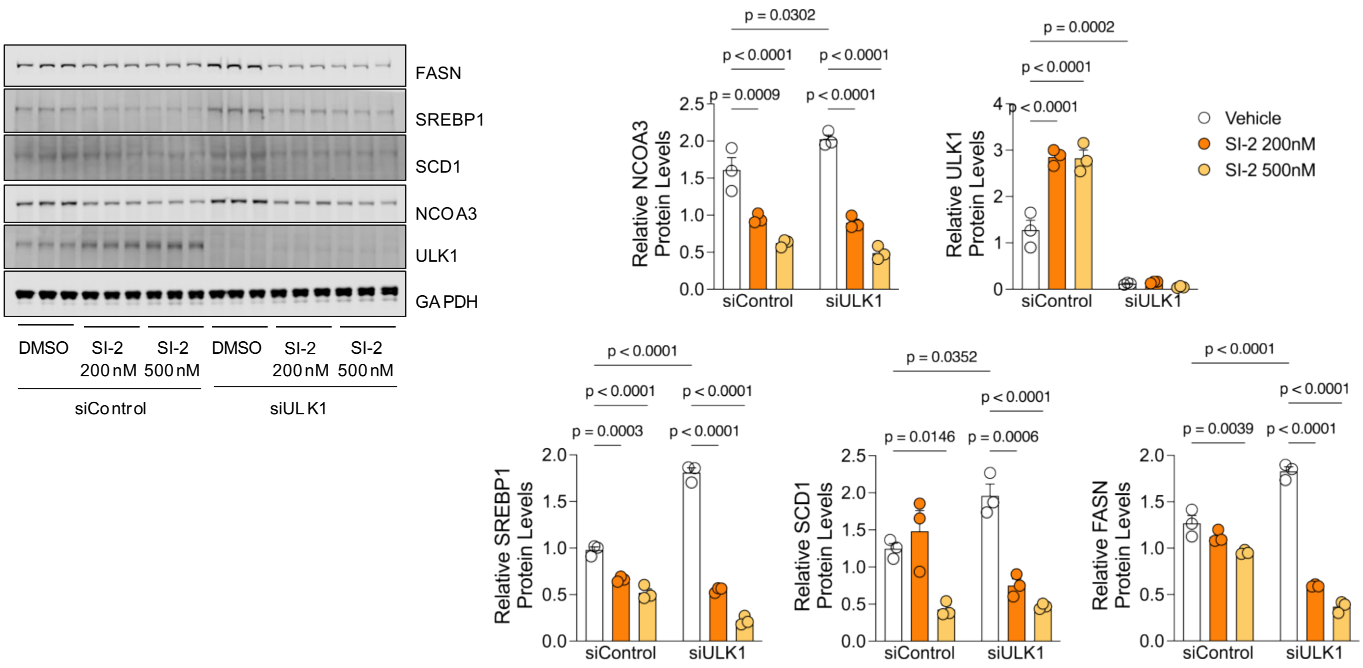

upplemental figure 12. Related to Figure 5. In cultured hepatocytes, inhibition of NCOA3 by siRNA or by e pharmacological inhibitor SI-2, blocks the induction of lipogenic genes caused by ULK1 deficiency.

Detailed Methods

Meta-analysis of ULK1 Expression and Pathway-Level Changes in Human Liver.

Publicly available human liver transcriptomic meta-analysis1(Healthy, N = 356; MASLD, N = 503) were analyzed to assess ULK1 expression and associated pathway-level changes. Raw RNA-seq data were processed to generate normalized expression values (FPKM). Group-level differences in ULK1 expression were visualized using box–whisker plots and sex-stratified violin plots. Pathway enrichment was interrogated using Gene Set Enrichment Analysis (GSEA) with Reactome, Biocarta, and Hallmark gene sets. Enrichment significance was assessed by normalized enrichment score (NES) and false discovery rate–adjusted q-value (FDR-q). To identify potential regulatory links, Pearson correlation analyses were performed between ULK1 transcript levels and transcriptional signatures of lipogenic (SREBP/SREBF), antioxidant (ARE/NRF2), and inflammatory (Hallmark) pathways. Together, these analyses highlight ULK1 as a central regulator whose downregulation in MASLD may contribute to impaired autophagy, unchecked lipid accumulation, redox imbalance, and chronic inflammation (1).

Generation of hepatocyte specific ULK1, ULK2 and NCOA3 KO mice and ULK1/2 and ULK1/NCOA3 double knockout mice

To generate hepatocyte specific-knockouts (L-ULK1, L-ULK2, L-ULK1/2, L-NCOA3, and L-ULK1/NCOA3) mice, ULK1 flox/flox, ULK2 flox/flox, ULK1/2 flox/flox, NCOA3 flox/flox and ULK1/NCOA3 flox/flox (2-5) were generated by serial matings with Albumin-Cre transgenic mice (Jackson lab, Bar Harbor, ME). NCOA3 floxed animals were obtained from cryopreserved sperm via Shanghai Model Organisms - Cat: NM-CKO-190033

Mouse Metabolic phenotyping

All animals were housed at 22-24°C, with a 12:12-h light-dark cycle and ad libitum access to standard pelleted chow or high-fat diet, (HFD; 6 0 kcal% fat, D12492; Research Diets, New Brunswick, NJ, USA) and water. 6-week-old mice were fed a HFD for 12 weeks. In a subset, diets were maintained for 60-weeks. Body weight was measured weekly, and body composition was measured by NMR (Nuclear Magnetic Resonance) with a BRUKER MINISPEC LF50 (BRUKER, Billerica, MA, USA). Metabolic cage experiments were conducted using the Comprehensive Lab Animal Monitoring System (CLAMS; Columbus Instruments, Columbus, OH, USA). Food and water intake were recorded weekly during NCD or HFD feeding. For the glucose tolerance test (GTT), glucose (1 g/kg body weight) was administered by intraperitoneal (i.p.) injection after 16 h starvation. For the insulin tolerance test (ITT), mice fasted for 6 h were injected with human insulin (1 U/kg body weight). For the pyruvate tolerance test (PTT), pyruvate (1 g/kg body weight) was administered by intraperitoneal (i.p.) injection after 16 h starvation. Serum glucose levels were determined using a Contour blood glucometer (Bayer, Healthcare LLC, Milpitas, IN, USA). Serum insulin (Crystal Chem, Elk Grove Village, IL, USA) was measured by enzyme-linked immunosorbent assays. Alanine transferase (ALT) (BioVision, Waltham, MA, USA), and aspartate aminotransferase (AST) (BioVision), Triglyceride (Cayman Chemical, Ann Arbor, MI, USA), Cholesterol (Cayman Chemical),

and Free fatty acid (CELL BIOLABS, San Diego, CA, USA) levels were determined using colorimetric assays. To pharmacologically inhibit NCOA3 in animal models, SI-2 (Millipore Sigma, CA, USA, SML1762-25MG dosed at 5 mg/kg body weight) was injected intraperitoneally twice a day for 10 days.

Oxygen consumption

Conventional Oxygen consumption of liver tissues was performed according to standard procedures as previously described (6).

Histological analysis

Tissues were fixed in 4 % formaldehyde, embedded in paraffin and sectioned. Sections were subjected to hematoxylin and eosin (H&E) staining. For Oil Red O staining, 10 μm frozen sections were rehydrated and stained by 5 % Oil Red O in 60 % isopropanol for 1 h to detect lipid droplets. Sections were washed twice with distilled water prior to microscopy.

Transmission Electron Microscopy

Electron microscopy fixation and quantification techniques were based on established protocols meant to minimize artifacts(7, 8). Briefly, liver tissues and cells were fixed in 2.5% glutaraldehyde in sodium cacodylate buffer at 37C for 1 hour, then postfixed in buffered 1% osmium tetroxide, stained with 2% uranyl acetate, dehydrated with an ethanol graded series, and embedded in Embed-812 resin. Thin sections were cut on an ultramicrotome and stained with 2% uranyl acetate and lead citrate. Images were acquired on the JEOL JEM 1230 electron microscope operating at 120 kV. Measurements of the lipid number, mitochondrial area, circularity and number, lysosome number and area, autophagosome number, and area were made with the Multi Measure ROI tool of Fiji ImageJ, as described (9).

Immunogold Labeling

Conventional immunogold labeling on ultrathin cryosections of liver tissues was performed according to standard procedures as previously described (10-12).

Cell Culture and Transfection

Hepa1c1c7 cells were maintained in α-MEM supplemented with 10% FBS (Invitrogen, Waltham, Massachusetts, USA). Transfection of plasmids expressing ULK1-myc and NCOA3 was performed using Lipo2000 (Thermo Fisher Scientific, Cat# 11668027, Waltham, MA, USA). Small interfering RNAs (siRNAs) of nonspecific RNAs (Horizon Discovery, Cat# D-001810-10-05), ULK1 (Horizon Discovery, Cat# L-040155-00-0005), ATG3 (Horizon Discovery, Cat# L-048439-00-0005), ATG5 (Horizon Discovery, Cat# L-064838-00-0005), ATG7 (Horizon Discovery, Cat# L-049953-00-0005), NCOA3 (Horizon Discovery, Cat# L-047722-02-0005), and CREB (Horizon Discovery, Cat# L-040959-01-0010) were purchased from Horizon Discovery (Waterbeach, Cambridge, UK). The siRNAs (50 nmol/l) were mixed with Lipofectamine™ RNAiMAX (Invitrogen, Cat# 13778150) in 200 μL of serum-free α-MEM and incubated for 15 min at room temperature. These reagents were added to cells in 800 μL of serum-free α-MEM. After 4 h incubation, 1 ml of α-MEM supplemented with 10 % FBS was added.

Hepatic triglyceride secretion

WT and L-ULK1 KO mice were injected with a 10% poloxamer 407 solution (1 mg/g; Sigma-Aldrich, St. Louis, MO, USA) i.p. after 4 hours of fasting. Triglyceride levels were measured before and 1, 6, and 24 h after the injection.

De Novo Lipogenesis Assay

De novo lipogenesis was measured using D_2O labeling as previously described (13), with minor modifications. Briefly, **four-week-old mice** received an intraperitoneal injection of D_2O in saline (33 μl/g body weight, 0.9% saline) between 16:00–17:00 and were subsequently provided with 5% D_2O in drinking water throughout the study to maintain body water enrichment. Tail blood was collected at 19:00 and at 10:00 the following day to assess serum enrichment. At the terminal time point (+1 day), mice were euthanized, and liver tissues were rapidly clamped in liquid nitrogen and stored at –80 °C. For metabolite extraction, frozen liver samples were pulverized in a Cryomill (Retsch) and homogenized in methanol:acetonitrile:water (40:40:20) with 0.5% formic acid, followed by neutralization with ammonium bicarbonate. Serum samples were extracted with 100% methanol. Saponified fatty acids were prepared by alkaline hydrolysis in methanol/KOH, followed by hexane extraction and resuspension in methanol:acetonitrile. Samples were analyzed by LC–MS (Orbitrap Exploris 480, Thermo Fisher Scientific) using HILIC columns for polar metabolites and C18 columns for fatty acids. Raw spectra were converted to mzXML and processed in EIMAVEN (v0.12.0) for isotopologue annotation and natural abundance correction. D_2O body water enrichment was determined from serum measurements, and flux of newly synthesized palmitate in liver was calculated using the kinetic model described by Zhang et al (13).

Real time qPCR

Total RNAs were isolated using TRIzol (Invitrogen) according to the manufacturer's instruction. Real-time qPCR was performed (in duplicate) using the SYBR-master mix (Applied Biosystems, Woolston Warrington, UK) and the ABI 7400 Real-time PCR system (Applied Biosystem, Foster City, CA, USA). Each cycle threshold (Ct) value was subtracted from Ct value of GAPDH (△Ct), and then subtracted from the value of each control set (△△Ct). Relative mRNA levels were expressed as 2-ΔΔCt. Primer sequences are listed in Supplemental Table.

Plasmids and antibodies

ULK1-myc plasmid (Cat# 31960) and myc-ULK1 M92A (Cat# 31962) were purchased from Addgene (Watertown, Massachusetts, USA,). NCOA3 plasmid (Genomics-online, Cat# ABIN380975) was purchased from Genomics-online (Limerick, PA, USA). NCOA3 mutants were generated by site directed mutagenesis reaction with serine substitution to alanine. The mutagenesis reaction involves PCR amplification of the template DNA using the designed mutagenic primers. During PCR, the primers hybridize with the template

DNA, and DNA polymerase extends the primers to generate a mutated DNA strand. The resulting PCR product contains the desired mutations incorporated into the target sequence. Antibodies against ULK1 (CST, Cat# 8054S), GAPDH (CST, Cat# 5174S), ATG3 (CST, Cat# 3415S), ATG5 (CST, Cat# 12994S), ATG7 (CST, Cat# 8558S), ATG13 (CST, Cat# 13468S), Phospho-Akt (Ser473) (CST, Cat# 4060S), AKT (CST, Cat# 4685S), SCD1 (CST, Cat# 2794S), SQSTM1/p62 (CST, Cat# 23214S), NCOA3 (CST, Cat# 2126S), CREB (CST, Cat# 9197S), NRF2 (CST, Cat# 12721S), and KEAP1 (CST, Cat# 7705S) were purchased from Cell Signaling Technology (Danvers, Massachusetts , USA). Antibodies against FASN (Cat# sc-48357), SREBP1 (Cat# sc-13551), and MTP (Cat# sc-515742) were purchased from Santa Cruz Biotechnology (Dallas, Texas, USA). Antibodies against LC3B (Cat#SAB5700240) was obtained from Sigma-Aldrich (St. Louis, MO, USA).

Luciferase reporter assay

Hepa1c1c7 cells (ATCC Virgina, USA, Cat: CRL-2026) were cultured in α-MEM supplemented with 10 % FBS. Hepa1c1c7 cells were seeded in 12-well plates and transfected with reporter vectors (300 ng) of CRE/CREB Reporter Kit (BPS Bioscience, Cat#6061, San Diego, California, USA) and pRSV-β-galactosidase (100 ng) using Lipo2000 (Invitrogen, Cat# 11668019). And then siRNA of ULK1 and NCOA3 were transfected with RNAiMAX (Invitrogen, Cat# 13778150) the following day. After 24h, luciferase and β-galactosidase activities were measured according to the manufacturer's instruction (Promega, Madison, WI).

Immunoprecipitation and Western blot analysis

For immunoprecipitation, Hepa1c1c7 cells plated on 6-well plates were transfected with expression vectors for ULK1-myc (300ng) and NCOA3 (300 ng) in combination, using Lipofectamine 2000 (Invitrogen, Cat# 11668019). After incubation for 24 h, cells were lysed in the RIPA buffer (Sigma-Aldrich, Cat# R0278) supplemented with 1X protease inhibitors (Thermo Fisher Scientific, Cat# 78438). Cell lysates (0.5 mg) were incubated with anti-c-myc antibody-coupled agarose beads (Thermo Fisher Scientific, Cat# 20168) for 16h at 4 °C. The precipitates were washed five times with PBS and subjected to SDS–polyacrylamide gel electrophoresis. For western blot analysis of tissue or cell lysates, samples were prepared in RIPA buffer supplemented with protease inhibitors. Proteins resolved on SDS–polyacrylamide gels were transferred onto a nitrocellulose membrane (GE Healthcare, Chicago, IL, USA). The bands were visualized by ODYSSEY CLx (LI-COR, Lincoln, NE, USA).

In vitro kinase assay

ULK1-mediated phosphorylation of NCOA3 was evaluated using the ULK1 kinase assay kit (Promega) coupled with the ADP-Glo Kinase Detection System (Promega). Myelin Basic Protein (MBP), a known ULK1 substrate, was used as a positive control. Recombinant NCOA3 fragments (amino acids 520–710 and 820–950) were tested as potential ULK1 substrates, with a substrate-free reaction serving as a negative control to monitor ULK1 autophosphorylation. Each reaction contained 0.05 μg of ULK1 and 1 μg of substrate protein (MBP or NCOA3 fragment) in 1× kinase reaction buffer supplemented with 50 μM ATP and 50 μM DTT, in a total volume of 25 μL. Reactions were incubated at room temperature for 60 min. ADP generated during the kinase reaction was converted

to ATP by adding ADP-Glo reagent, followed by luminescence detection using the kinase detection reagent. Luminescence was recorded over 60 min to monitor reaction kinetics. The assay principle is based on the proportional relationship between ATP consumption during phosphorylation and the amount of luminescence generated by ADP-to-ATP conversion.

Reactive Oxygen Species (ROS) Detection

ROS in mouse liver tissue (50 mg) was measured using the DCF ROS/RNS Assay Kit (Abcam, Cat# ab238535, Cambridge, United Kingdom). The experiments were performed according to the manufacturer's protocol.

Sample Preparation for phospho-Proteomics Analyses

Hepa1c1c7 cells were transfected with siControl (50 nM) and siULK1 (50 nM) (n=4 per condition). Cells were washed three times with ice-cold cold PBS, then lysed with (lysis buffer components). Lysates were reduced with 5 mM DTT at 56 ºC for 30 min, alkylated with 15 mM iodoacetamide at room temperature for 45 min, then subjected to LysC (Wako, Saitama Prefecture, Japan) digestion at a 1:50 enzyme:protein ratio at 37ºC for 2 h. Samples were diluted in1M urea, then digested overnight with trypsin (Promega, Madison, Wisconsin, USA) at a 1:50 enzyme:protein ratio at 37ºC. The resulting peptide samples were acidified and desalted using StrataX 33 µm Polymeric Reversed Phase, 10 mg cartridges (Phenomenex, Torrance, California, USA) and eluates were dried via vacuum centrifugation. Peptide concentration was determined using Pierce Quantitative Colorimetric Peptide Assay. 3.0 µg of each sample was reconstituted with 200 mM TEAB, then individually labeled with a TMT 10-plex reagent (Thermo Fisher) for 2 hr. at room temperature. Samples were quenched with 50% hydroxylamine to a final concentration of 0.4%. Labeled peptide samples were mixed 1:1 ratio (8 samples total), dried via vacuum centrifugation, then desalted using SepPak C18 SPE cartridges (Waters, 500mg sorbent, Milford, MA, USA). 100 µg of the mixed TMT sample was fractionated into six 'global proteome' fractions using high pH reverse phase spin columns (Pierce, Pierce City, MO, USA). The remaining TMT sample was enriched for phosphopeptides using MagReSyn Ti-MAC beads (ReSyn Biosciences, Edenvale, Gauteng, South Africa), as previously described (14). The phosphopeptide-enriched samples were fractionated into three 'phosphoproteome' fractions using high pH reverse phase spin columns (Pierce). The proteome and phosphoproteome fractions were dried via vacuum centrifugation and stored at -80°C until further analysis. For mouse liver, 200 µg protein was lysed in 8 M urea, boiled (95 °C, 5 min), sonicated, and quantified (BCA assay). Proteins were reduced/alkylated with TCEP (10 mM) and 2-chloroacetamide (40 mM, 23 °C, 30 min). Urea was diluted 8-fold with 100 mM Tris-HCl (pH 8), and proteins digested overnight (trypsin/LysC, 1:100, 33 °C). Peptides were acidified (TFA, pH 2–3) and desalted (HLB, 30 mg). ~10% was retained for global proteomics, and 90% was enriched for phosphopeptides using Ti-IMAC beads (1:2 peptide:beads, binding buffer 0.1 M glycolic acid in 80% ACN, 5% TFA). Beads were washed sequentially (binding buffer, wash buffer 1, wash buffer 2, water) and phosphopeptides eluted with 1% NH_4OH, acidified, pooled, dried, and resuspended in 0.1% formic acid. 500 ng peptides were analyzed using a timsTOF HT mass spectrometer (Bruker Daltonics, Billerica, MA, USA).

Mass spectrometry proteomics acquisition

Dried peptides were resuspended in 0.1% (v/v) FA in MS grade water (Fisher) and analyzed on a timsTOF HT mass spectrometer, paired with a Vanquish Neo UHPLC system. Mobile phase A consisted of 0.1% (v/v) FA in MS grade water (Fisher), and mobile phase B consisted of 0.1% (v/v) FA in 100% MS grade Acetonitrile (Fisher). The LC was operated in trap-and-elute mode, where the peptides were first trapped onto a PepMap Neo Trap column (5 mm, 100 Å pore size, 5 µm particle size) and then reversed-phase separated using gradients mentioned below on an Aurora Elite C18 reverse phase column (15 cm, 100 Å pore size, 1.5 µm particle size for captive spray, IonOptiks), kept at 50°C using a column oven for Bruker Captive Spray source (Sonation Lab Solutions, Biberach, Germany), and ionized in a CaptiveSpray source (Bruker Daltonics) at 1700 V. For global proteome analysis, the %B gradient used was: 5% to 35% over 37 min at 0.3 µL/min, then to 45% in the next 4 mins, and 60% in the next 1 min, followed by an increase to 95% B over 3 min. For phosphoproteome analysis, the %B gradient used was 3% to 16% over 26 min, 30% in the next 11.5 min, 45% in the next 4 min, and 60% B over 1 min at 0.3 µL/min flow rate. For phosphoproteome analysis, the raw data was acquired in dia-PASEF mode with variable isolation window widths in the m/z vs ion mobility plane. These windows were adjusted to maximize the coverage of precursor ions. For dia-PASEF, in the ion mobility (1/K0) range 0.6 to 1.60 Vs cm-2, the collision energy was linearly decreased from 59 eV at 1/K0 = 1.28 Vs cm-2 to 20 eV at 1/K0 = 0.62 Vs cm-2 to collect the MS/MS spectra in the mass range 256.9 to 1175.9 Da. The estimated mean cycle time was 1.27 s. For the global abundance data analysis, equal-size windows of 21 Da were designed with an overlap of 1 Da to maximize the precursors ion coverage for further MS/MS. The ion accumulation time and ramp times in the dual TIMS analyzer were set to 100 ms each. In the ion mobility (1/K0) range 0.6 to 1.6 Vs cm-2, the collision energy was linearly decreased from 59 eV at 1/K0 = 1.32 Vs cm-2 to 20 eV at 1/K0 = 0.65 Vs cm-2 to collect the MS/MS spectra in the mass range 266.3 to 1212.3 Da. The estimated mean cycle time was 1.48 s

Data Analysis of phospho-Proteomics

For in vitro data, raw files were processed in MaxQuant v1.6.1.0 with ‘reporter ion MS2’ and ‘10plex TMT’. Isolation purity was >0.7. Peak lists were searched against a reviewed UniProt *Mus musculus* database (May 2019, 17,457 sequences) plus common contaminants using Andromeda. Parameters included up to two missed trypsin cleavages, fixed carbamidomethylation (C), and variable M oxidation and S/T/Y phosphorylation. Peptide FDR was set at 1%, and only phosphopeptides with localization probability >0.7 were reported. Data visualization and analysis were performed in Perseus, Excel, and R. For mouse liver, raw files were analyzed in Spectronaut (Biognosys) using the directDIA+ algorithm. Fixed modification was carbamidomethylation (C), and variable modifications included protein N-term acetylation, methionine oxidation, and S/T/Y phosphorylation (for phosphoproteomics). A reviewed human UniProt database (Oct 2023) was used for spectral matching. FDR was 1% for PSM, peptide, and protein levels, with minimum PTM localization threshold set to 0. MS2-level area-based quantification was performed without cross-run normalization (later normalized using MSstats). Quantitative analyses were conducted in R v4.4.1. QC included inter-run clustering, correlations, PCA, and peptide/protein counts and intensities. Statistical analysis of protein and phosphosite abundance changes between conditions was performed using MSstats v4.12.1. For

protein abundance, peptides mapping to the same protein were summarized via Tukey's Median Polish; for phosphoproteomics, peptides with the same phosphorylated sites were summarized into site groups. MSstats normalized by median equalization, with imputation off. Log2 fold changes were calculated as the ratio of averaged intensities between conditions, p-values via Student's t-test, and FDR-adjusted using Benjamini-Hochberg.

Histopathological assessment of MAFLD

The liver sections were fixed in formalin, embedded and then stained with H&E and Masson Trichrome. A board-certified pathologist evaluated H&E and Trichrome slides for the severity of fatty liver disease in mice, blinded to the experimental groups. The NASH clinical research network scoring system (NAS) was applied for semiquantitative scoring (15).

mRNA sequencing and data analysis

Total RNA was obtained from liver tissues of 4-week-old control or L-ULK1 KO mice. Transcript profiling using RNA-Seq was performed by the University of Iowa Genomics Division using manufacturer recommended protocols. Briefly, 500 ng of DNase I-treated total RNA was used to prepare sequencing libraries using the Illumina TruSeq stranded mRNA library preparation kit (Cat. #RS-122-2101, Illumina, Inc., San Diego, CA, USA). The molar concentrations of the resulting indexed libraries were measured using the 2100 Agilent Bioanalyzer (Agilent Technologies, Santa Clara, CA, USA) and combined equally into pools for sequencing. The concentrations of the library pools were measured using the Illumina Library Quantification Kit (KAPA Biosystems, Wilmington, MA, USA) and sequenced on the Illumina NovaSeq 6000 genome sequencer using 150 bp paired end SBS chemistry. Paired-end reads were quantified using Kallisto (v0.46.2) and differential expression analysis performed with DESeq2 (v1.26.0). Pathway analysis and gene ontology was performed using Qiagen's Ingenuity Pathway Analysis and Advaita's iPathwayGuide (16, 17).

References

1. Li Z, Zhang H, Li Q, Feng W, Jia X, Zhou R, et al. GepLiver: an integrative liver expression atlas spanning developmental stages and liver disease phases. *Sci Data.* 2023;10(1):376.
2. Kundu M, Lindsten T, Yang CY, Wu J, Zhao F, Zhang J, et al. Ulk1 plays a critical role in the autophagic clearance of mitochondria and ribosomes during reticulocyte maturation. *Blood.* 2008;112(4):1493-502.
3. Cheong H, Wu J, Gonzales LK, Guttentag SH, Thompson CB, and Lindsten T. Analysis of a lung defect in autophagy-deficient mouse strains. *Autophagy.* 2014;10(1):45-56.
4. Cheong H, Lindsten T, Wu J, Lu C, and Thompson CB. Ammonia-induced autophagy is independent of ULK1/ULK2 kinases. *Proc Natl Acad Sci U S A.* 2011;108(27):11121-6.

5. Xie Y, Yuan Q, Cao X, Qiu Y, Zeng J, Cao Y, et al. Deficiency of Nuclear Receptor Coactivator 3 Aggravates Diabetic Kidney Disease by Impairing Podocyte Autophagy. *Adv Sci (Weinh).* 2024:e2308378.
6. Osto C, Benador IY, Ngo J, Liesa M, Stiles L, Acin-Perez R, et al. Measuring Mitochondrial Respiration in Previously Frozen Biological Samples. *Curr Protoc Cell Biol.* 2020;89(1):e116.
7. Hinton A, Jr., Katti P, Christensen TA, Mungai M, Shao J, Zhang L, et al. A Comprehensive Approach to Sample Preparation for Electron Microscopy and the Assessment of Mitochondrial Morphology in Tissue and Cultured Cells. *Adv Biol (Weinh).* 2023;7(10):e2200202.
8. Neikirk K, Lopez EG, Marshall AG, Alghanem A, Krystofiak E, Kula B, et al. Call to action to properly utilize electron microscopy to measure organelles to monitor disease. *Eur J Cell Biol.* 2023;102(4):151365.
9. Lam J, Katti P, Biete M, Mungai M, AshShareef S, Neikirk K, et al. A Universal Approach to Analyzing Transmission Electron Microscopy with ImageJ. *Cells.* 2021;10(9).
10. Giordano F, Saheki Y, Idevall-Hagren O, Colombo SF, Pirruccello M, Milosevic I, et al. PI(4,5)P(2)-dependent and Ca(2+)-regulated ER-PM interactions mediated by the extended synaptotagmins. *Cell.* 2013;153(7):1494-509.
11. Galmes R, Houcine A, van Vliet AR, Agostinis P, Jackson CL, and Giordano F. ORP5/ORP8 localize to endoplasmic reticulum-mitochondria contacts and are involved in mitochondrial function. *EMBO Rep.* 2016;17(6):800-10.
12. Vue Z, Garza-Lopez E, Neikirk K, Katti P, Vang L, Beasley H, et al. 3D reconstruction of murine mitochondria reveals changes in structure during aging linked to the MICOS complex. *Aging Cell.* 2023;22(12):e14009.
13. Zhang Z, TeSlaa T, Xu X, Zeng X, Yang L, Xing G, et al. Serine catabolism generates liver NADPH and supports hepatic lipogenesis. *Nat Metab.* 2021;3(12):1608-20.
14. Esnault S, Hebert AS, Jarjour NN, Coon JJ, and Mosher DF. Proteomic and Phosphoproteomic Changes Induced by Prolonged Activation of Human Eosinophils with IL-3. *J Proteome Res.* 2018;17(6):2102-11.
15. Kleiner DE, Brunt EM, Van Natta M, Behling C, Contos MJ, Cummings OW, et al. Design and validation of a histological scoring system for nonalcoholic fatty liver disease. *Hepatology.* 2005;41(6):1313-21.
16. Bray NL, Pimentel H, Melsted P, and Pachter L. Near-optimal probabilistic RNA-seq quantification. *Nat Biotechnol.* 2016;34(5):525-7.
17. Love MI, Huber W, and Anders S. Moderated estimation of fold change and dispersion for RNA-seq data with DESeq2. *Genome Biol.* 2014;15(12):550.

Table 1. Primers

qRT-PCR Primers		
Genes	Forward Primer 5' - 3'	Reverse Primer 5' - 3'
Human ULK1	cggtggtcttcaccgtgggc	caccaggtggcgggcagaag
Human GAPDH	gccttccgtgtccccactgc	cctccgacgcctgcttcacc
Mouse ULK1	ctccagagcaacatgatggc	cttccctgtcagacactggt
Mouse ULK2	tgtccaaagactctgcgagt	acactgccctccacacataa
Mouse GAPDH	ttcctacccccaatgtgtcc	agacaacctggtcctcagtg
Mouse SREBP1	aacctcatccgccacctgct	cactggcacgggcatccttc
Mouse SCD1	ggcagccgataaaagggggc	actgcgcttggaaacctgcc
Mouse FASN	agcactgccctgcagaagga	caagcagctgccaggagtcg
Mouse IL-4	ggagaagggacgccatgcac	tgcgaagcaccttggaagcc
Mouse IL-6	acaaccacggccttccctact	agcctccgacttgtgaagtggt
Mouse TNFα	ggacaaggctgccccgacta	ccctcaggggtgtccttggg
Mouse Interferon γ	tcttcagcaacagcaaggcgaa	cagctggtggaccactcgga
Mouse CSF1	ccgcagagctggaaggagga	caggaggcccccaacagtca
Mouse Keratin19	gaaggcacgctggcagagac	aggtggcgatctcctgctcc
Mouse CCL2	taacgccccactcacctgct	tggggtcagcacagacctctc
Mouse CCL3	tggagctgacaccccgactg	tgacacctggctgggagcaa
Mouse CCL4	gaagctctgcgtgtctgccc	gcttctgtgaagctgccggg
Mouse CCL5	tctgcagctgccctcaccat	cgcgagggagaggtaggcaa
Mouse PEPCK	atccagggcagcctcgacag	tgcggatgacaccctcctcc
Mouse G6Pase	agccaggaccttccaactggt	agggagagacagtgcgagcc
