# Supplementary material for: The ULK1-NCOA3 axis restrains de novo lipogenesis and prevents diet-induced steatohepatitis and fibrosis in mice
Source: J Clin Invest. 2026 Apr 2;136(11):e191101. doi: 10.1172/JCI191101 (PMC13221236; doi:10.1172/JCI191101)

Figure 1. Human and murine MASLD associate with reduced ULK1 expression.

(C)

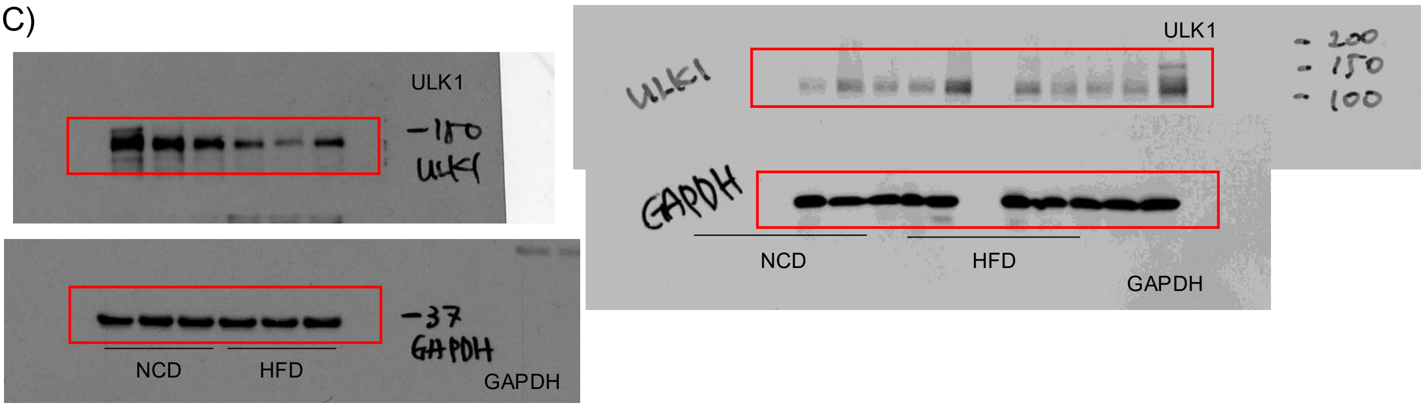

(E)

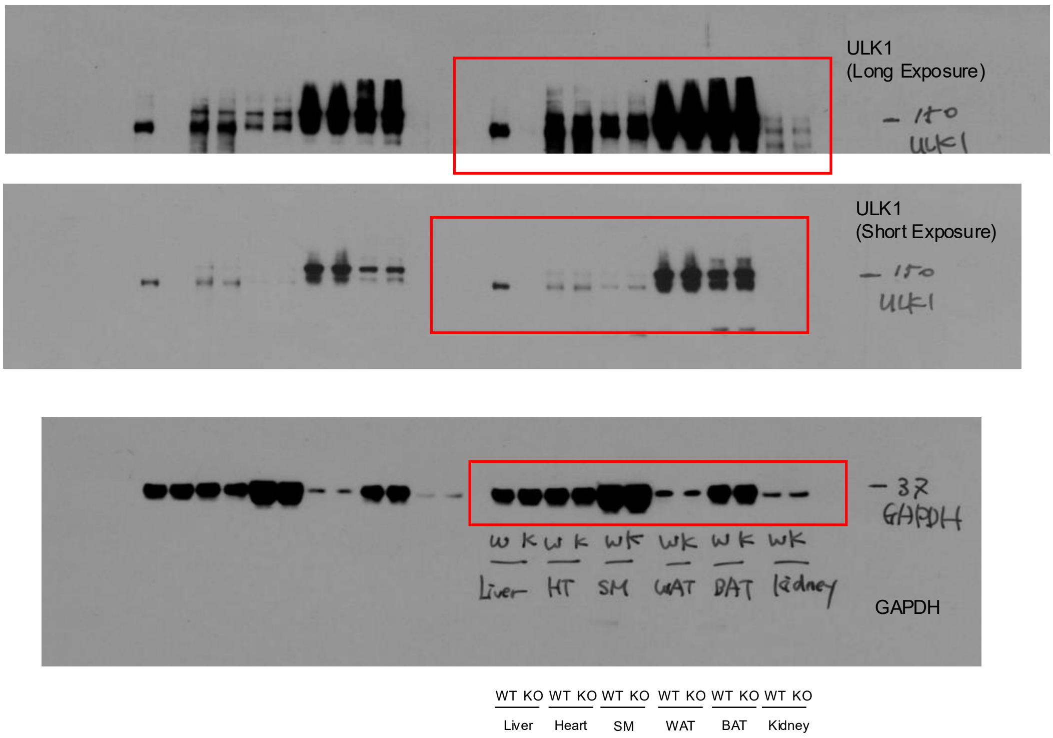

(F)

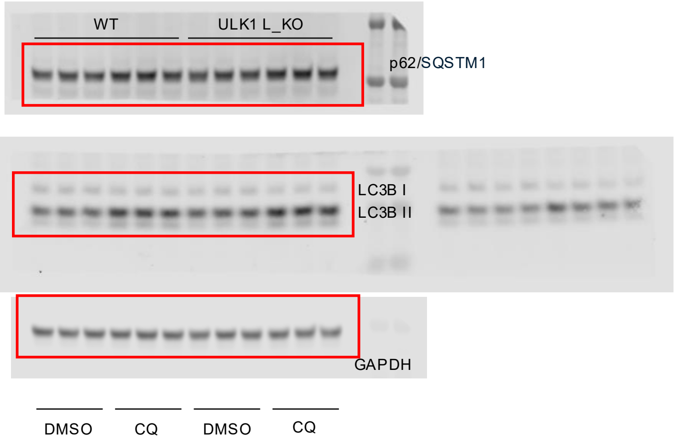

Figure 3. Hepatic ULK1 deficiency drives lipogenesis, independent of insulin resistance.

(B)

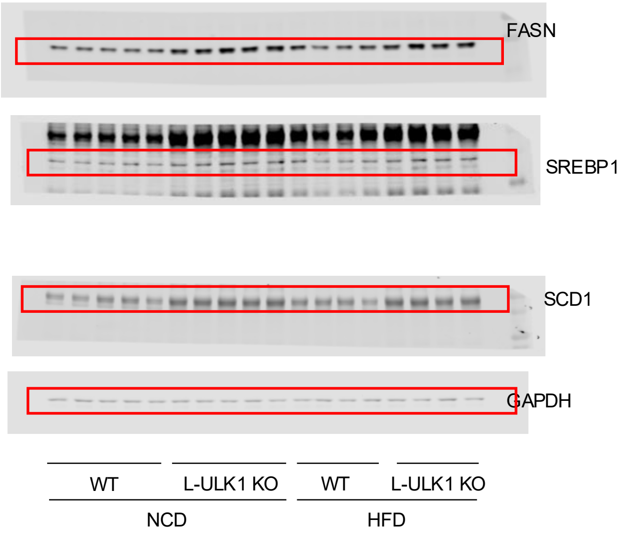

(C)

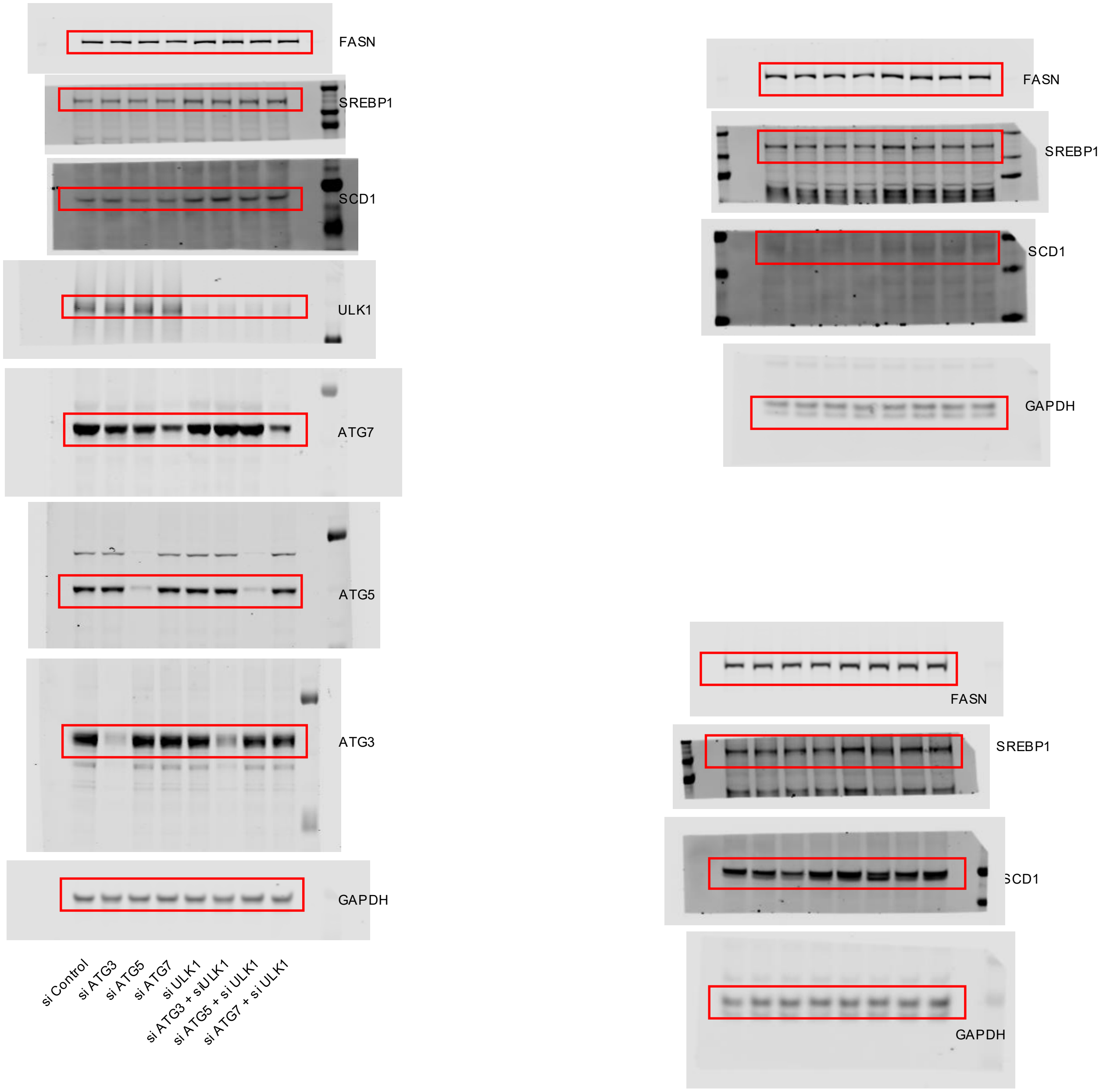

Figure 3. Hepatic ULK1 deficiency drives lipogenesis, independent of insulin resistance.

(E)

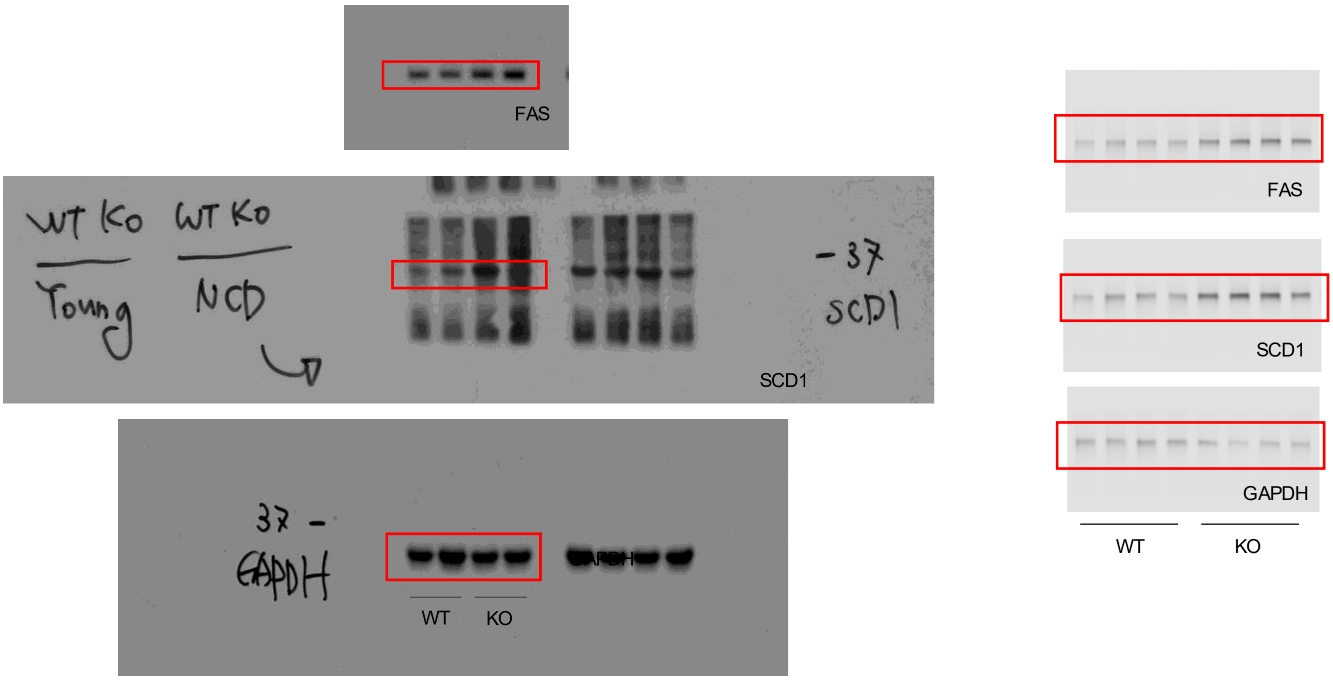

(F)

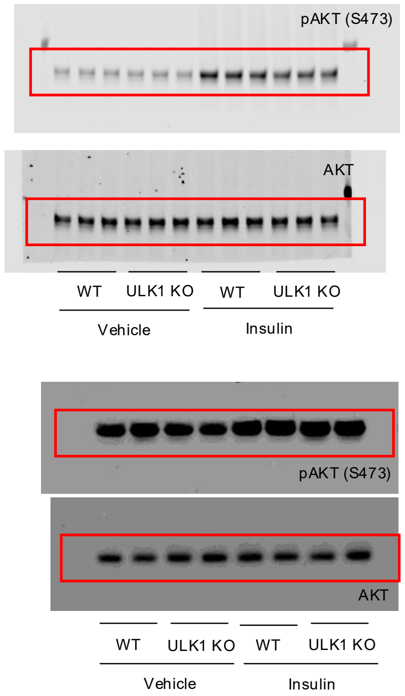

Figure 4. Phosphorylation of NCOA3 by ULK1 blocks lipogenesis by modulating transcriptional activities of the CREB-CBP complex.

(B)

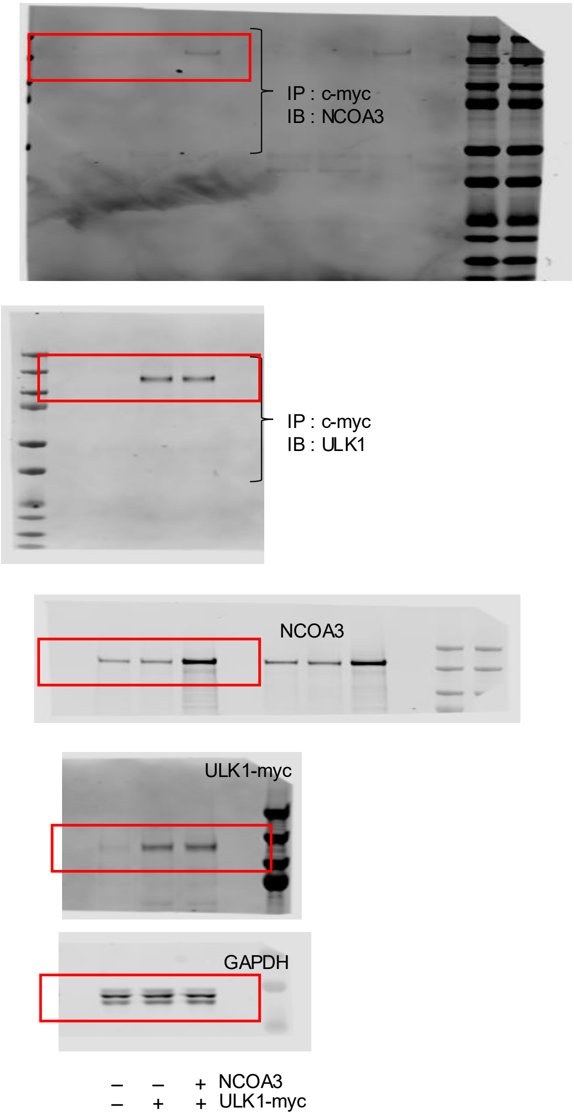

(E)

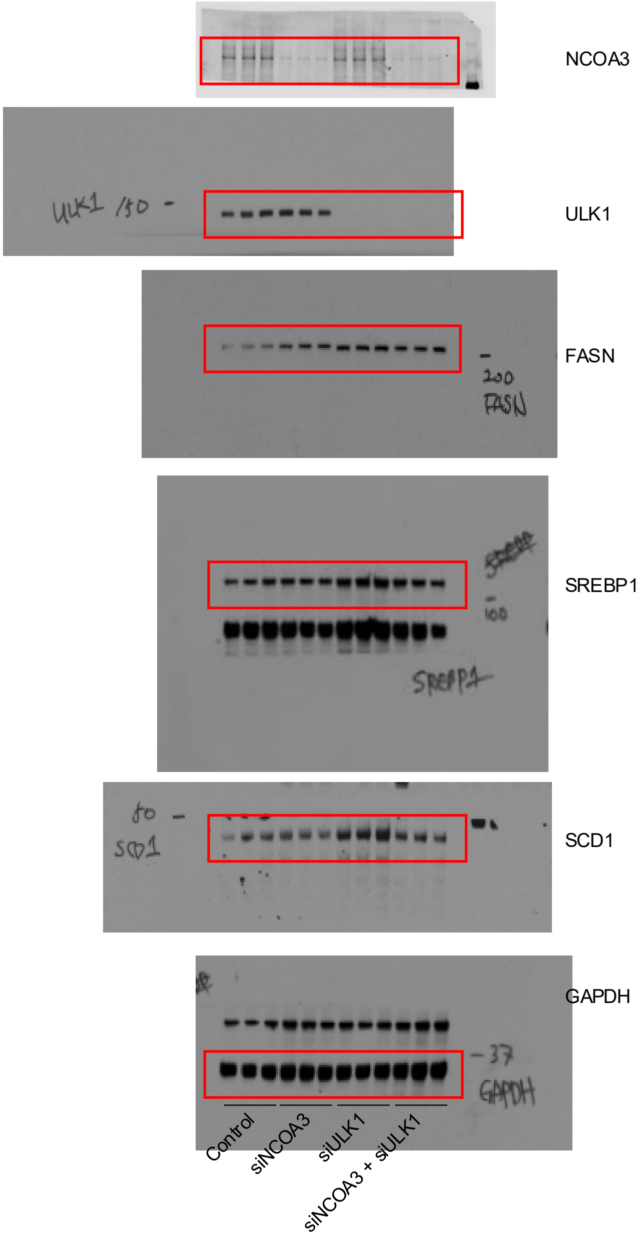

Figure 4. Phosphorylation of NCOA3 by ULK1 blocks lipogenesis by modulating transcriptional activities of the CREB-CBP complex.

(F)

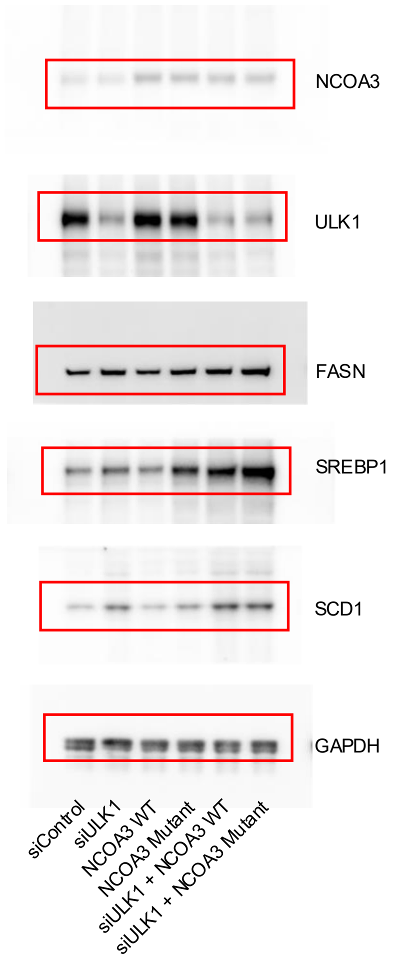

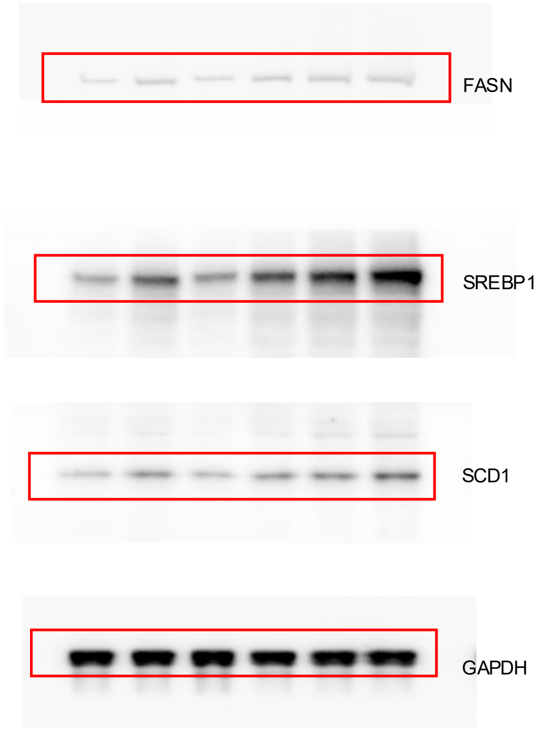

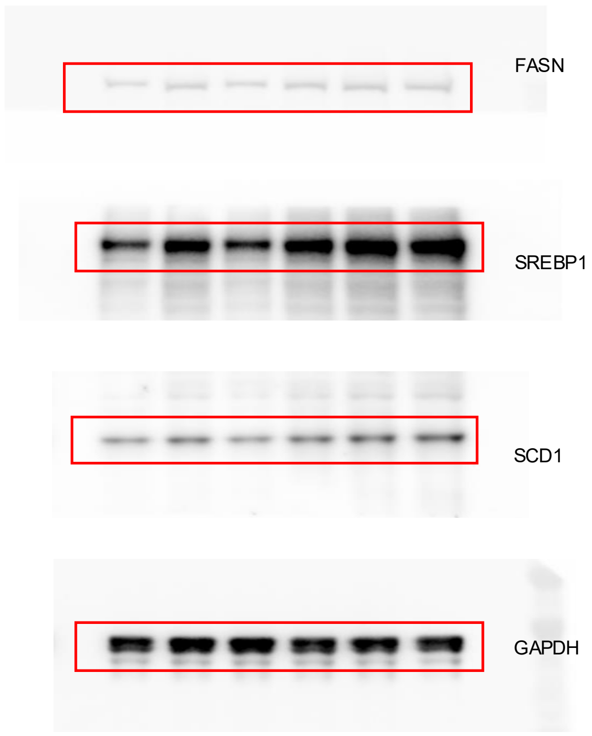

Figure 4. Phosphorylation of NCOA3 by ULK1 blocks lipogenesis by modulating transcriptional activities of the CREB-CBP complex.

(G)

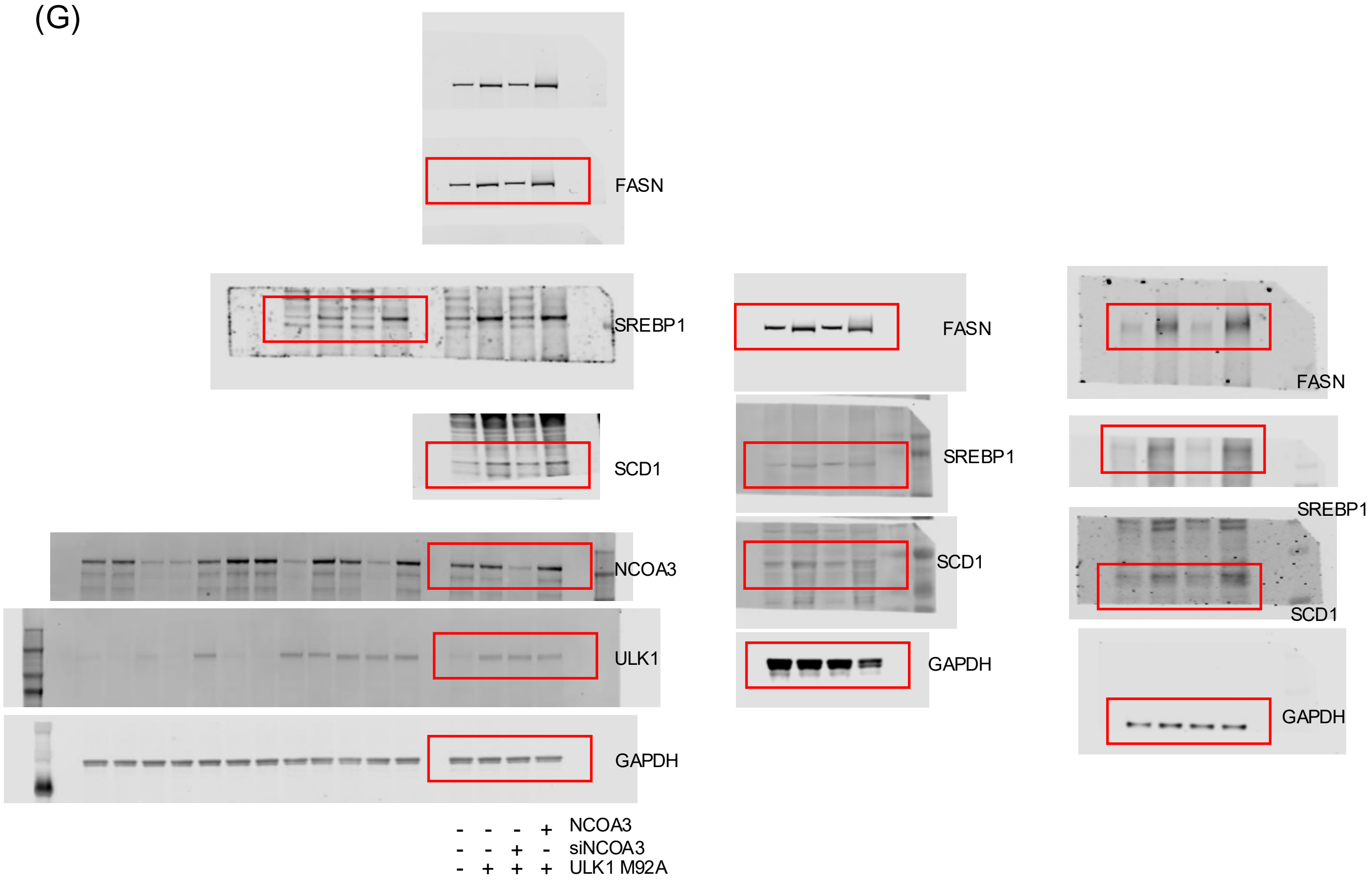

Figure 4. Phosphorylation of NCOA3 by ULK1 blocks lipogenesis by modulating transcriptional activities of the CREB-CBP complex.

(J)

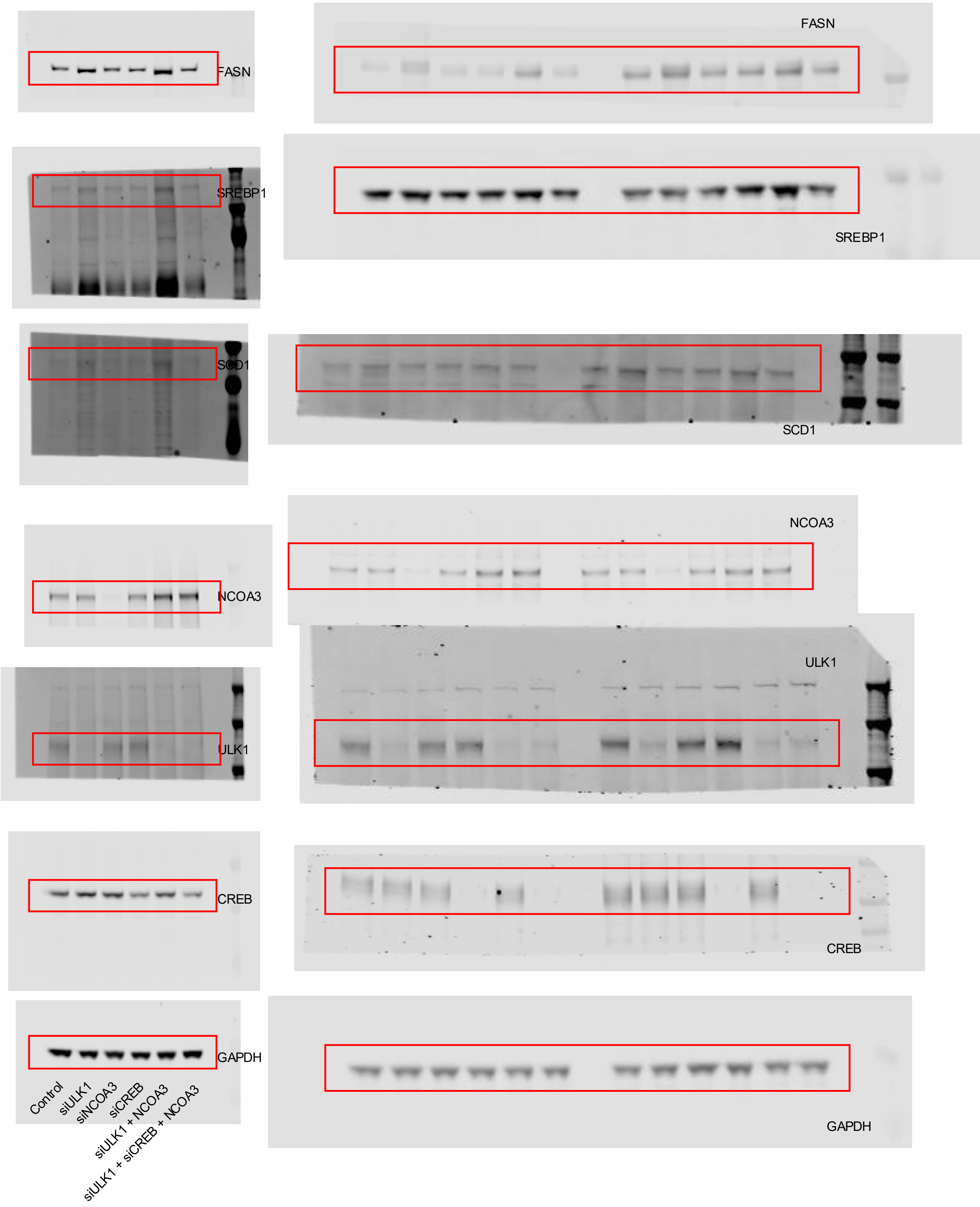

Figure 5. Hepatocyte-specific loss of NCOA3 prevents MASLD due to ULK1 deficiency.

(D)

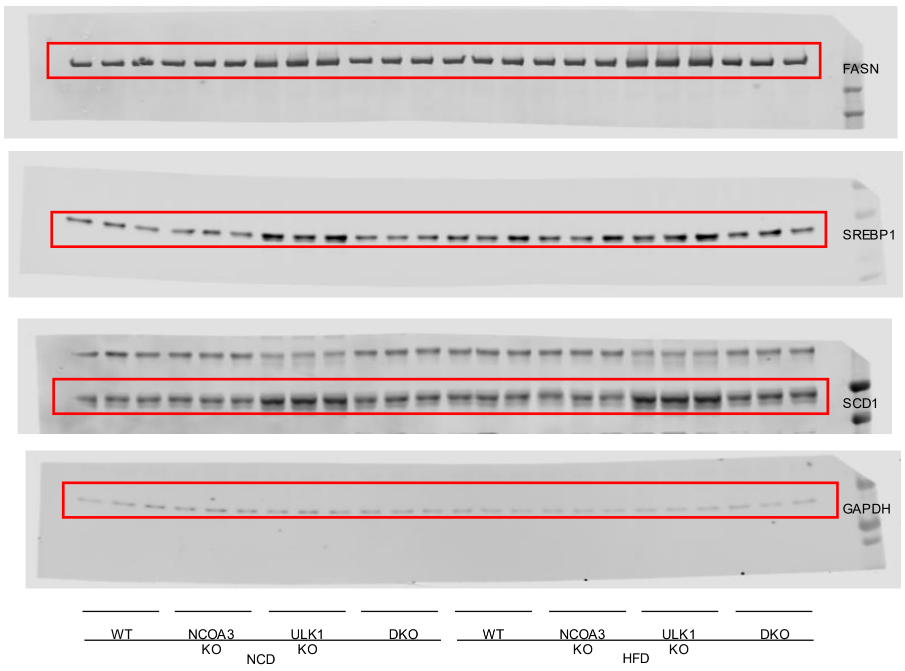

(H)

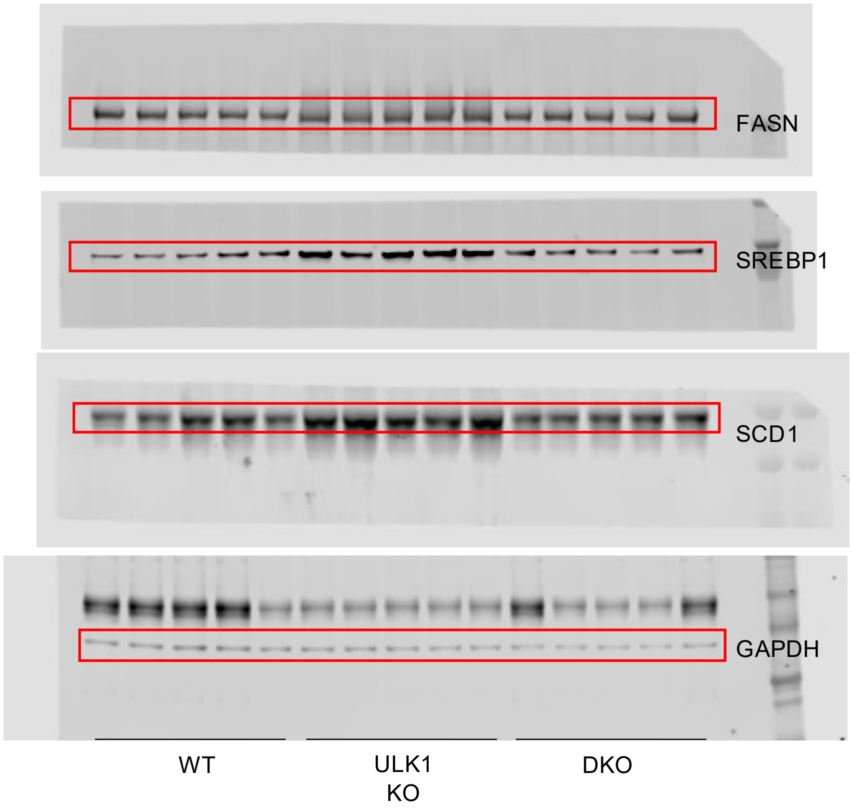

Figure 5. Hepatocyte-specific loss of NCOA3 prevents MASLD due to ULK1 deficiency.

(I)

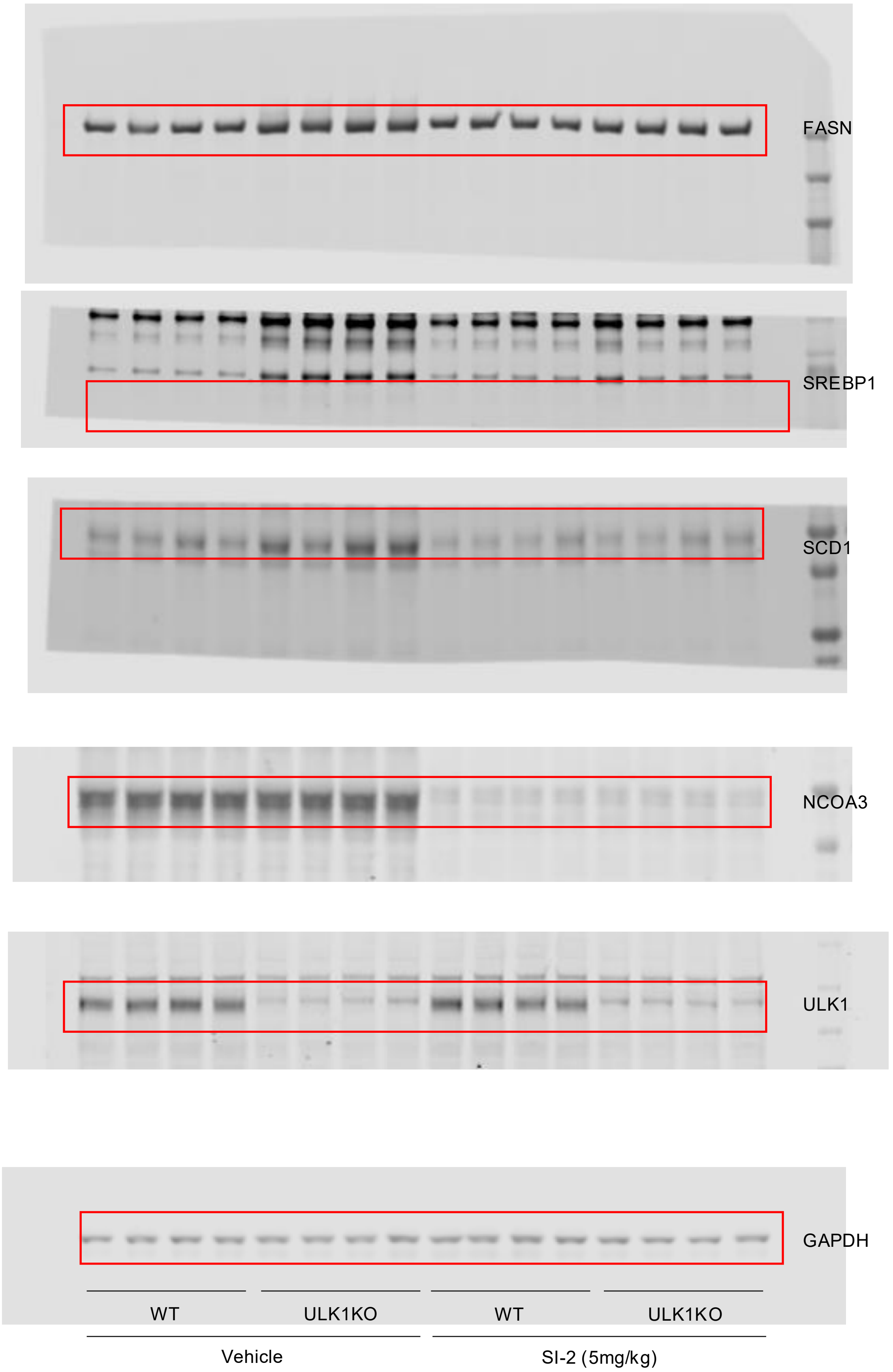

Figure 6. Hepatic ULK1 deficiency in mice induces fibrosis, and in humans with MASLD associates with induction of lipogenic and inflammatory genes and repression of NRF2 signaling

(D)

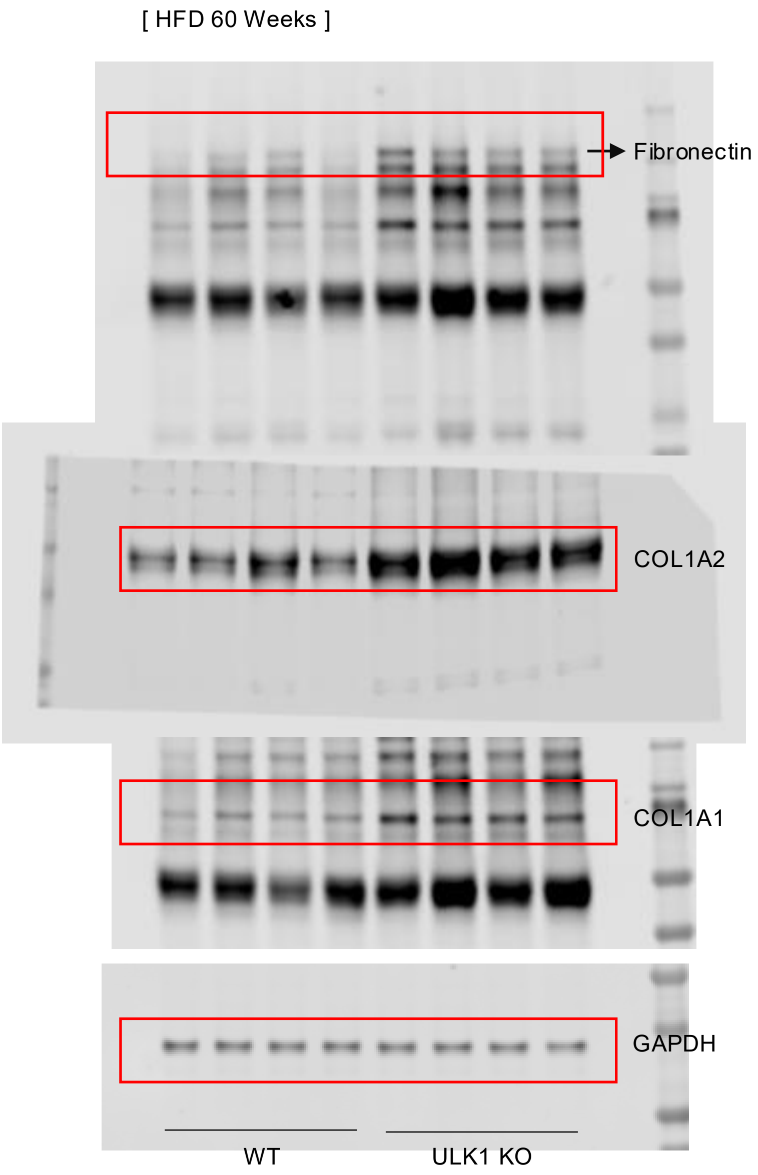

Figure 7. ULK1 deficiency represses NRF2 signaling, promotes oxidative stress and hepatic inflammation through NCOA3-dependent mechanisms.

(A)

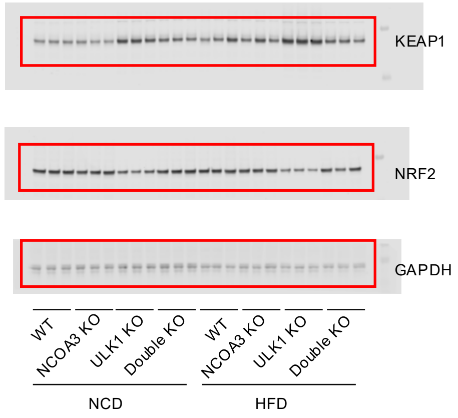

(C)

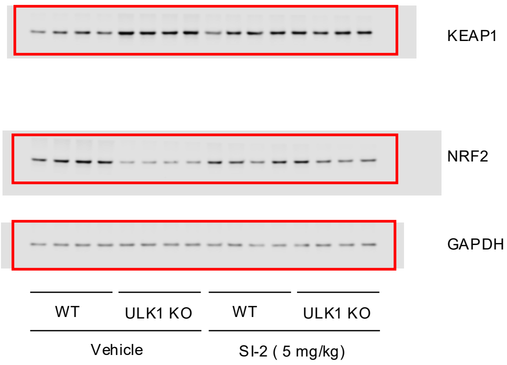

Supplementary Figure 1. Related to Figure 1. ATG protein levels in livers of high-fat-fed mice and autophagy flux when ULK1 is silenced in cultured hepatocytes.

(A)

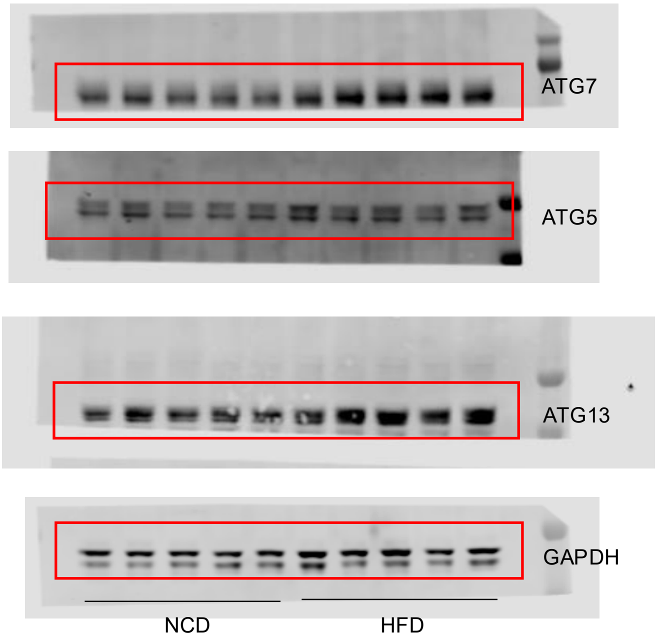

(C)

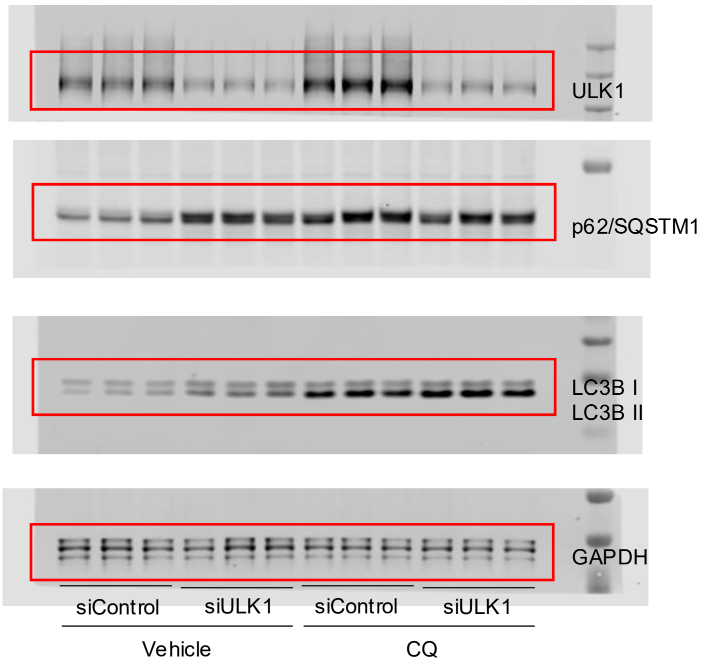

Supplementary Figure 3. Related to Figure 2. Hepatic ULK1 deficiency impairs glucose tolerance, promotes insulin resistance and increases hepatic gluconeogenesis.

(C)

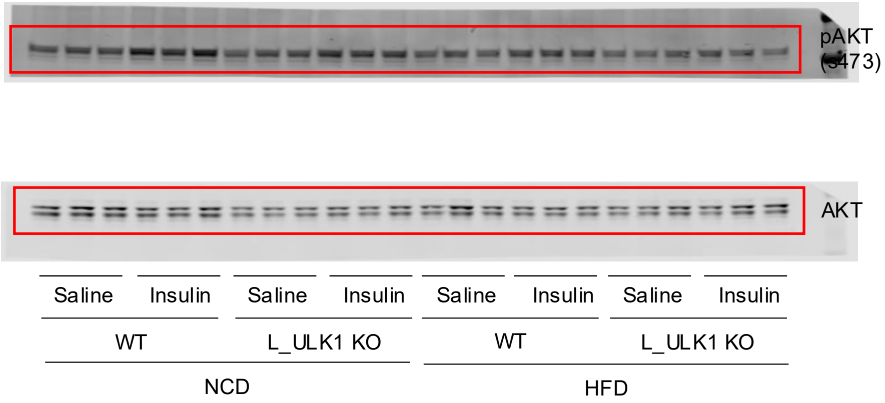

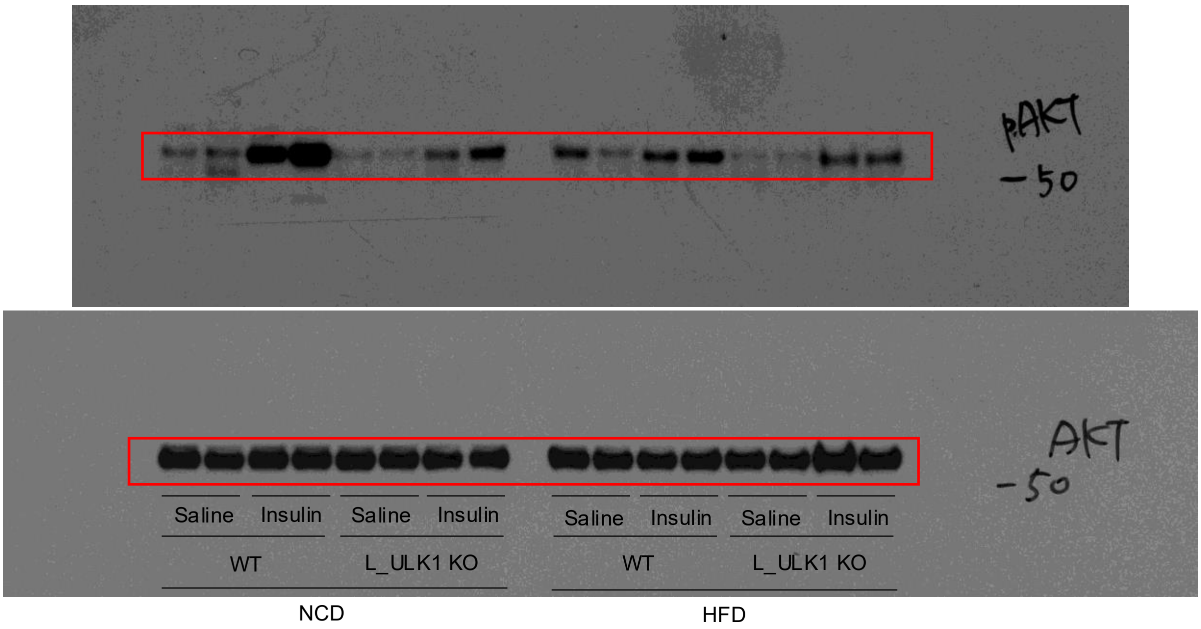

Supplementary Figure 5. Related to Figure 2. Knockout of ULK1 and ULK2 in hepatocytes phenocopies liver-specific ULK1 knockout.

(C)

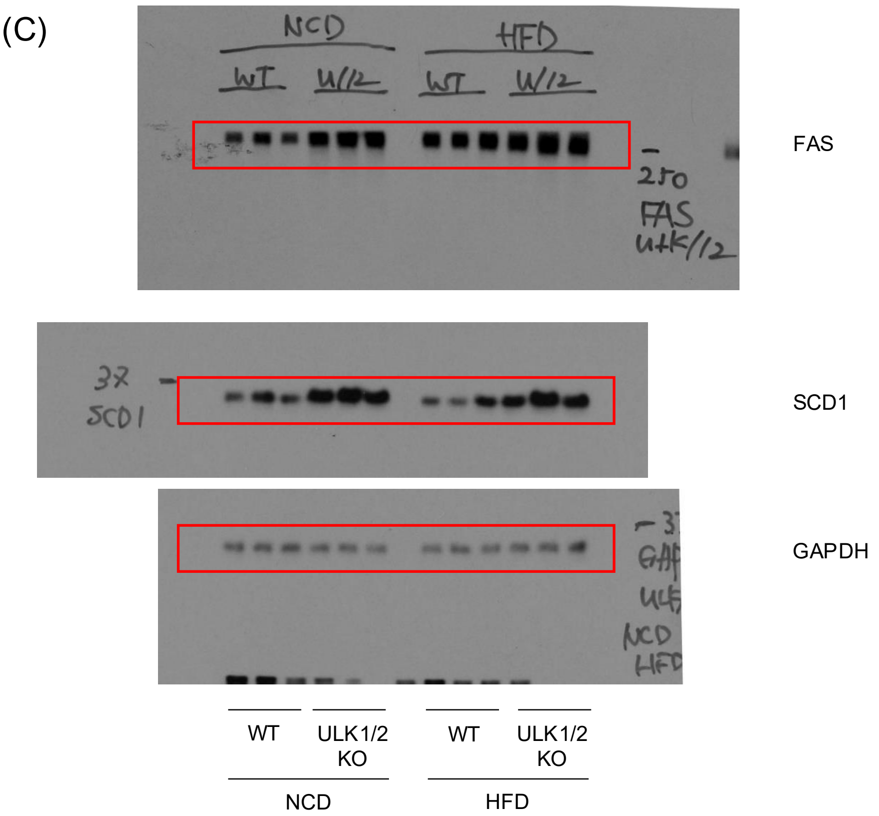

Supplementary Figure 6. Related to Figure 2. Deficiency of ULK1 in hepatocytes increases triglyceride secretion.

(D)

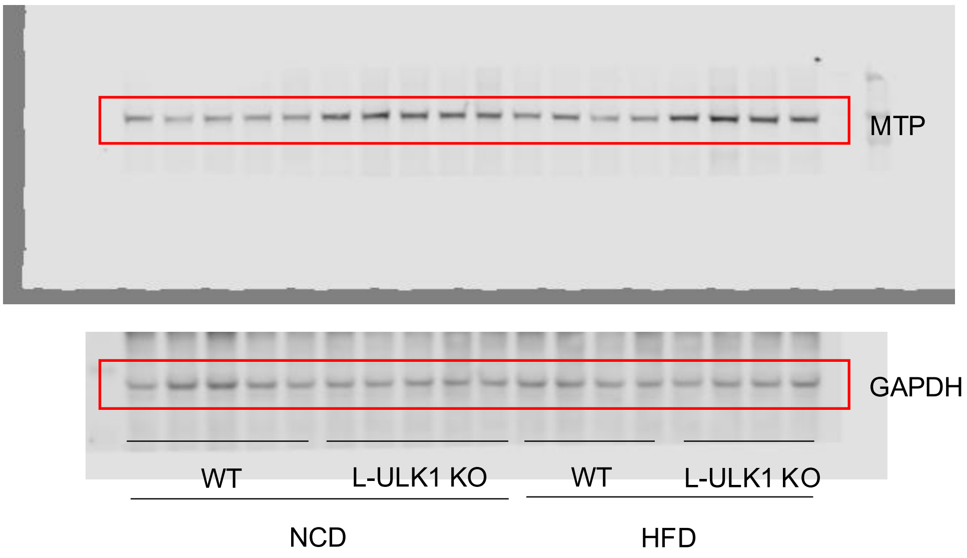

Supplemental figure 10. Related to Figure 4. Effect of site-directed mutagenesis of putative ULK1 phosphorylation sites on lipogenic protein expression in cultured hepatocytes.

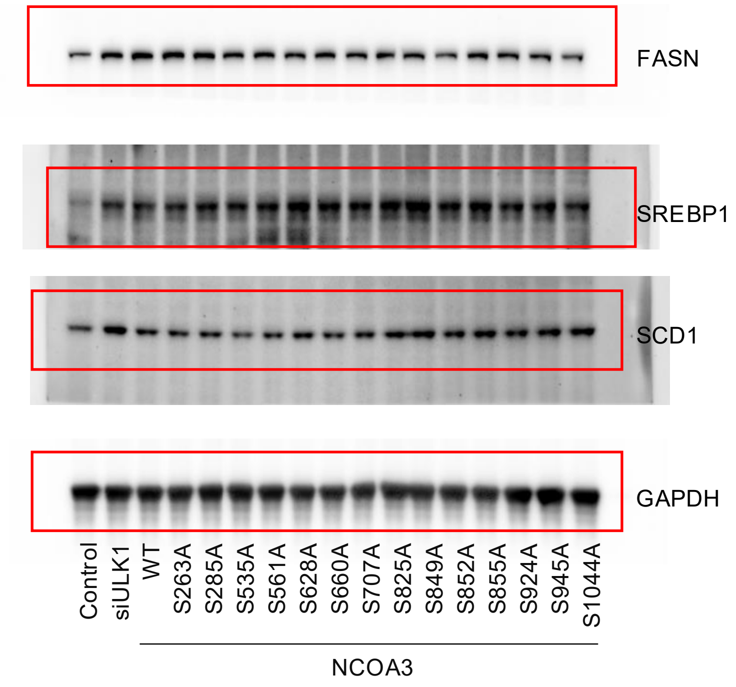

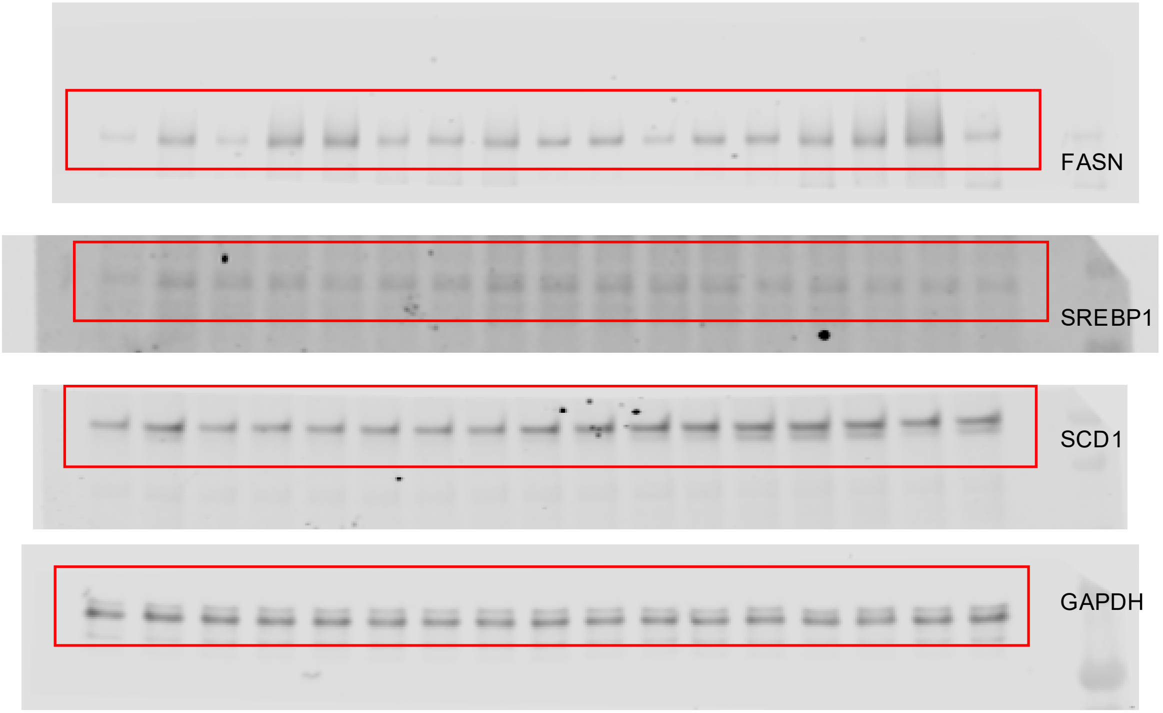

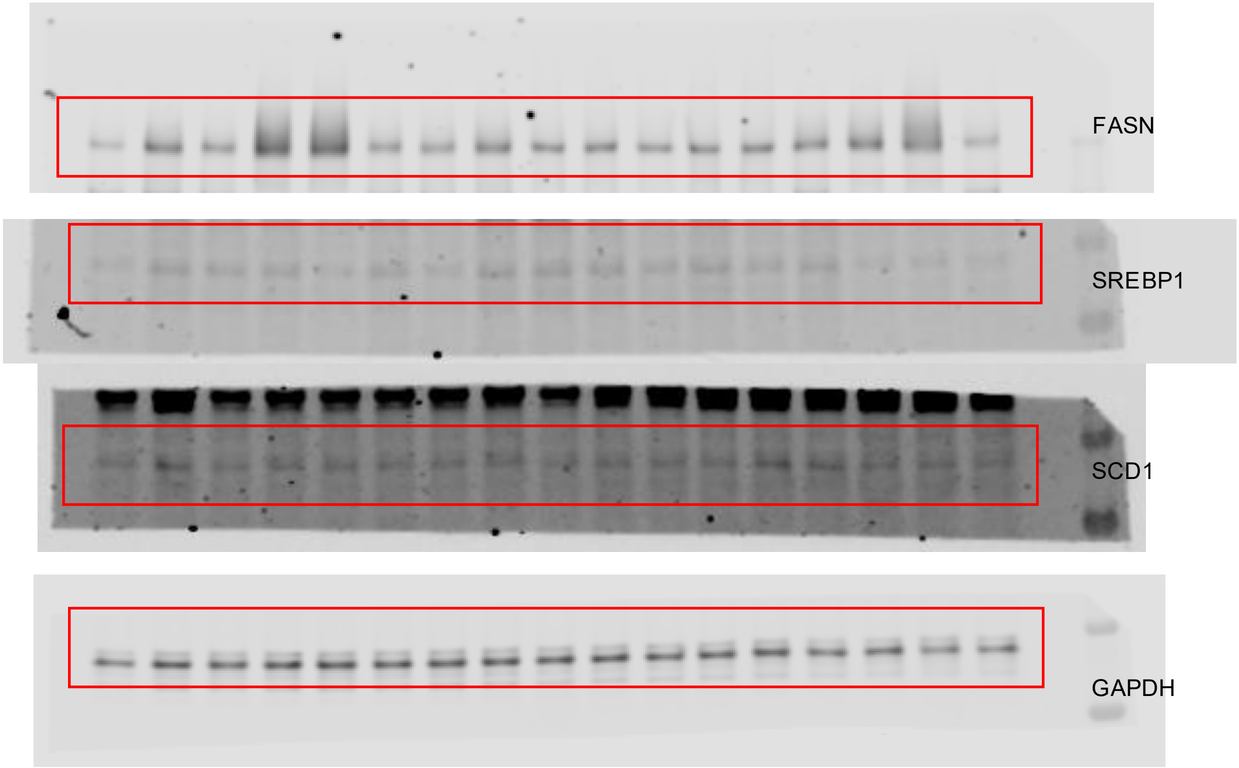

Supplemental figure 12. Related to Figure 5. In cultured hepatocytes, inhibition of NCOA3 by siRNA or by the pharmacological inhibitor SI-2, blocks the induction of lipogenic genes caused by ULK1 deficiency.

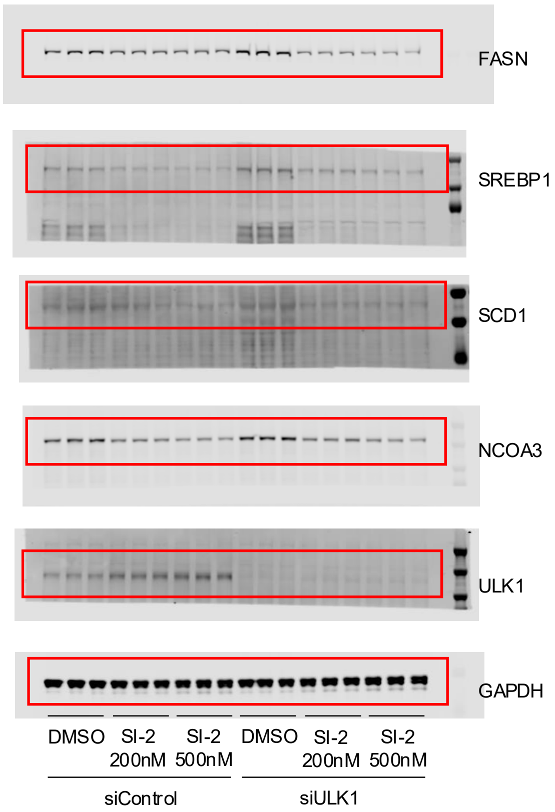

Supplement: Unedited blot and gel images [file jci-136-191101-s166.pdf]
